# The fast and efficient KI/H_2_O_2_ mediated 2-sulfonylation of indoles and *N*-methylpyrrole in water†

**DOI:** 10.1039/c8ra09367a

**Published:** 2018-12-12

**Authors:** Jun Zhang, Zhong Wang, Lingjuan Chen, Yan Liu, Ping Liu, Bin Dai

**Affiliations:** School of Chemistry and Chemical Engineering, The Key Laboratory for Green Processing of Chemical Engineering of Xinjiang Bingtuan, Shihezi University Shihezi City 832004 China liuyan1979810@aliyun.com liuping1979112@aliyun.com +86 0993 2057270 +86 0993 2057213

## Abstract

The rapid and efficient KI/H_2_O_2_-mediated 2-sulfonylation of substituted indoles and *N*-methylpyrrole was established. The corresponding 2-sulfonylation products are synthesized from readily available sulfur sources, namely arylsulfonyl hydrazides, 4-methylbenzenesulfinic acid and sodium 4-methylbenzenesulfinate in good to excellent yields in only 5 min. Moreover, the aqueous solution of hydrogen peroxide is used as both oxidant and solvent. Mechanistic studies demonstrated that 2,3-diiodoindoline was the main sulfonylation intermediate.

## Abstract 2

The rapid and efficient KI/H_2_O_2_-mediated 2-sulfonylation of substituted indoles and *N*-methylpyrrole was established.

## Graphical abstract

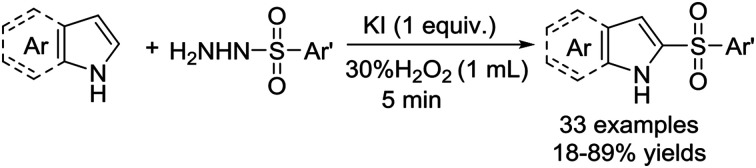

## Introduction

Indole is a widely used building block and is found in many natural products, pharmaceuticals and fine chemicals.^1^ 2-Sulfonylindoles are an important class of indole derivatives due to the sulfonyl moiety which can enhance bioactivity^2^ and acts as a useful vehicle for the development of new strategies for synthesis.^3^ Generally, 2-sulfonylindoles are synthesized *via* the oxidation of the corresponding arylthioindoles.^4^ In recent years, a series of simple, efficient and direct 2-sulfonylations of indoles with sodium sulfinates have been reported for the synthesis of 2-arylsulfonyl indoles. For example, Deng and co-workers developed the I_2_-catalyzed 2-sulfonylation of indoles using sodium sulfinates, with TBHP as the oxidant and HOAc as the solvent.^5^ Around the same time Kuhakarn and co-workers proposed a similar catalytic process in the absence of oxidants.^6^ This provoked the development of numerous catalytic systems, such as NH_4_I–TBHP in HOAc^7^ and KI-oxones (2KHSO_5_–KHSO_4_–K_2_SO_4_) in H_2_O.^8^ Recently, Yu and co-workers presented the electrochemical-sulfonylation of 1*H*-indoles using sodium sulfinates under chemical oxidant-free conditions with TBAI as the catalyst.^9^ In addition, sulfonyl hydrazides are environmentally friendly sulfur sources, and have been extensively employed in organic reaction because they are stable, readily accessible, odor-free.^10^ Barman and co-workers reported the elegant sulfonylation of sulfonyl hydrazides and indoles using TBHP/I_2_ as the catalyst and DCE as solvent.^11^ However, drawbacks still remain, such as prolonged reaction times, the use of organic solvents and large amounts of acid, non-green oxidant, and a relatively expensive iodine source. Therefore, a more environmentally benign protocol for the synthesis of 2-sulfonylindoles is highly desirable. Herein, we report a rapid and efficient KI-mediated 2-sulfonylation of substituted indoles and *N*-methylpyrrole using 30% H_2_O_2_ solution as both oxidant and solvent. Importantly, this transformation can be completed within 5 min and features a broad substrate scope.

## Results and discussion

As our model reaction for the optimization study 1*H*-indole (1a, 0.5 mmol) and *p*-toluenesulfonyl hydrazide (TsNHNH_2_, 2a, 1.0 mmol) were used (Table 1). When the reaction was first conducted using 10% KI and 1 equiv. of H_2_O_2_ in HOAc at 60 °C for 2 h, 2-tosyl-1*H*-indole 3a was obtained in only 15% yield (entry 1). With the addition of EtOH as the solvent product 3a was isolated in 20% yield (entry 2). However, by increasing the KI content to 20%, and with the slow addition of 30% H_2_O_2_ solution to the reaction, we found that the 2-sulfonylation of indole can occur rapidly resulting in 53% yield of product 3a in only 5 min (entry 3). The main reason for the increase in yield may be that a large amount of heat is released due to the addition of hydrogen peroxide to the mixture of 1*H*-indole and TsNHNH_2_ in absence of any other solvent, and the high temperature generated promotes the progress of the 2-sulfonylation. Moreover, by increasing the amount of KI, the reaction yield rose significantly (entries 4 and 5). In the case of altering the substrate ratio of compounds 1a and 2a, no significant change in the reaction yield was observed (entries 6 and 7). When 70% TBHP was used as the oxidant product 3a was produced in 65% yield (entry 8). A similar yield was observed when NH_4_I was employed as the iodine source, furnishing product 3a in 69% yield (entry 9). Since KI is substantially greener and has lower-cost, it was selected as the iodine source and H_2_O_2_ as the oxidant. Finally, by using excess KI, 1.0 and 1.2 equiv. of KI, the reaction yield reached 81% and 83% of compound 3a, respectively (entries 10 and 11). Meanwhile, we also tried the reaction at room temperature using ethanol as a solvent, but only to give a yield of 63% (entry 12). On the basis of the above experiments, the optimized reaction conditions are as follows: 1*H*-indole (1a, 0.5 mmol), TsNHNH_2_ (2a, 1 mmol), KI (1.0 equiv.), H_2_O_2_ (1 mL), for 5 min under air.

**Table 1:** Optimization of the reaction conditions^a^

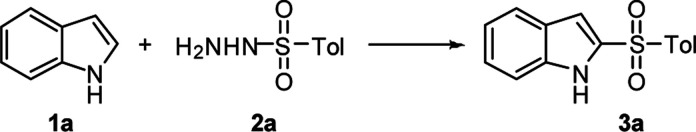				
Entry	Catalyst	Oxidation/solvent	Time (min)	Yield^b^ (%)
1^c^	KI (10%)	H_2_O_2_ (1 equiv.)/HOAc(1 mL)	120	15
2^c^	KI (10%)	H_2_O_2_ (2 equiv.)/EtOH(1 mL)	120	20
3	KI (20%)	H_2_O_2_ (1 mL)/—	5	53
4	KI (40%)	H_2_O_2_ (1 mL)/—	5	58
5	KI (60%)	H_2_O_2_ (1 mL)/—	5	71
6^d^	KI (60%)	H_2_O_2_ (1 mL)/—	5	60
7^e^	KI (60%)	H_2_O_2_ (1 mL)/—	5	48
8	KI (60%)	TBHP (1 mL)/—	5	65
9	NH_4_I (60%)	H_2_O_2_ (1 mL)/—	5	69
10	KI (100%)	H_2_O_2_ (1 mL)/—	5	81
11	KI (120%)	H_2_O_2_ (1 mL)/—	5	83
12	KI (100%)	H_2_O_2_ (1 mL)/EtOH(1 mL)	5	63

a Reaction conditions: 1*H*-indole (1a, 0.5 mmol), TsNHNH_2_ (2a, 1 mmol).

b Isolated yield.

c 60 °C.

d 0.50 mmol of 2a was used.

e 0.75 mmol of 1a was used.

With the optimized reaction conditions in hand, we next investigated the scope of the reaction with a series of indoles, which are summarized in Table 2. The electronic nature of the substituents on the indole was found to have a considerable effect on the reaction yield. Electron-donating substituents (1-Me, 3-Me, 5-Me, 7-Me, 4-MeO, 5-MeO, and 7-MeO) gave better reactivity and provided the corresponding 2-sulfonylated products 3a, 3b, and 3e–j in 78–89% yields. In addition, 4-(benzyloxy)-1*H*-indole furnished product 3k in 60% yield. Whereas, 6-bromo- and 7-bromoindoles gave products 3l and 3m in 78% and 52% yield, respectively. To our delight, 2-methyl-1*H*-indole showed good reactivity and C-3 regioselectivity, providing product 3n in 88% yield. Unfortunately, when 1-ethyl or 1-benzyl-substituted indole was used as the reactant, the desired product 3c or 3d was obtained in a low yield.

**Table 2:** KI/H_2_O_2_-mediated 2-sulfonylation of substituted indoles and TsNHNH_2_^a^

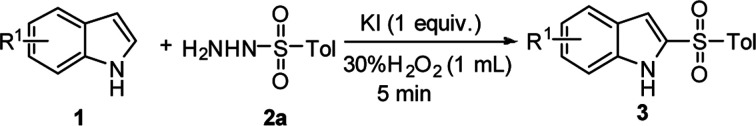
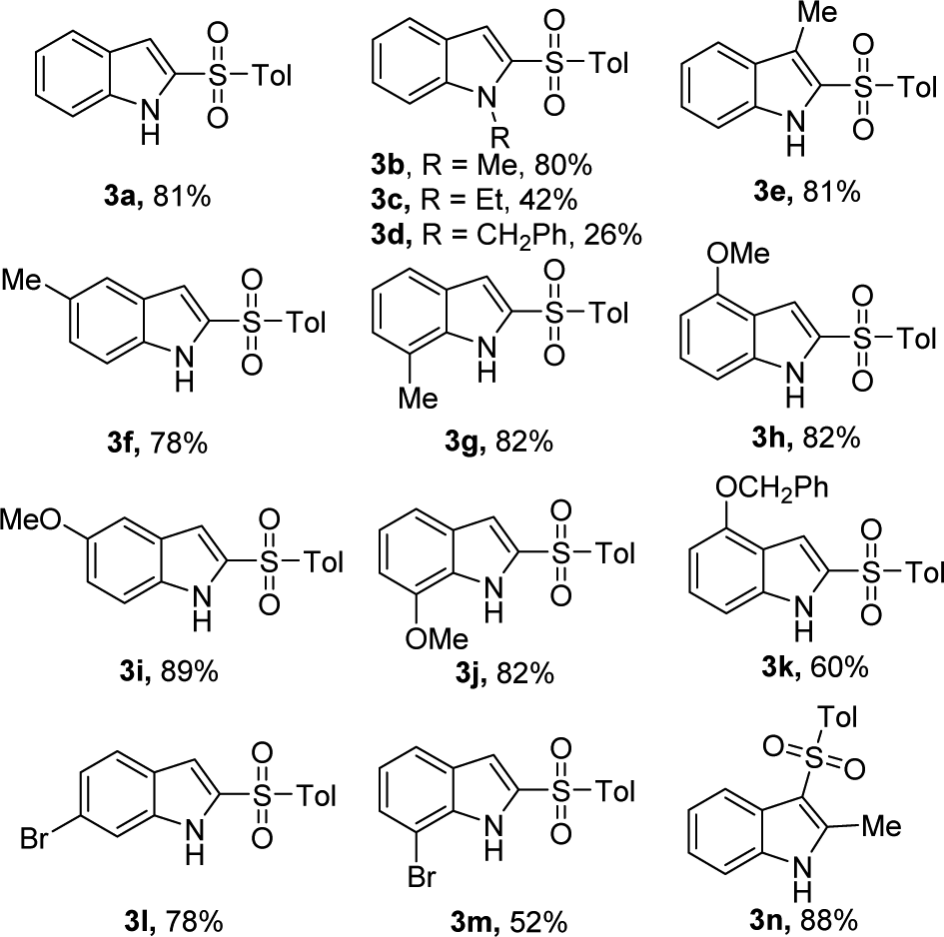

a Reaction conditions: 1 (0.5 mmol), 2a (1.0 mmol), KI (1 equiv.), H_2_O_2_ (1 mL), reaction time 5 min. The yields of isolated products are given.

Encouraged by the above results, we further explored our sulfonylation method using a variety of arylsulfonyl hydrazides with 1*H*-indole 1a under optimum conditions (Table 3). The reaction of benzenesulfonyl hydrazide with 1*H*-indole gave product 3o in only 55% yield. A similar result was obtained for the substituted benzenesulfonyl hydrazide bearing electron-withdrawing groups 4-F, 4-Br, 4-CF_3_. However, 4-Cl or 4-NO_2_ substituted substrates were converted into 2-sulfonylated products 3q and 3t in good yields. Electron-donating substrates 4-^*t*^Bu, and 4-OMe gave 2-sulfonylated products 3u and 3v in 76% and 51% yield, respectively. Furthermore, the method was also extended to 2-naphthylsulfonyl hydrazide, producing product 3w in 81% yield.

**Table 3:** KI/H_2_O_2_-mediated 2-sulfonylation of 1*H*-indole and arylsulfonyl hydrazides^a^

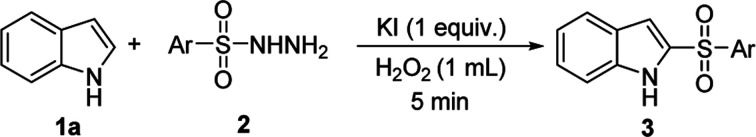
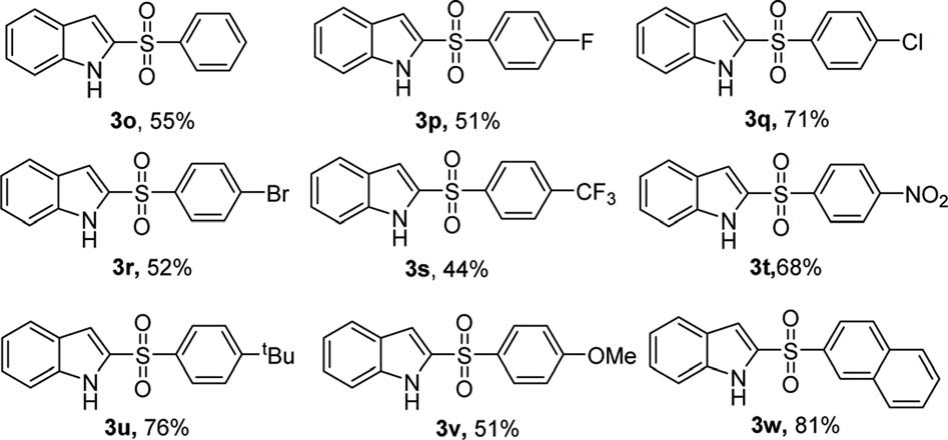

a Reaction conditions: 1a (0.5 mmol), 2 (1.0 mmol), KI (1 equiv.), H_2_O_2_ (1 mL), reaction time 5 min. The yields of isolated products are given.

In our scope studies, 4-methylbenzenesulfinic acid and sodium 4-methylbenzenesulfinate were also examined under the optimized conditions, as shown in Table 4. Weather electron-donating or withdrawing groups were employed the reactions performed well, giving the desired products in high yields. In this case, the electronic nature of the substituent had no significant influence on the reactivity. Compared with the reported methods,^5–9^ operational simplicity, short reaction time and good yield are the key advantages of this protocol.

**Table 4:** 2-Sulfonylation of 4-methylbenzenesulfinic acid or sodium 4-methylbenzenesulfinate as sulfur sources^a^

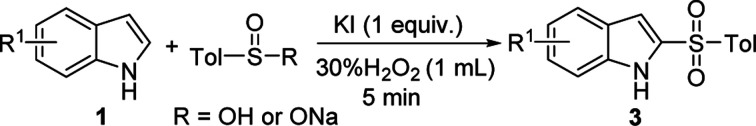
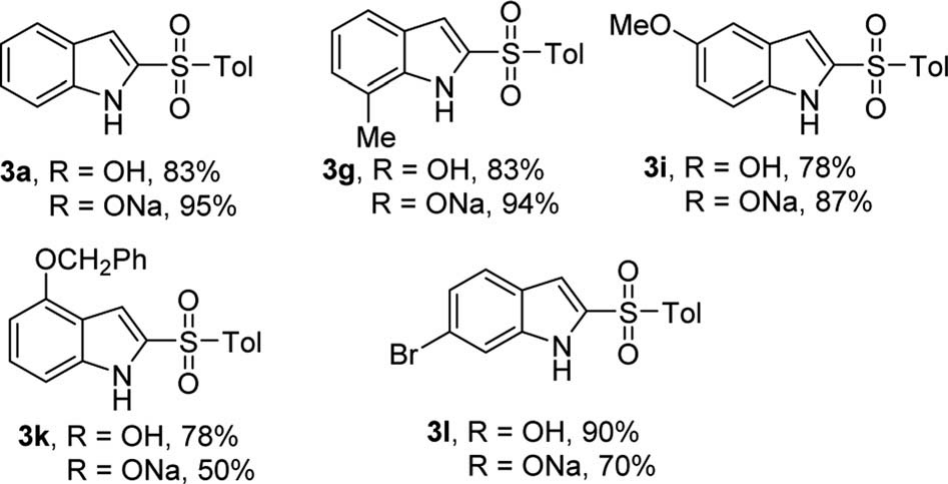

a Reaction conditions: 1a (0.5 mmol), 4-methylbenzenesulfinic acid or sodium 4-methylbenzenesulfinate (1.0 mmol), KI (1 equiv.), H_2_O_2_ (1 mL), reaction time 5 min (0.5 mL HOAc was added, when sodium 4-methylbenzenesulfinate is used as sulfur source). The yields of isolated products are given.

To the best of our knowledge, the 2-sulfonylation of *N*-methylpyrrole has rarely been reported, with unstable sulfur sources and complex reaction conditions often required.^12,13^ Gratifyingly, various arylsulfonyl hydrazides showed good results with *N*-methylpyrrole, giving the desired products 5a–i in 42–57% yields (Table 5). We also found that when 4-methylbenzenesulfinic acid was used as the sulfur source, the reaction yield (5b) significantly improved. In addition, 1*H*-pyrrole as a substrate reacted with *p*-toluenesulfonyl hydrazide to afford the desired product 5b′ in only 18% yield.

**Table 5:** KI/H_2_O_2_-mediated 2-sulfonylation of *N*-methylpyrrole^a^

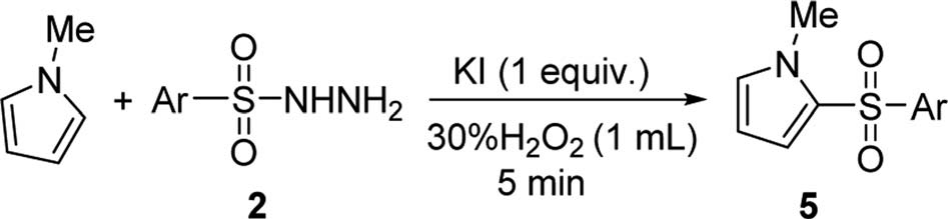
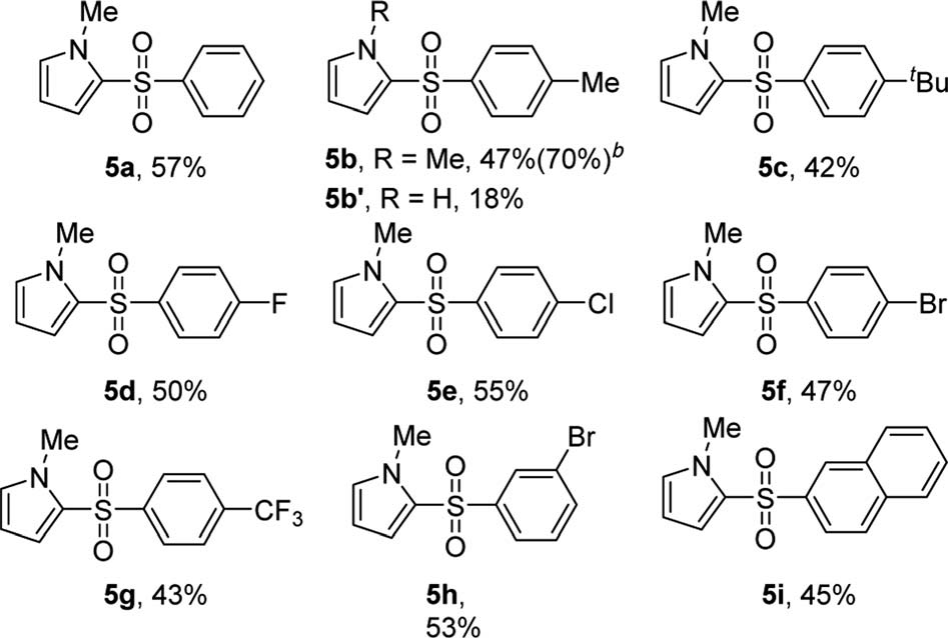

a Reaction conditions: *N*-methylpyrrole (0.5 mmol), 2 (1.0 mmol), KI (1 equiv.) in H_2_O_2_ (1 mL), reaction time 5 min. The yields of isolated products are given.

b 4-Methylbenzenesulfinic acid as sulfur source.

**Scheme 1:**
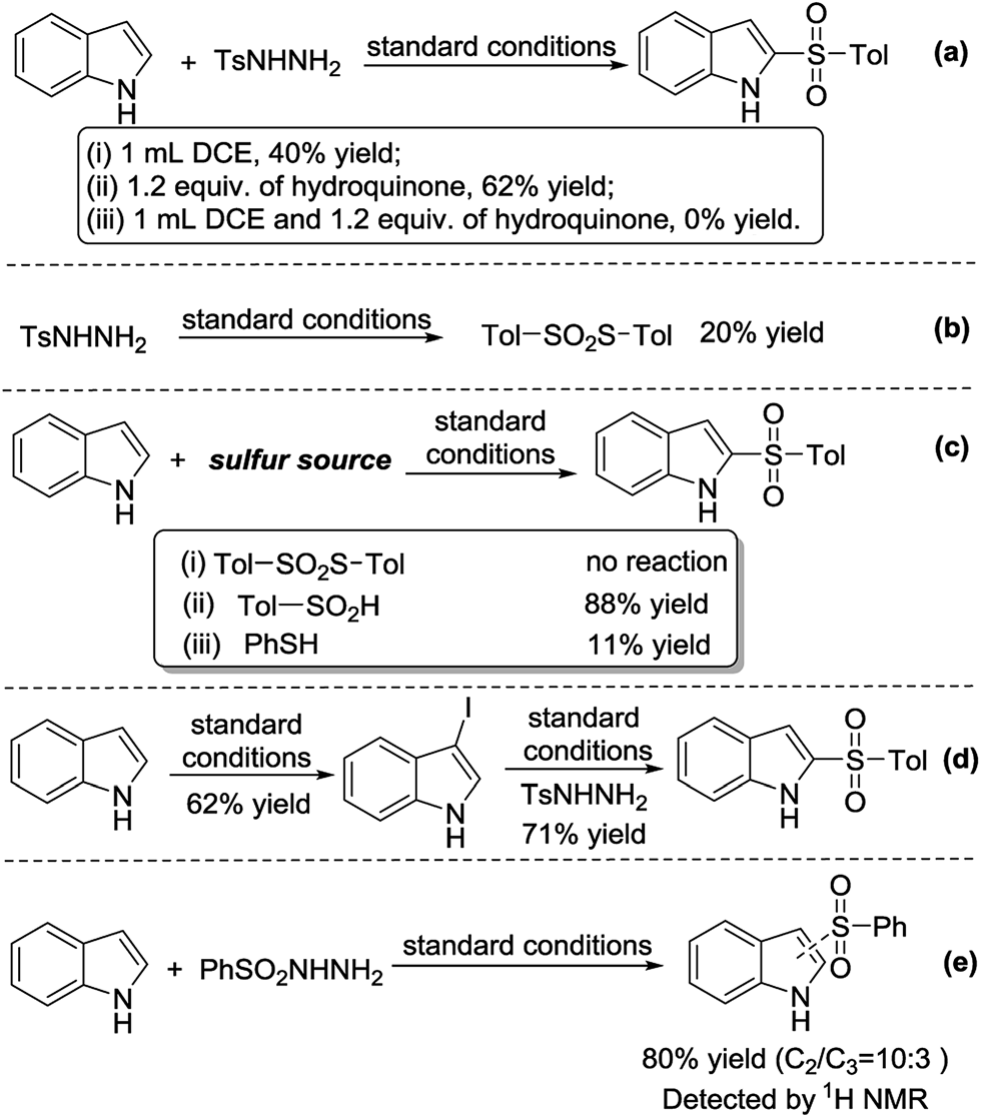
Control experiments.

A series of experiments were conducted to uncover the reaction mechanism. In order to confirm that the reaction was radical, a radical scavenger, 1.2 equiv. of hydroquinone, was added into the reaction mixture. Dichloroethane was also added to aid in solubility of the scavenger. We found that the reaction did not proceed; hence, the reaction was radical (Scheme 1a). In the absence of 1*H*-indole, *p*-toluenesulfonyl hydrazide underwent a self-coupling reaction to form *S-p*-tolyl 4-methylbenzenesulfonothioate (Scheme 1b). However, when *S-p*-tolyl 4-methylbenzenesulfonothioate was used as the sulfur source, the 2-sulfonylation reaction did not proceed. This result indicates that *S-p*-tolyl 4-methylbenzenesulfonothioate is not an intermediate of the reaction. Subsequently, we tested other sulfur sources, such as sodium 4-methylbenzenesulfinate and thiophenol, giving the 2-sulfonylated product under standard conditions in 88% and 11% yield, respectively (Scheme 1c). 1*H*-Indole underwent iodination to form 3-iodo-1*H*-indole in the absence of *p*-toluenesulfonyl hydrazide in 62% yield, and 3-iodo-1*H*-indole reacted with *p*-toluenesulfonyl hydrazide to give 3a in 71% yield under standard conditions (Scheme 1d). In addition, we found an important experimental fact that benzenesulfonyl hydrazide reacted with 1*H*-indole to afford C_2_- and C_3_-sulfonylation products, which were detected by ^1^H NMR in a ratio of 10 : 3 (80% conversion, Scheme 1e).

**Scheme 2:**
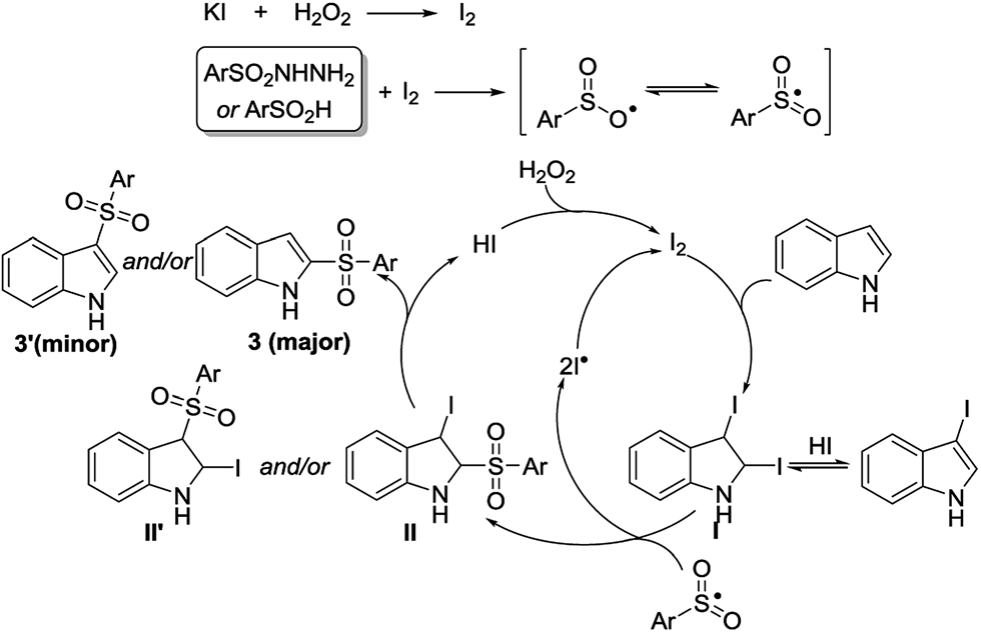
Proposed mechanism.

According to the above results, as well as support from previous reports,^5–9,11^ a plausible radical mechanism is proposed (Scheme 2). KI is first oxidized by H_2_O_2_ to form molecular iodine, which reacts quickly with *p*-toluenesulfonyl hydrazide to produce the active sulfonyl radical. Meanwhile, the addition of molecular iodine to indole occurs and generates a key intermediate I (2,3-diiodoindoline). Subsequently, intermediate I reacts with the sulfonyl radical to produce intermediate II, II′, and iodine radical. Then, HI elimination takes place to provide 2- or 3-sulfonylindole. Meanwhile, the molecular iodine can be re-generated *via* the coupling of two iodine radicals or the oxidation of HI by H_2_O_2_. This mechanism provides a reasonable explanation for the generation of C_2_- and/or C_3_-sulfonylation products (Scheme 1e), and even product 3n (Table 2).

## Conclusions

In summary, we have developed a simple KI-mediated 2-sulfonylation of substituted indoles and *N*-methylpyrrole using 30% H_2_O_2_ solution as the oxidant and the solvent in presence of various sulfur sources. This method provides a fast and efficient approach to produce diversified 2-sulfonylation products in good to excellent yields within 5 min. This protocol has great advantages including short reaction time, green oxidant and solvent, cheap iodine source, avoiding the use of acid, and broad substrate scope. Notably, 2,3-diiodoindoline maybe the main sulfonylation intermediates in this transformation. Studies to further investigate this catalytic system to other related reactions are currently underway in our laboratory.

## Experimental

### Materials and instruments

Chemicals were obtained commercially and used as received. All products were isolated by short chromatography on a silica gel (200–300 mesh) column using petroleum ether (60–90 °C) and ethyl acetate. ^1^H NMR and ^13^C NMR spectra were recorded on a Bruker Avance III HD 400 MHz spectrometer in CDCl_3_ solution. All chemical shifts were reported in ppm (*δ*) relative to the internal standard TMS (0 ppm). ESI-mass spectrum was measured on an Agilent 6210 ESI/TOF MS.

### General procedure for a KI/H_2_O_2_ mediated 2-sulfonylation of indoles and *N*-methylpyrrole in water

A mixture of substituted indole or *N*-methylpyrrole (0.5 mmol), sulfur source (1.0 mmol, arylsulfonyl hydrazides or 4-methylbenzenesulfinic acid or sodium 4-methylbenzenesulfinate + 0.5 mL HOAc) and KI (0.5 mmol) was stirred at room temperature under air. Subsequently, 30% H_2_O_2_ solution (1 mL) was slowly added to the above system (note: a large amount of heat is generated during this process) and the reaction stirred for an additional 5 min. The solution was quenched the saturated solution of sodium thiosulfate (5 mL) and extracted with EtOAc (3 × 10 mL). The combined EtOAc extracts were dried over anhydrous MgSO_4_, filtered and concentrated under reduced pressure. The crude residue was purified by flash column chromatography on silica gel using PE/EtOAc as the eluent.

### The characterization data of products

#### 4-Methoxy-2-tosyl-1*H*-indole (3h)

White solid, 123 mg, 82% yield, mp 155–158 °C; ^1^H NMR (400 MHz, CDCl_3_) *δ* 8.90 (s, 1H), 7.87 (d, *J* = 8.4 Hz, 2H), 7.28 (d, *J* = 7.1 Hz, 3H), 7.23 (d, *J* = 8.0 Hz, 1H), 6.99 (d, *J* = 8.4 Hz, 1H), 6.52 (d, *J* = 7.8 Hz, 1H), 3.92 (s, 3H), 2.38 (s, 3H); ^13^C NMR (101 MHz, CDCl_3_) *δ* 154.65, 144.52, 130.06, 127.45, 127.20, 106.80, 105.11, 100.55, 55.52, 21.72; HRMS(ESI): *m*/*z* calcd for C_16_H_15_NO_3_S (M)^+^: 301.0767, found: 301.0763.

#### 7-Methoxy-2-tosyl-1*H*-indole (3j)

White solid, 123 mg, 82% yield, mp 149–151 °C; ^1^H NMR (400 MHz, CDCl_3_) *δ* 9.00 (s, 1H), 7.86 (d, *J* = 8.3 Hz, 2H), 7.28 (s, 2H), 7.23 (d, *J* = 8.2 Hz, 1H), 7.16–7.03 (m, 3H), 6.73 (d, *J* = 7.7 Hz, 1H), 3.95 (s, 3H), 2.38 (s, 3H); ^13^C NMR (101 MHz, CDCl_3_) *δ* 146.65, 144.52, 138.77, 134.40, 130.04, 127.47, 122.15, 114.85, 109.09, 104.80, 55.60, 21.71; HRMS(ESI): *m*/*z* calcd for C_16_H_15_NO_3_S (M)^+^: 301.0767, found: 301.0769.

#### 4-(Benzyloxy)-2-tosyl-1*H*-indole (3k)

White solid, 113 mg, 60% yield, mp 136–139 °C; ^1^H NMR (400 MHz, CDCl_3_) *δ* 8.97 (s, 1H), 7.87 (d, *J* = 8.3 Hz, 2H), 7.47 (d, *J* = 7.2 Hz, 2H), 7.37 (dt, *J* = 24.0, 7.0 Hz, 4H), 7.28 (s, 1H), 7.22 (t, *J* = 8.1 Hz, 1H), 6.58 (d, *J* = 7.8 Hz, 1H), 5.18 (s, 2H), 2.38 (s, 3H); ^13^C NMR (101 MHz, CDCl_3_) *δ* 153.74, 138.79, 138.54, 136.92, 133.16, 130.07, 128.72, 128.15, 127.44, 107.00, 105.38, 101.85, 70.11, 21.72; HRMS(ESI): *m*/*z* calcd for C_22_H_19_NO_3_S (M)^+^: 377.1080, found: 377.1075.

#### 6-Bromo-2-tosyl-1*H*-indole (3l)

Brown solid, 136 mg, 78% yield, mp 181–182 °C; ^1^H NMR (400 MHz, CDCl_3_) *δ* 9.25 (s, 1H), 7.88 (d, *J* = 8.3 Hz, 2H), 7.61–7.48 (m, 2H), 7.33–7.27 (m, 3H), 2.39 (s, 3H); ^13^C NMR (101 MHz, CDCl_3_) *δ* 144.99, 138.28, 137.75, 135.32, 130.24, 127.50, 126.01, 125.30, 123.96, 119.78, 115.35, 108.91, 21.77; HRMS(ESI): *m*/*z* calcd for C_15_H_12_BrNNaO_2_S (M + Na)^+^: 371.9664, found: 371.9653.

#### 7-Bromo-2-tosyl-1*H*-indole (3m)

Dark red solid, 91 mg, 52% yield, mp 155–158 °C. ^1^H NMR (400 MHz, CDCl_3_) *δ* 8.86 (s, 1H), 7.91 (d, *J* = 8.3 Hz, 2H), 7.60 (d, *J* = 8.1 Hz, 1H), 7.51–7.47 (m, 1H), 7.33 (d, *J* = 8.1 Hz, 2H), 7.22 (d, *J* = 2.2 Hz, 1H), 7.06 (t, *J* = 7.8 Hz, 1H), 2.41 (s, 3H). ^13^C NMR (101 MHz, CDCl_3_) *δ* 144.87, 138.14, 135.77, 135.57, 130.10, 128.20, 128.02, 127.51, 122.67, 121.87, 109.60, 105.32, 21.64; HRMS(ESI): *m*/*z* calcd for C_15_H_12_BrNNaO_2_S (M + Na)^+^: 371.9664, found: 371.9661.

#### 2-Methyl-3-tosyl-1*H*-indole (3n)

White solid, 125 mg, 88% yield; mp 179–181 °C. ^1^H NMR (400 MHz, CDCl_3_) *δ* 9.26 (s, 1H), 7.97 (d, *J* = 8.5 Hz, 1H), 7.83 (d, *J* = 8.3 Hz, 2H), 7.24–7.10 (m, 5H), 2.64 (s, 3H), 2.32 (s, 3H). ^13^C NMR (101 MHz, CDCl_3_) *δ* 143.38, 141.52, 141.19, 134.54, 129.75, 126.15, 125.40, 123.10, 122.17, 119.28, 111.42, 111.27, 21.57, 13.04. HRMS(ESI): *m*/*z* calcd for C_16_H_16_NO_2_S (M + H)^+^: 286.0896, found: 286.0900.

#### 2-((4-Nitrophenyl)sulfonyl)-1*H*-indole (3t)

Light yellow solid, 102 mg, 68% yield, mp 129–132 °C; ^1^H NMR (400 MHz, CDCl_3_) *δ* 8.90 (s, 1H), 8.36–8.34 (m, 1H), 8.33 (d, *J* = 2.1 Hz, 1H), 8.20–8.15 (m, 2H), 7.69 (d, *J* = 8.1 Hz, 1H), 7.46–7.42 (m, 1H), 7.41–7.37 (m, 1H), 7.29 (dd, *J* = 2.1, 0.8 Hz, 1H), 7.22 (ddd, *J* = 8.0, 6.8, 1.1 Hz, 1H); ^13^C NMR (101 MHz, CDCl_3_) *δ* 150.40, 147.18, 137.49, 132.20, 129.24, 128.58, 127.15, 126.92, 124.61, 124.46, 122.97, 122.12, 112.35, 110.85. HRMS(ESI): *m*/*z* calcd for C_14_H_10_N_2_NaO_4_S (M + Na)^+^: 325.0253, found: 325.0251.

#### 2-((4-(*tert*-Butyl)phenyl)sulfonyl)-1-methyl-1*H*-pyrrole (5c)

Pale yellow solid, 58 mg, 42% yield, mp 59–61 °C; ^1^H NMR (400 MHz, CDCl_3_) *δ* 7.80 (d, *J* = 8.7 Hz, 2H), 7.50 (d, *J* = 8.7 Hz, 2H), 7.02 (dd, *J* = 4.0, 1.9 Hz, 1H), 6.75 (t, *J* = 2.2 Hz, 1H), 6.16 (dd, *J* = 4.0, 2.6 Hz, 1H), 3.72 (s, 3H), 1.32 (s, 9H); ^13^C NMR (101 MHz, CDCl_3_) *δ* 156.84, 139.25, 129.56, 128.43, 127.16, 126.33, 118.64, 108.33, 35.79, 35.30, 31.18; HRMS(ESI): *m*/*z* calcd for C_15_H_20_NO_2_S (M + H)^+^: 278.1209, found: 278.1208.

#### 1-Methyl-2-((4-(trifluoromethyl)phenyl)sulfonyl)-1*H*-pyrrole (5g)

Pale yellow solid, 62 mg, 43% yield, mp 66–68 °C; ^1^H NMR (400 MHz, CDCl_3_) *δ* 8.01 (d, *J* = 8.2 Hz, 2H), 7.77 (d, *J* = 8.3 Hz, 2H), 7.09 (dd, *J* = 4.1, 1.9 Hz, 1H), 6.81 (t, *J* = 2.2 Hz, 1H), 6.21 (dd, *J* = 4.1, 2.6 Hz, 1H), 3.73 (s, 3H); ^13^C NMR (101 MHz, CDCl_3_) *δ* 145.96, 134.76, 134.43, 130.61, 127.77, 126.85, 126.53, 126.50, 124.63, 121.92, 119.92, 108.94, 35.87; HRMS(ESI): *m*/*z* calcd for C_12_H_11_F_3_NO_2_S (M + H)^+^: 290.0457, found: 290.0457.

#### 2-((3-Bromophenyl)sulfonyl)-1-methyl-1*H*-pyrrole (5h)

Pale yellow solid, 79 mg, 53% yield, mp 55–58 °C; ^1^H NMR (400 MHz, CDCl_3_) *δ* 8.01 (t, *J* = 1.8 Hz, 1H), 7.81 (d, *J* = 7.9 Hz, 1H), 7.68 (d, *J* = 8.0 Hz, 1H), 7.38 (t, *J* = 7.9 Hz, 1H), 7.05 (dd, *J* = 4.1, 1.9 Hz, 1H), 6.80 (t, *J* = 2.2 Hz, 1H), 6.20 (dd, *J* = 4.1, 2.6 Hz, 1H), 3.72 (s, 3H); ^13^C NMR (101 MHz, CDCl_3_) *δ* 144.26, 136.02, 130.86, 130.36, 130.09, 127.11, 125.78, 123.25, 119.62, 108.77, 35.85; HRMS(ESI): *m*/*z* calcd for C_11_H_10_BrNO_2_S (M)^+^: 298.9688, found: 298.9690.

#### 1-Methyl-2-(naphthalen-2-ylsulfonyl)-1*H*-pyrrole (5i)

Pale yellow solid, 60 mg, 45% yield, mp 60–63 °C; ^1^H NMR (400 MHz, CDCl_3_) *δ* 8.50 (s, 1H), 7.93 (td, *J* = 17.0, 16.6, 7.8 Hz, 3H), 7.80 (dd, *J* = 8.7, 1.9 Hz, 1H), 7.66–7.57 (m, 2H), 7.10 (dd, *J* = 4.0, 1.9 Hz, 1H), 6.75 (t, *J* = 2.2 Hz, 1H), 6.19 (dd, *J* = 4.0, 2.6 Hz, 1H), 3.72 (s, 3H); ^13^C NMR (101 MHz, CDCl_3_) *δ* 139.13, 135.02, 132.30, 129.85, 129.73, 129.47, 129.15, 128.33, 128.06, 127.72, 122.71, 119.14, 108.54, 35.81; HRMS(ESI): *m*/*z* calcd for C_15_H_14_NO_2_S (M + H)^+^: 272.0740, found: 272.0745.

## Conflicts of interest

There are no conflicts to declare.

## Supplementary Material

RA-008-C8RA09367A-s001
