# Supplementary material for: The fast and efficient KI/H2O2 mediated 2-sulfonylation of indoles and N-methylpyrrole in water
Source: RSC Adv. 2018 Dec 12;8(72):41651–6. doi: 10.1039/c8ra09367a (PMC9092009; doi:10.1039/c8ra09367a)
Supplement: RA-008-C8RA09367A-s001 [file RA-008-C8RA09367A-s001.pdf]

Electronic Supplementary Material (ESI) for RSC Advances.
This journal is © The Royal Society of Chemistry 2018

Supporting information

The fast and efficient KI/H_2O_2 mediated 2-sulfonylation of indoles and *N*-methylpyrrole in water

Jun Zhang, Zhong Wang, Lingjuan Chen, Yan Liu*, Ping Liu*, and Bin Dai

School of Chemistry and Chemical Engineering, the Key Laboratory for Green Processing of Chemical Engineering of Xinjiang Bingtuan, Shihezi University, Shihezi City, 832004, China

E-mail: liuyan1979810@aliyun.com and liuping1979112@aliyun.com

Table of Contents

General Information 2
Characterization of compounds 2
^{1}H and ^{13}C NMR spectra 13

General Information

Unless otherwise stated, all solvents and reagents were obtained commercially without further purification, all reactions were carried out in air and the reaction was monitored by TLC. All products were isolated by short chromatography on a silica gel (200–300 mesh) column using petroleum ether (60–90 °C) and ethyl acetate. ^{1}H NMR and ^{13}C NMR spectra were recorded on a Brucker Advance III HD 400 MHz spectrometer in $CDCl_3$ solution. All chemical shifts were reported in ppm (δ) relative to the internal standard TMS (0 ppm). ESI–Mass spectrum was measured on an Agilent 6210 ESI/TOF MS.

Characterization of compounds

2-tosyl-1H-indole (3a)[1]

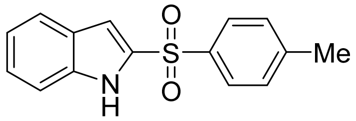

White solid, 110 mg, 81% yield, m.p. 190-192 ^{o}C; ^{1}H NMR (400 MHz, $CDCl_3$) δ 8.91 (s, 1H), 7.88 (d, J = 8.4 Hz, 2H), 7.66 (d, J = 8.8 Hz, 1H), 7.43–7.39 (m, 1H), 7.36–7.27 (m, 3H), 7.20–7.14 (m, 2H), 2.39 (s, 3H); ^{13}C NMR (101 MHz, $CDCl_3$) δ 144.68, 138.68, 137.12, 134.69, 130.12, 127.49, 126.10, 122.81, 121.69, 112.36, 108.98, 21.74.

1-methyl-2-tosyl-1H-indole (3b)[2]

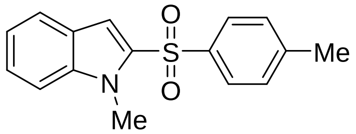

Pale yellow solid, 114 mg, 80% yield, m.p. 124–125 °C; ^{1}H NMR (400 MHz, $CDCl_3$) δ 7.84 (d, J = 8.3 Hz, 2H), 7.69 (d, J = 8.1 Hz, 1H), 7.39–7.28 (m, 5H), 7.21–7.14 (m, 1H), 3.84 (s, 3H), 2.41 (s, 3H); ^{13}C NMR (101 MHz, $CDCl_3$) δ 144.61, 139.56, 138.37, 135.30, 130.07, 127.86, 125.73, 125.31, 122.94, 121.22, 110.54, 110.34, 31.11, 21.74.

1-ethyl-2-tosyl-1H-indole (3c)[2]

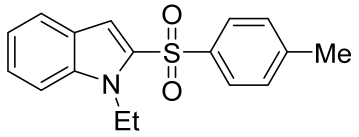

Light yellow liquid, 63 mg, 42% yield. ^{1}H NMR (400 MHz, $CDCl_3$): δ 7.84 (d, J = 6.8 Hz, 2H), 7.69 (d, J = 8.0 Hz, 1H), 7.37-7.27 (m, 5H), 7.20-7.13 (m, 1H), 4.37 (q, J = 7.2 Hz, 2H), 2.39 (s, 2H), 1.16 (t, J = 7.2 Hz, 3H).

1-benzyl-2-tosyl-1H-indole (3d)[2]

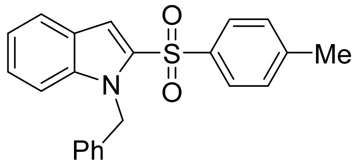

Pale yellow solid, 47 mg, 26% yield. ^{1}H NMR (400 MHz, $CDCl_3$): δ 7.72 (d, J = 8.0 Hz, 1H), 7.62 (d, J = 8.4 Hz, 2H), 7.47 (d, J = 0.8 Hz, 1H), 7.28-7.22 (m, 1H), 7.20-7.07 (m, 3H), 7.07-6.99 (m, 4H), 6.68 (d, J = 7.1 Hz, 2H), 5.64 (s, 2H), 2.28 (s, 3H).

3-methyl-2-tosyl-1H-indole (3e)[2]

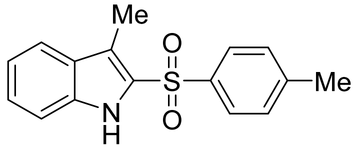

Yellow solid, 115 mg, 80% yield, m.p. 186–187 °C; ^{1}H NMR (400 MHz, $CDCl_3$) δ 9.28 (s, 1H), 7.87 (dd, J = 8.3, 2.6 Hz, 2H), 7.58 (d, J = 8.1 Hz, 1H), 7.39 (d, J = 8.4 Hz, 1H), 7.33–7.23 (m, 3H), 7.17–7.11 (m, 1H), 2.53 (s, 3H), 2.36 (s, 3H); ^{13}C NMR (101 MHz, $CDCl_3$) δ 144.38, 139.09, 136.09, 130.02, 129.52, 128.32, 126.12, 118.51, 112.42, 21.65, 9.02.

5-methyl-2-tosyl-1H-indole (3f)[3]

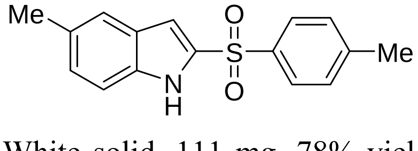

White solid, 111 mg, 78% yield, m.p. 135–137 °C; ^{1}H NMR (400 MHz, $CDCl_3$) δ 8.88 (s, 1H), 7.87 (d, J = 8.4 Hz, 2H), 7.42 (s, 1H), 7.29 (dd, J = 8.3, 5.3 Hz, 3H), 7.15 (dd, J = 8.5, 1.5 Hz, 1H), 7.09–7.07 (m, 1H), 2.41 (s, 3H), 2.38 (s, 3H); ^{13}C

NMR (101 MHz, $CDCl_3$) δ 144.55, 138.80, 135.61, 134.43, 130.07, 128.03, 127.41, 121.98, 112.03, 108.53, 21.72.

7-methyl-2-tosyl-1H-indole (3g)[3]

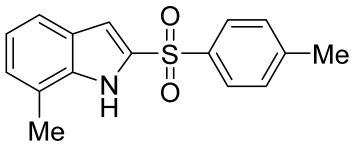

White solid, 116 mg, 82% yield, m.p. 171–173 °C; ^{1}H NMR (400 MHz, $CDCl_3$) δ 9.01 (s, 1H), 7.92 (t, J = 6.5 Hz, 2H), 7.49 (d, J = 7.7 Hz, 1H), 7.29 (d, J = 8.1 Hz, 2H), 7.18 (d, J = 2.2 Hz, 1H), 7.13–7.05 (m, 2H), 2.48 (s, 3H), 2.39 (s, 3H); ^{13}C NMR (101 MHz, $CDCl_3$) δ 144.61, 138.76, 137.21, 134.27, 130.11, 127.44, 126.30, 121.86, 120.26, 109.62, 21.72, 16.89.

4-methoxy-2-tosyl-1H-indole (3h)

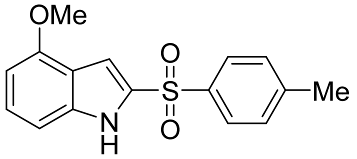

White solid, 123 mg, 82% yield, m.p. 155-158 °C; ^{1}H NMR (400 MHz, $CDCl_3$) δ 8.90 (s, 1H), 7.87 (d, J = 8.4 Hz, 2H), 7.28 (d, J = 7.1 Hz, 3H), 7.23 (d, J = 8.0 Hz, 1H), 6.99 (d, J = 8.4 Hz, 1H), 6.52 (d, J = 7.8 Hz, 1H), 3.92 (s, 3H), 2.38 (s, 3H); ^{13}C NMR (101 MHz, $CDCl_3$) δ 154.65, 144.52, 130.06, 127.45, 127.20, 106.80, 105.11, 100.55, 55.52, 21.72; HRMS(ESI): m/z calcd for $C_{16}H_{15}NO_3S$ $(M)^+$: 301.0767, found: 301.0763.

5-methoxy-2-tosyl-1H-indole (3i)[2]

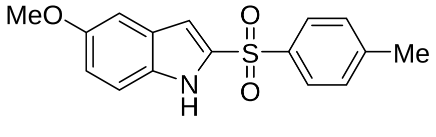

White solid, 134 mg, 89% yield, m.p. 145-147 °C; ^{1}H NMR (400 MHz, $CDCl_3$) δ 8.92 (s, 1H), 7.87 (d, J = 8.4 Hz, 2H), 7.29 (dd, J = 8.5, 4.2 Hz, 3H), 7.08 (dd, J = 2.1, 0.9 Hz, 1H), 7.04–6.97 (m, 2H), 3.82 (s, 3H), 2.39 (s, 3H); ^{13}C NMR (101 MHz, $CDCl_3$) δ 155.26, 144.58, 134.74, 132.48, 130.09, 127.73, 127.41, 117.75, 113.35, 108.52, 102.66, 55.83, 21.73.

7-methoxy-2-tosyl-1H-indole (3j)

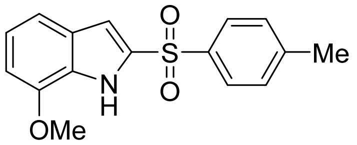

White solid, 123 mg, 82% yield, m.p.149-151 °C; ^{1}H NMR (400 MHz, $CDCl_3$) δ 9.00 (s, 1H), 7.86 (d, J = 8.3 Hz, 2H), 7.28 (s, 2H), 7.23 (d, J = 8.2 Hz, 1H), 7.16–7.03 (m, 3H), 6.73 (d, J = 7.7 Hz, 1H), 3.95 (s, 3H), 2.38 (s, 3H); ^{13}C NMR (101 MHz, $CDCl_3$) δ 146.65, 144.52, 138.77, 134.40, 130.04, 127.47, 122.15, 114.85, 109.09, 104.80, 55.60, 21.71; HRMS(ESI): m/z calcd for $C_{16}H_{15}NO_3S(M)^+$: 301.0767, found: 301.0769.

4-(benzyloxy)-2-tosyl-1H-indole (3k)

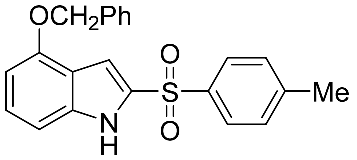

White solid, 113 mg, 60% yield, m.p. 136-139 °C; ^{1}H NMR (400 MHz, $CDCl_3$) δ 8.97 (s, 1H), 7.87 (d, J = 8.3 Hz, 2H), 7.47 (d, J = 7.2 Hz, 2H), 7.37 (dt, J = 24.0, 7.0 Hz, 4H), 7.28 (s, 1H), 7.22 (t, J = 8.1 Hz, 1H), 6.58 (d, J = 7.8 Hz, 1H), 5.18 (s, 2H), 2.38 (s, 3H); ^{13}C NMR (101 MHz, $CDCl_3$) δ 153.74, 138.79, 138.54, 136.92, 133.16, 130.07, 128.72, 128.15, 127.44, 107.00, 105.38, 101.85, 70.11, 21.72; HRMS(ESI): m/z calcd for $C_{22}H_{19}NO_3S(M)^+$: 377.1080, found: 377.1075.

6-bromo-2-tosyl-1H-indole (3l)

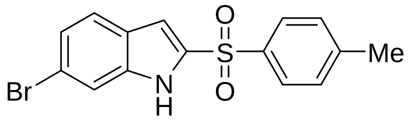

Brown solid, 136 mg, 78% yield, m.p. 181-182 °C; ^{1}H NMR (400 MHz, $CDCl_3$) δ 9.25 (s, 1H), 7.88 (d, J = 8.3 Hz, 2H), 7.61 – 7.48 (m, 2H), 7.33 – 7.27 (m, 3H), 2.39 (s, 3H); ^{13}C NMR (101 MHz, $CDCl_3$) δ 144.99, 138.28, 137.75, 135.32, 130.24, 127.50, 126.01, 125.30, 123.96, 119.78, 115.35, 108.91, 21.77; HRMS(ESI): m/z calcd for $C_{15}H_{12}BrNNaO_2S(M+Na)^+$: 371.9664, found: 371.9653.

7-bromo-2-tosyl-1H-indole (3m)

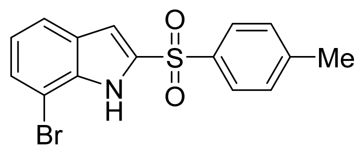

Dark red solid, 91 mg, 52% yield, m.p. 155-158 °C; ^{1}H NMR (400 MHz, $CDCl_3$) δ 8.86 (s, 1H), 7.91 (d, *J* = 8.3 Hz, 2H), 7.60 (d, *J* = 8.1 Hz, 1H), 7.51–7.47 (m, 1H), 7.33 (d, *J* = 8.1 Hz, 2H), 7.22 (d, *J* = 2.2 Hz, 1H), 7.06 (t, *J* = 7.8 Hz, 1H), 2.41 (s, 3H); ^{13}C NMR (101 MHz, $CDCl_3$) δ 144.87, 138.14, 135.77, 135.57, 130.10, 128.20, 128.02, 127.51, 122.67, 121.87, 109.60, 105.32, 21.64; HRMS(ESI): m/z calcd for $C_{15}H_{12}BrNNaO_2S(M+Na)^+$: 371.9664, found: 371.9661.

2-methyl-3-tosyl-1H-indole (3n)

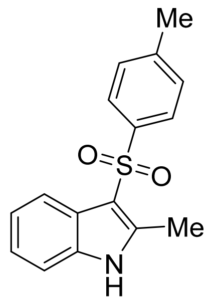

White solid, 125 mg, 88% yield, m.p. 179-181 °C; ^{1}H NMR (400 MHz, $CDCl_3$) δ 9.26 (s, 1H), 7.97 (d, *J* = 8.5 Hz, 1H), 7.83 (d, *J* = 8.3 Hz, 2H), 7.24–7.10 (m, 5H), 2.64 (s, 3H), 2.32 (s, 3H); ^{13}C NMR (101 MHz, $CDCl_3$) δ 143.38, 141.52, 141.19, 134.54, 129.75, 126.15, 125.40, 123.10, 122.17, 119.28, 111.42, 111.27, 21.57, 13.04; HRMS(ESI): m/z calcd for $C_{16}H_{16}NO_2S$ $(M+H)^+$: 286.0896, found: 286.0900.

2-(phenylsulfonyl)-1H-indole (3o)[1]

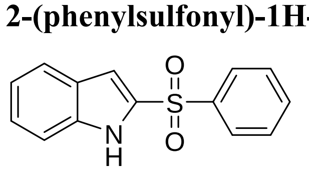

White solid, 104 mg, 81% yield, m.p. 159-161 °C; ^{1}H NMR (400 MHz, $CDCl_3$) δ 9.53 (s, 1H), 8.02 (d, *J* = 7.7 Hz, 2H), 7.65 (d, *J* = 8.0 Hz, 1H), 7.53 (t, *J* = 7.3 Hz, 1H), 7.45 (dd, *J* = 18.0, 8.4 Hz, 3H), 7.32–7.28 (m, 1H), 7.22 (s, 1H), 7.15 (t, *J* = 7.1 Hz, 1H); ^{13}C NMR (101 MHz, $CDCl_3$) δ 141.50, 137.51, 133.93, 133.56, 129.47, 127.32, 127.05, 126.14, 122.68, 121.62, 112.64, 109.39.

2-((4-fluorophenyl)sulfonyl)-1H-indole (3p)[1]

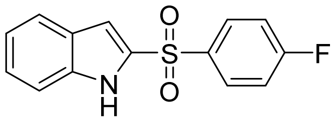

White solid, 88 mg, 65% yield, m.p. 139-141 °C; ^{1}H NMR (400 MHz, $CDCl_3$) δ 9.12 (s, 1H), 8.02 (dd, *J* = 8.9, 5.0 Hz, 2H), 7.67 (d, *J* = 8.0 Hz, 1H), 7.42 (d, *J* = 7.8 Hz, 1H), 7.33 (d, *J* = 8.3 Hz, 1H), 7.20–7.15 (m, 4H); ^{13}C NMR (101 MHz, $CDCl_3$) δ 137.29, 133.98, 130.34, 130.24, 127.21, 126.39, 122.87, 121.88, 116.95, 116.72, 112.45, 109.48.

2-((4-chlorophenyl)sulfonyl)-1H-indole (3q)[3]

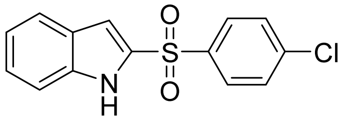

White solid, 104 mg, 71% yield, m.p. 146-148 °C; ^{1}H NMR (400 MHz, $CDCl_3$) δ 9.51 (s, 1H), 8.05 (d, *J* = 8.7 Hz, 2H), 7.50 (d, *J* = 8.8 Hz, 3H), 7.44–7.37 (m, 2H), 7.29– 7.23 (m, 2H); ^{13}C NMR (101 MHz, $CDCl_3$) δ 140.67, 138.65, 135.98, 133.34, 131.34, 129.58, 129.38, 127.39, 123.24, 122.52, 112.51.

2-((4-bromophenyl)sulfonyl)-1H-indole (3r)[1]

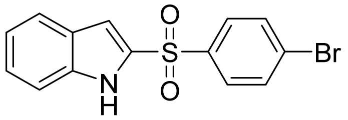

White solid, 97 mg, 58% yield, m.p. 191-193 °C; ^{1}H NMR (400 MHz, $CDCl_3$) δ 8.97 (s, 1H), 7.85 (d, *J* = 8.7 Hz, 2H), 7.67 (d, *J* = 8.1 Hz, 1H), 7.63 (d, *J* = 8.7 Hz, 2H), 7.42 (d, *J* = 8.4 Hz, 1H), 7.38–7.33 (m, 1H), 7.21–7.17 (m, 2H); ^{13}C NMR (101 MHz, $CDCl_3$) δ 140.67, 137.31, 133.63, 132.82, 131.77, 128.93, 127.25, 126.51, 122.92, 121.95, 112.42, 109.75.

2-((4-(trifluoromethyl)phenyl)sulfonyl)-1H-indole (3s)[1]

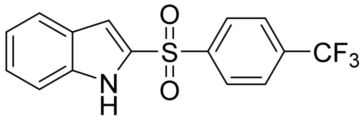

White solid, 109 mg, 67% yield, m.p. 151-153 °C; ^{1}H NMR (400 MHz, $CDCl_3$) δ 8.96 (s, 1H), 8.12 (d, *J* = 8.2 Hz, 2H), 7.76 (d, *J* = 8.3 Hz, 2H), 7.68 (d, *J* = 8.1 Hz, 1H), 7.43 (d, *J* = 8.4 Hz, 1H), 7.39–7.35 (m, 1H), 7.24–7.08 (m, 2H); ^{13}C NMR (101 MHz, $CDCl_3$) δ 145.05, 137.35, 132.82,127.81, 127.11, 126.61, 122.86, 121.94, 112.33, 110.25, 65.61.

2-((4-nitrophenyl)sulfonyl)-1H-indole (3t)

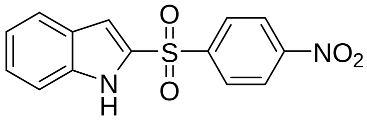

Light yellow solid, 102 mg, 68% yield, m.p. 129-132 °C; ^{1}H NMR (400 MHz, CDCl$_3$) δ 8.90 (s, 1H), 8.36–8.34 (m, 1H), 8.33 (d, *J* = 2.1 Hz, 1H), 8.20–8.15 (m, 2H), 7.69 (d, *J* = 8.1 Hz, 1H), 7.46–7.42 (m, 1H), 7.41–7.37 (m, 1H), 7.29 (dd, *J* = 2.1, 0.8 Hz, 1H), 7.22 (ddd, *J* = 8.0, 6.8, 1.1 Hz, 1H); ^{13}C NMR (101 MHz, CDCl$_3$) δ 150.40, 147.18, 137.49, 132.20, 129.24, 128.58, 127.15, 126.92, 124.61, 124.46, 122.97, 122.12, 112.35, 110.85; HRMS(ESI): m/z calcd for $C_{14}H_{10}N_2NaO_4S(M+Na)^+$: 325.0253, found: 325.0251.

2-((4-(tert-butyl)phenyl)sulfonyl)-1H-indole (3u)[1]

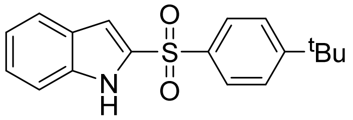

White solid, 118 mg, 76% yield, m.p. 192-194 °C; ^{1}H NMR (400 MHz, CDCl$_3$) δ 9.50 (s, 1H), 7.94 (d, *J* = 8.7 Hz, 2H), 7.65 (d, *J* = 8.1 Hz, 1H), 7.47 (d, *J* = 8.7 Hz, 2H), 7.42 (d, *J* = 7.7 Hz, 1H), 7.29 (ddd, *J* = 8.3, 7.1, 1.0 Hz, 1H), 7.21 (d, *J* = 1.3 Hz, 1H), 7.18 – 7.12 (m, 1H), 1.27 (s, 9H); ^{13}C NMR (101 MHz, CDCl$_3$) δ 157.57, 138.47, 137.38, 134.50, 127.28, 127.12, 126.54, 126.02, 122.67, 121.58, 112.59, 109.01, 35.33, 31.10.

2-((4-methoxyphenyl)sulfonyl)-1H-indole (3v)[1]

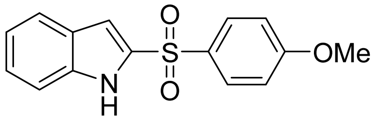

White solid, 73 mg, 51% yield, m.p. 186-187 °C; ^{1}H NMR (400 MHz, CDCl$_3$) δ 8.25 (s, 1H), 7.55 (d, *J* = 8.0 Hz, 1H), 7.37 (d, *J* = 2.6 Hz, 1H), 7.32 (d, *J* = 8.1 Hz, 1H), 7.19 – 7.14 (m, 1H), 7.07 (ddd, *J* = 12.3, 7.3, 1.6 Hz, 3H), 6.69–6.62 (m, 2H), 3.65 (s, 3H); ^{13}C NMR (101 MHz, CDCl$_3$)δ 157.89, 136.57, 130.14, 129.62, 129.12, 128.68, 123.06, 120.89, 119.77, 114.61, 111.64, 104.72, 55.46.

2-(naphthalen-2-ylsulfonyl)-1H-indole (3w)[1]

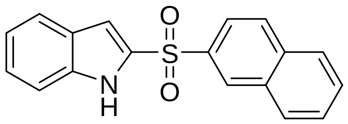

White solid, 124 mg, 81% yield, m.p. 156-158 °C; ^{1}H NMR (400 MHz, $CDCl_3$) δ 8.91 (s, 1H), 8.61 (s, 1H), 7.99–7.91 (m, 3H), 7.87 (d, *J* = 7.7 Hz, 1H), 7.68–7.58 (m, 3H), 7.49–7.29 (m, 3H), 7.17 (t, *J* = 7.5 Hz, 1H); ^{13}C NMR (101 MHz, $CDCl_3$) δ 138.24, 137.07, 135.14, 134.19, 132.18, 129.77, 129.44, 129.30, 128.74, 127.96, 127.74, 127.16, 126.11, 122.73, 122.27, 121.63, 112.22, 109.32.

1-methyl-2-(phenylsulfonyl)-1H-pyrrole (5a)[4]

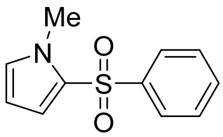

Light yellow viscous solid, 62 mg, 57% yield, m.p. 76-78 °C; ^{1}H NMR (400 MHz, $CDCl_3$) δ 7.88 (d, *J* = 7.1 Hz, 2H), 7.57–7.48 (m, 3H), 7.03 (dd, *J* = 4.0, 1.9 Hz, 1H), 6.76 (s, 1H), 6.17 (dd, *J* = 4.0, 2.6 Hz, 1H), 3.70 (s, 3H); ^{13}C NMR (101 MHz, $CDCl_3$) δ 142.25, 132.98, 129.81, 129.31, 127.23, 118.96, 108.45, 35.73.

1-methyl-2-tosyl-1H-pyrrole (5b)[4]

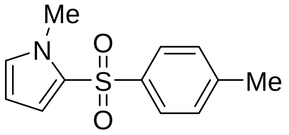

White solid, 55 mg, 47% yield, m.p. 99-101 °C; ^{1}H NMR (400 MHz, $CDCl_3$) δ 7.77 (d, *J* = 8.3 Hz, 2H), 7.29 (d, *J* = 8.0 Hz, 2H), 7.00 (dd, *J* = 4.0, 1.9 Hz, 1H), 6.74 (t, *J* = 2.2 Hz, 1H), 6.16 (dd, *J* = 4.0, 2.6 Hz, 1H), 3.70 (s, 3H), 2.41 (s, 3H); ^{13}C NMR (101 MHz, $CDCl_3$) δ 143.90, 139.36, 129.94, 129.54, 128.44, 118.63, 108.35, 35.72, 21.68.

2-tosyl-1H-pyrrole (5b′)[5]

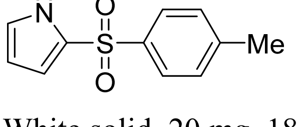

White solid, 20 mg, 18% yield. ^{1}H NMR (400 MHz, $CDCl_3$): δ 9.35 (s, 1H), 7.80 (d, *J* = 8.4 Hz, 2H), 7.28 (d, *J* = 8.0 Hz, 2H), 6.95 (td, *J* = 2.8, 1.6 Hz, 1H), 6.85 (ddd, *J* = 4.0, 2.4, 1.6 Hz, 1H), 6.28 (dt, *J* = 3.6, 2.4 Hz, 1H), 2.39 (s, 3H).

2-((4-(tert-butyl)phenyl)sulfonyl)-1-methyl-1H-pyrrole (5c)

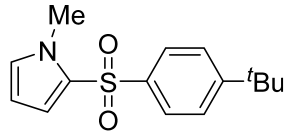

Pale yellow solid, 58 mg, 42% yield, m.p. 59-61 °C; ^{1}H NMR (400 MHz, $CDCl_3$) δ 7.80 (d, *J* = 8.7 Hz, 2H), 7.50 (d, *J* = 8.7 Hz, 2H), 7.02 (dd, *J* = 4.0, 1.9 Hz, 1H), 6.75 (t, *J* = 2.2 Hz, 1H), 6.16 (dd, *J* = 4.0, 2.6 Hz, 1H), 3.72 (s, 3H), 1.32 (s, 9H); ^{13}C NMR (101 MHz, $CDCl_3$) δ 156.84, 139.25, 129.56, 128.43, 127.16, 126.33, 118.64, 108.33, 35.79, 35.30, 31.18; HRMS(ESI): m/z calcd for $C_{15}H_{20}NO_2S(M+H)^+$: 278.1209, found: 278.1208.

2-((4-fluorophenyl)sulfonyl)-1-methyl-1H-pyrrole (5d)[4]

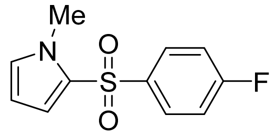

Pale yellow solid, 59 mg, 50% yield, m.p. 61-63 °C; ^{1}H NMR (400 MHz, $CDCl_3$) δ 7.90 (dd, *J* = 8.9, 5.1 Hz, 2H), 7.17 (t, *J* = 8.6 Hz, 2H), 7.01 (dd, *J* = 4.0, 1.9 Hz, 1H), 6.78 (t, *J* = 2.1 Hz, 1H), 6.17 (dd, *J* = 4.0, 2.6 Hz, 1H), 3.71 (s, 3H); ^{13}C NMR (101 MHz, $CDCl_3$) δ 166.49, 163.95, 138.35, 130.07, 129.97, 127.72, 118.97, 116.67, 116.44, 108.52, 35.69.

2-((4-chlorophenyl)sulfonyl)-1-methyl-1H-pyrrole (5e)[4]

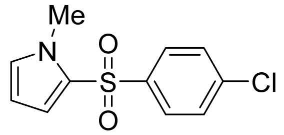

Yellow solid, 66 mg, 52% yield, m.p. 73-75 °C; ^{1}H NMR (400 MHz, $CDCl_3$) δ 7.82 (d, *J* = 8.8 Hz, 2H), 7.47 (d, *J* = 8.8 Hz, 2H), 7.03 (dd, *J* = 4.1, 1.9 Hz, 1H), 6.78 (t, *J* = 2.2 Hz, 1H), 6.18 (dd, *J* = 4.1, 2.6 Hz, 1H), 3.71 (s, 3H); ^{13}C NMR (101 MHz, $CDCl_3$) δ 140.87, 139.57, 130.13, 129.65, 128.77, 127.54, 119.30, 108.70, 35.80.

2-((4-bromophenyl)sulfonyl)-1-methyl-1H-pyrrole (5f)[4]

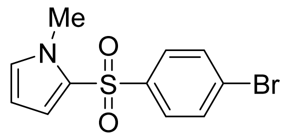

Pale yellow solid, 70 mg, 47% yield, m.p. 90-92 °C; ^{1}H NMR (400 MHz, $CDCl_3$) δ 7.74 (d, *J* = 8.7 Hz, 2H), 7.64 (d, *J* = 8.7 Hz, 2H), 7.03 (dd, *J* = 4.1, 1.9 Hz, 1H), 6.78 (t, *J* = 2.2 Hz, 1H), 6.18 (dd, *J* = 4.1, 2.6 Hz, 1H), 3.71 (s, 3H); ^{13}C NMR (101 MHz, $CDCl_3$) δ 141.39, 132.62, 130.16, 128.83, 128.07, 127.45, 119.32, 35.79.

1-methyl-2-((4-(trifluoromethyl)phenyl)sulfonyl)-1H-pyrrole (5g)

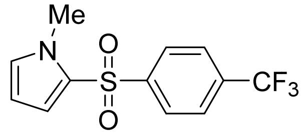

Pale yellow solid, 62 mg, 43% yield, m.p. 66-68 °C; ^{1}H NMR (400 MHz, $CDCl_3$) δ 8.01 (d, *J* = 8.2 Hz, 2H), 7.77 (d, *J* = 8.3 Hz, 2H), 7.09 (dd, *J* = 4.1, 1.9 Hz, 1H), 6.81 (t, *J* = 2.2 Hz, 1H), 6.21 (dd, *J* = 4.1, 2.6 Hz, 1H), 3.73 (s, 3H); ^{13}C NMR (101 MHz, $CDCl_3$) δ 145.96, 134.76, 134.43, 130.61, 127.77, 126.85, 126.53, 126.50, 124.63, 121.92, 119.92, 108.94, 35.87; HRMS(ESI): m/z calcd for $C_{12}H_{11}F_3NO_2S$ $(M+H)^+$: 290.0457, found: 290.0457.

2-((3-bromophenyl)sulfonyl)-1-methyl-1H-pyrrole (5h)

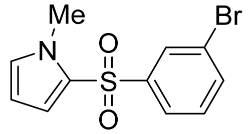

Pale yellow solid, 79 mg, 53% yield, m.p. 55-58 °C; ^{1}H NMR (400 MHz, $CDCl_3$) δ 8.01 (t, *J* = 1.8 Hz, 1H), 7.81 (d, *J* = 7.9 Hz, 1H), 7.68 (d, *J* = 8.0 Hz, 1H), 7.38 (t, *J* = 7.9 Hz, 1H), 7.05 (dd, *J* = 4.1, 1.9 Hz, 1H), 6.80 (t, *J* = 2.2 Hz, 1H), 6.20 (dd, *J* = 4.1, 2.6 Hz, 1H), 3.72 (s, 3H); ^{13}C NMR (101 MHz, $CDCl_3$) δ 144.26, 136.02, 130.86, 130.36, 130.09, 127.11, 125.78, 123.25, 119.62, 108.77, 35.85; HRMS(ESI): m/z calcd for $C_{11}H_{11}BrNO_2S(M)^+$: 299.9688, found: 298.9690.

1-methyl-2-(naphthalen-2-ylsulfonyl)-1H-pyrrole (5i)

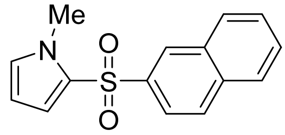

Pale yellow solid, 60 mg, 45% yield, m.p. 60-63 °C; ^{1}H NMR (400 MHz, $CDCl_3$) δ 8.50 (s, 1H), 7.93 (td, *J* = 17.0, 16.6, 7.8 Hz, 3H), 7.80 (dd, *J* = 8.7, 1.9 Hz, 1H), 7.66–7.57 (m, 2H), 7.10 (dd, *J* = 4.0, 1.9 Hz, 1H), 6.75 (t, *J* = 2.2 Hz, 1H), 6.19 (dd, *J*

= 4.0, 2.6 Hz, 1H), 3.72 (s, 3H), ^{13}C NMR (101 MHz, $CDCl_3$) δ 139.13, 135.02, 132.30, 129.85, 129.73, 129.47, 129.15, 128.33, 128.06, 127.72, 122.71, 119.14, 108.54, 35.81; HRMS(ESI): m/z calcd for $C_{15}H_{14}NO_2S(M+H)^+$: 272.0740, found: 272.0745.

References

1 F. Xiao, H. Chen, H. Xie, S. Chen, L. Yang, G.-J. Deng, *Org. Lett.* **2014**, *16*, 50.

2 Katrun, P.; Mueangkaew, C.; Pohmakotr, M.; Reutrakul, V.; Jaipetch, T.; Soorukram, D.; Kuhakarn, C. *J. Org. Chem*. **2014**, *79*, 1778.

3 Yang, Y.; Li, W. M.; Xia, C. C.; Ying, B. B.; Shen, C.; Zhang, P. F. *ChemCatChem*. **2016**, *8*, 304.

4 Santosh K. Pagire, Asik Hossain, and Oliver Reiser, *Org. Lett.* **2018**, *20*, 648.

5 Yadav, J. S., Reddy, B. V. S., Kondaji, G., Rao, R. S., Kumar, S. P. *Tetrahedron lett*., **2002**, *43*, 8133.

^{1}H and ^{13}C NMR spectra

2-tosyl-1H-indole (3a)

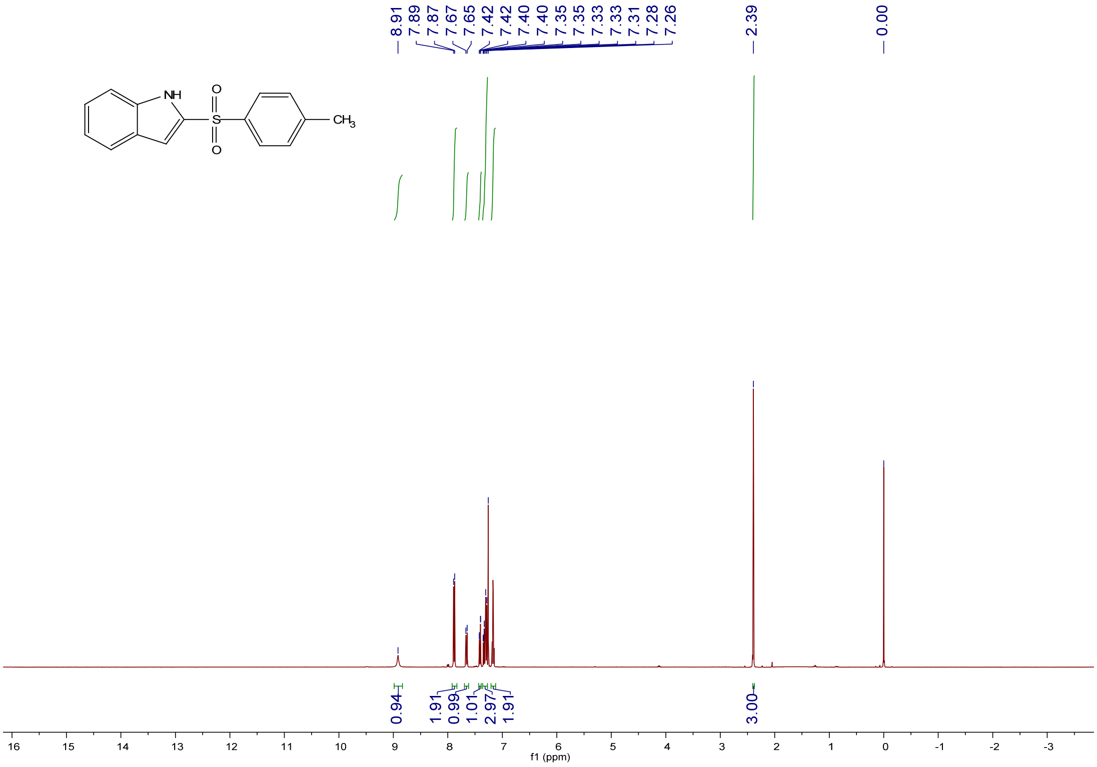

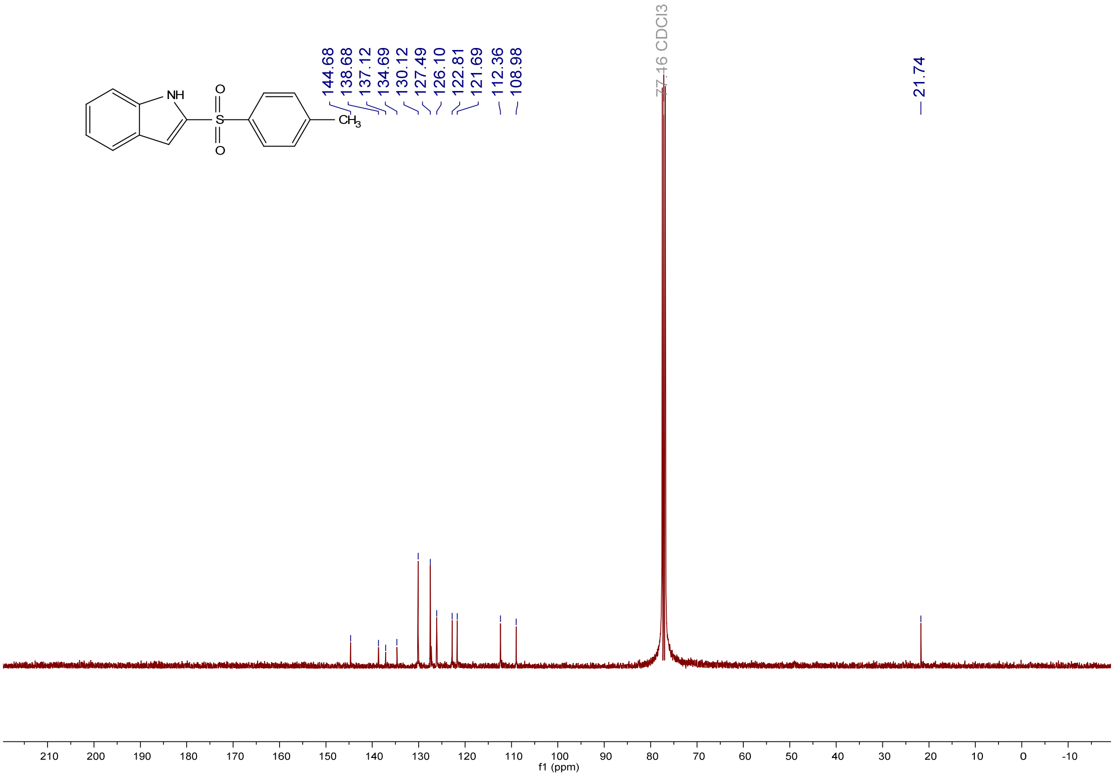

1-methyl-2-tosyl-1H-indole (3b)

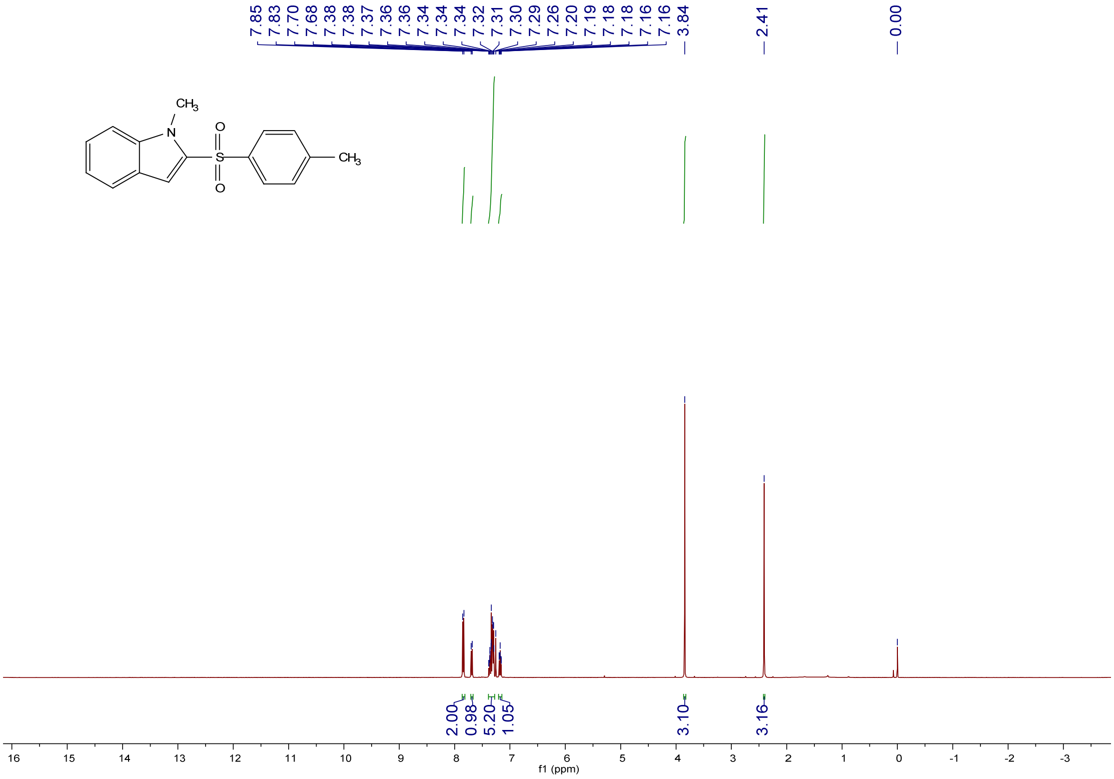

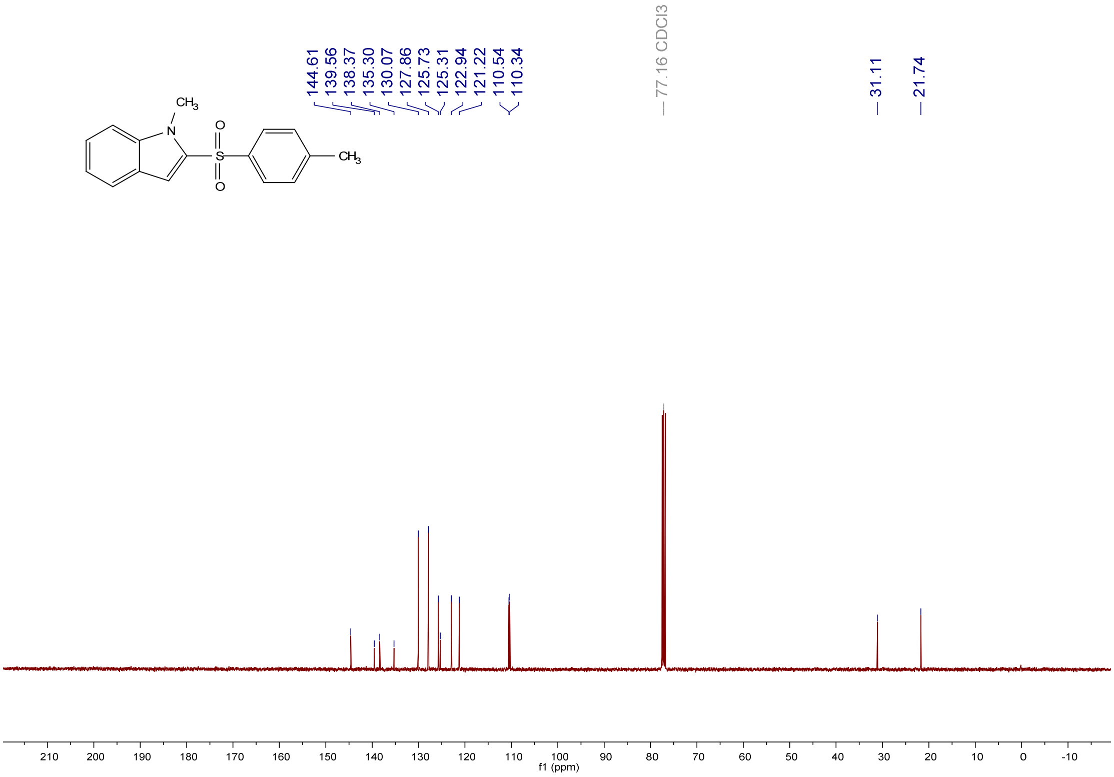

1-ethyl-2-tosyl-1H-indole (3c)

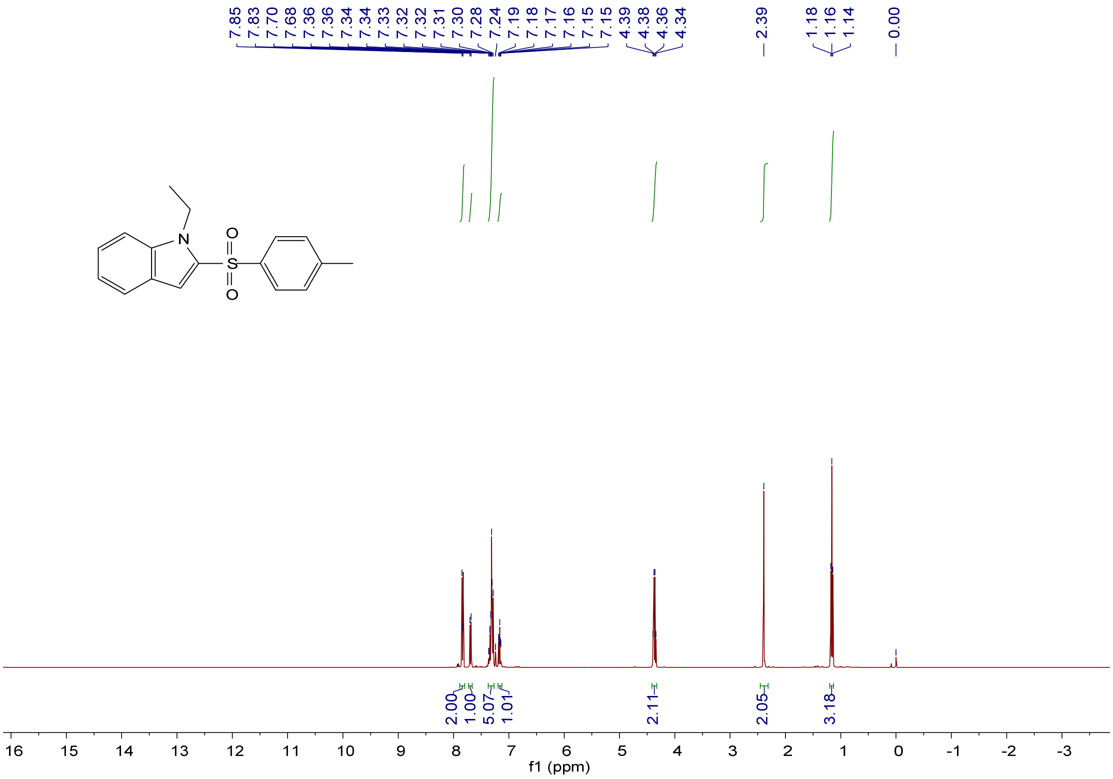

1-benzyl-2-tosyl-1H-indole (3d)

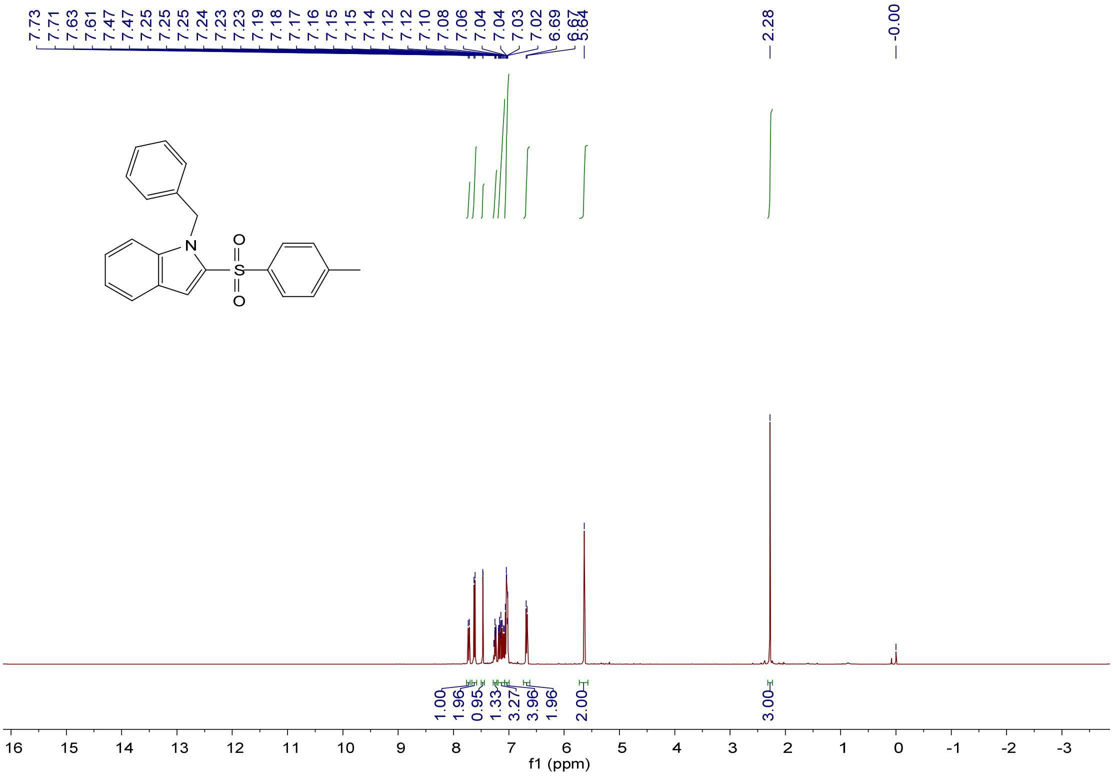

3-methyl-2-tosyl-1H-indole (3e)

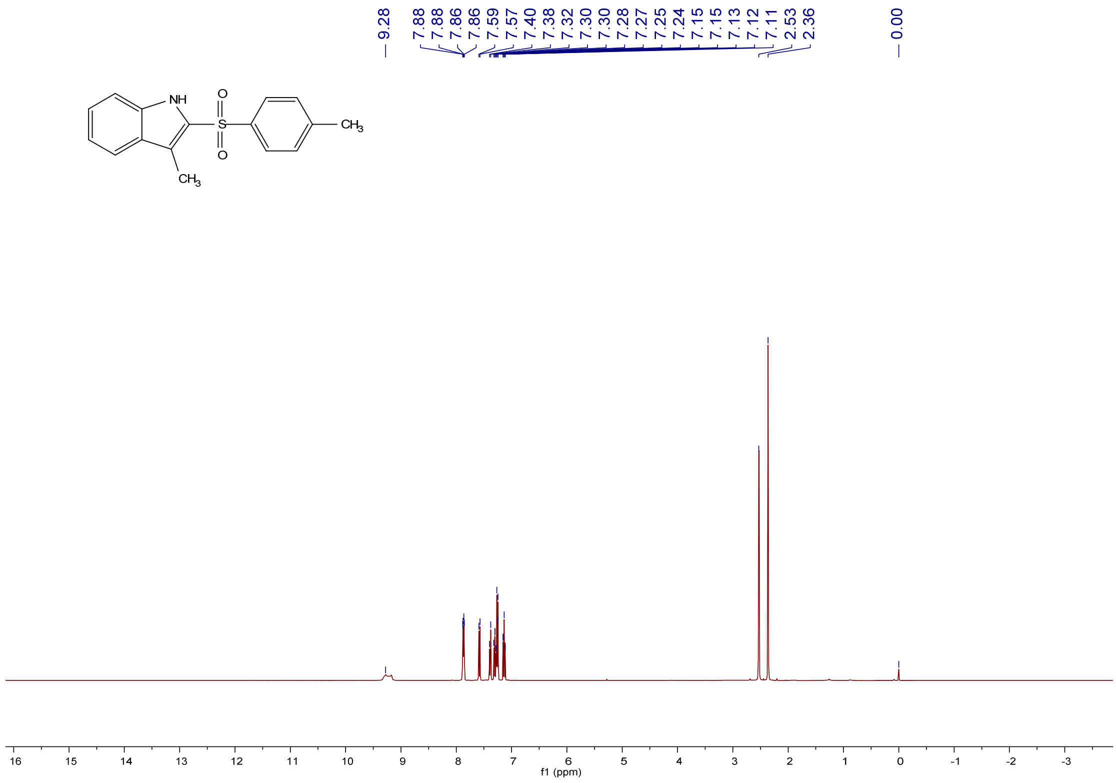

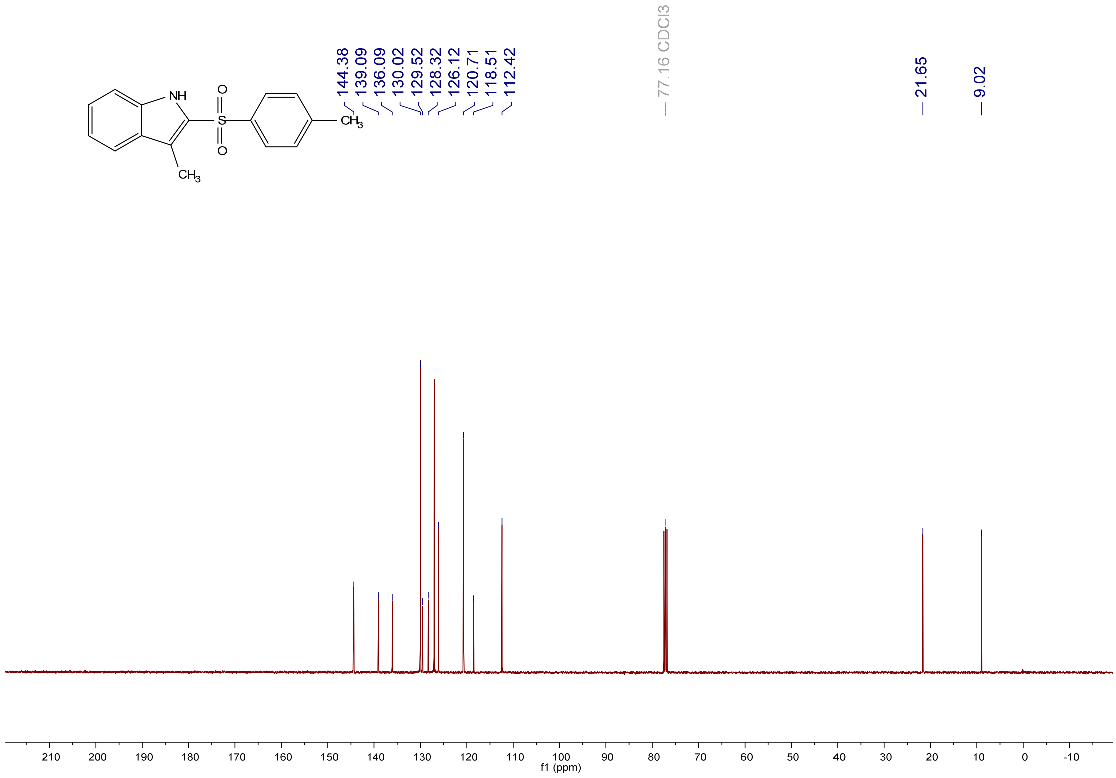

5-methyl-2-tosyl-1H-indole (3f)

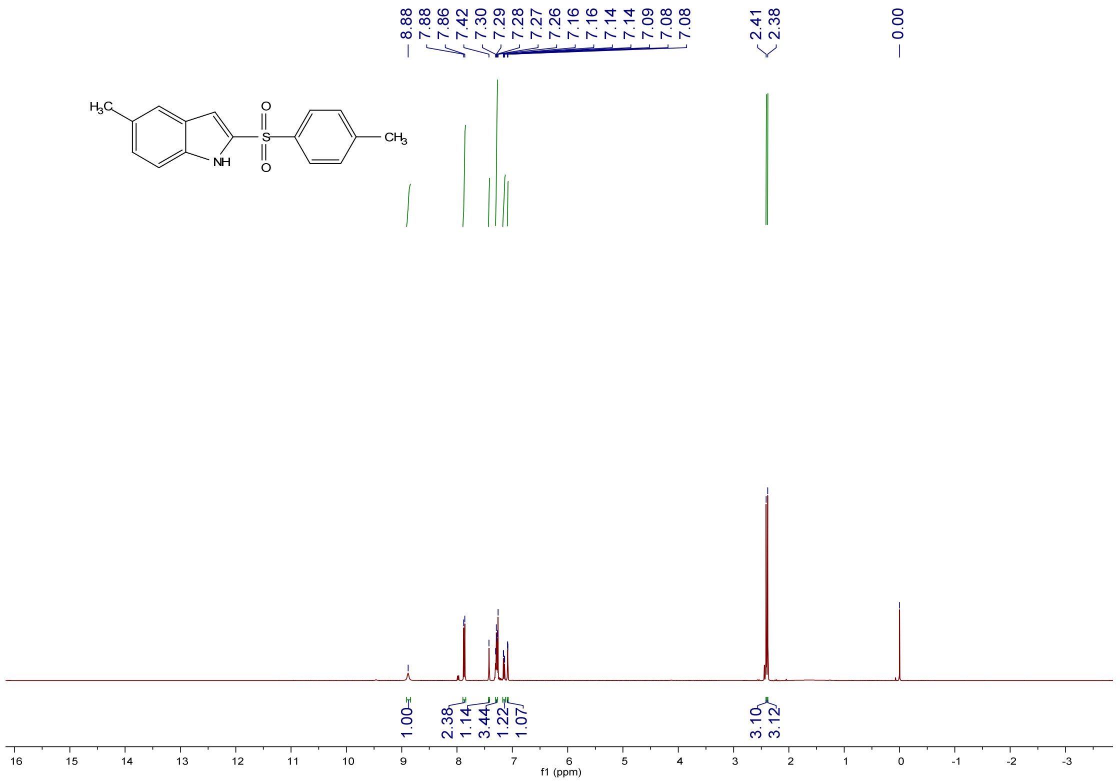

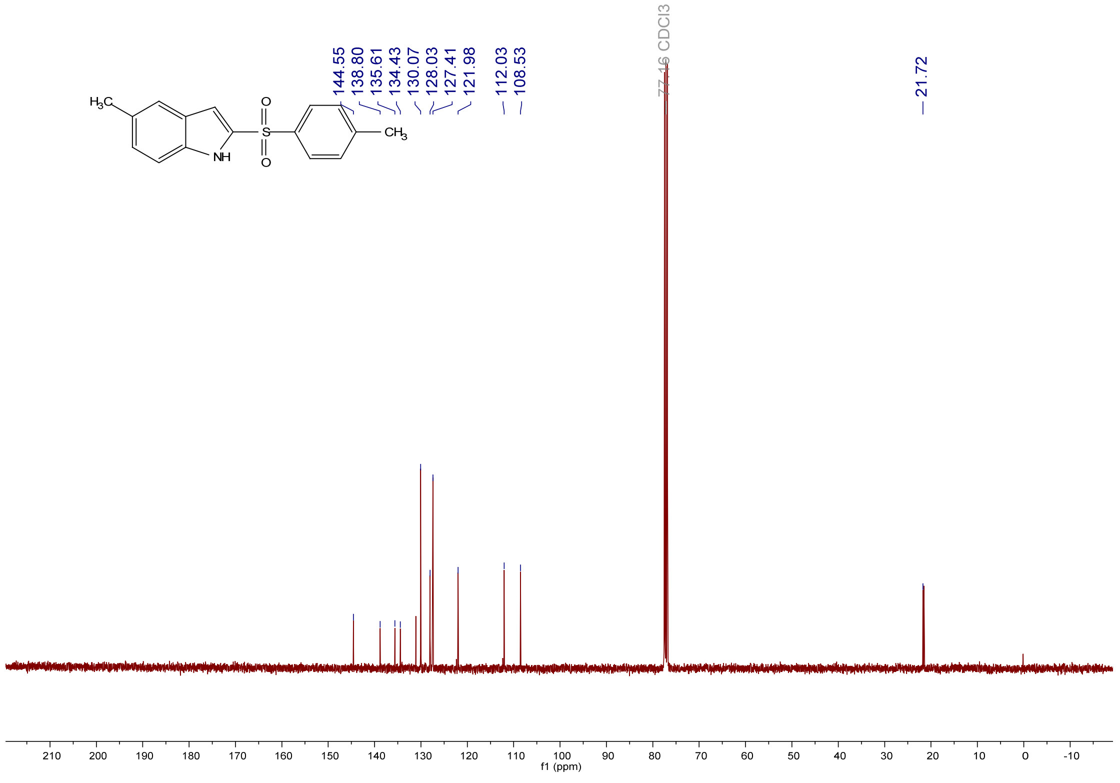

7-methyl-2-tosyl-1H-indole (3g)

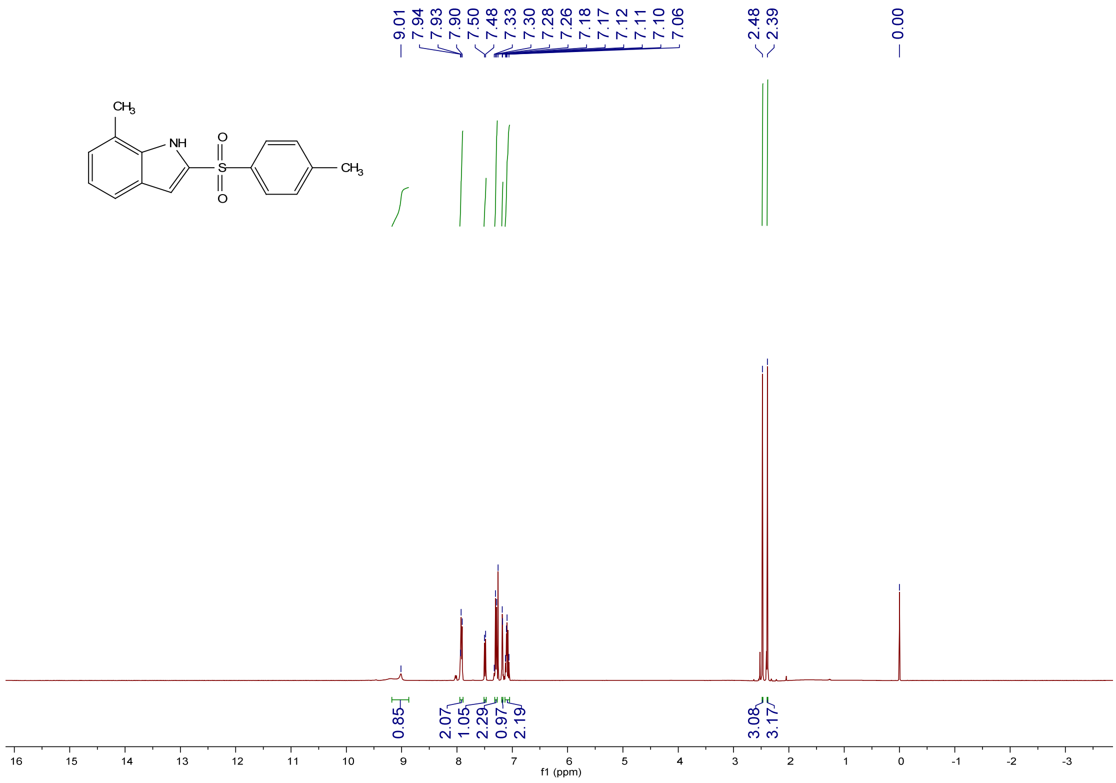

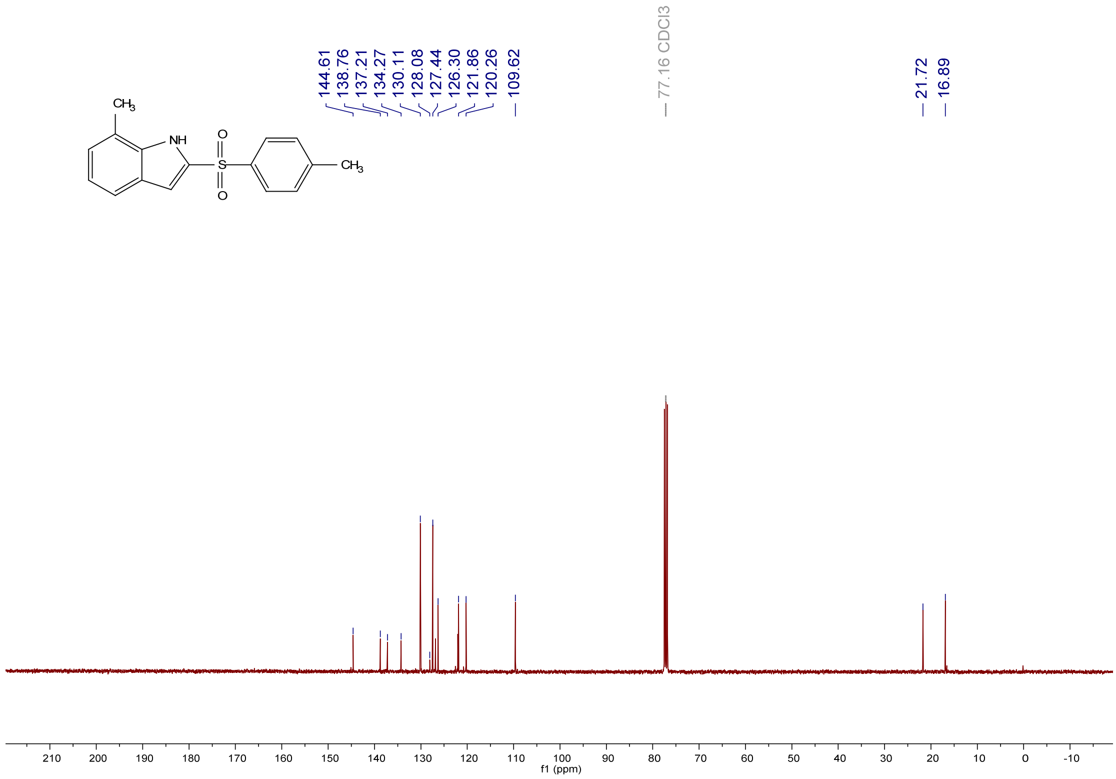

4-methoxy-2-tosyl-1H-indole (3h)

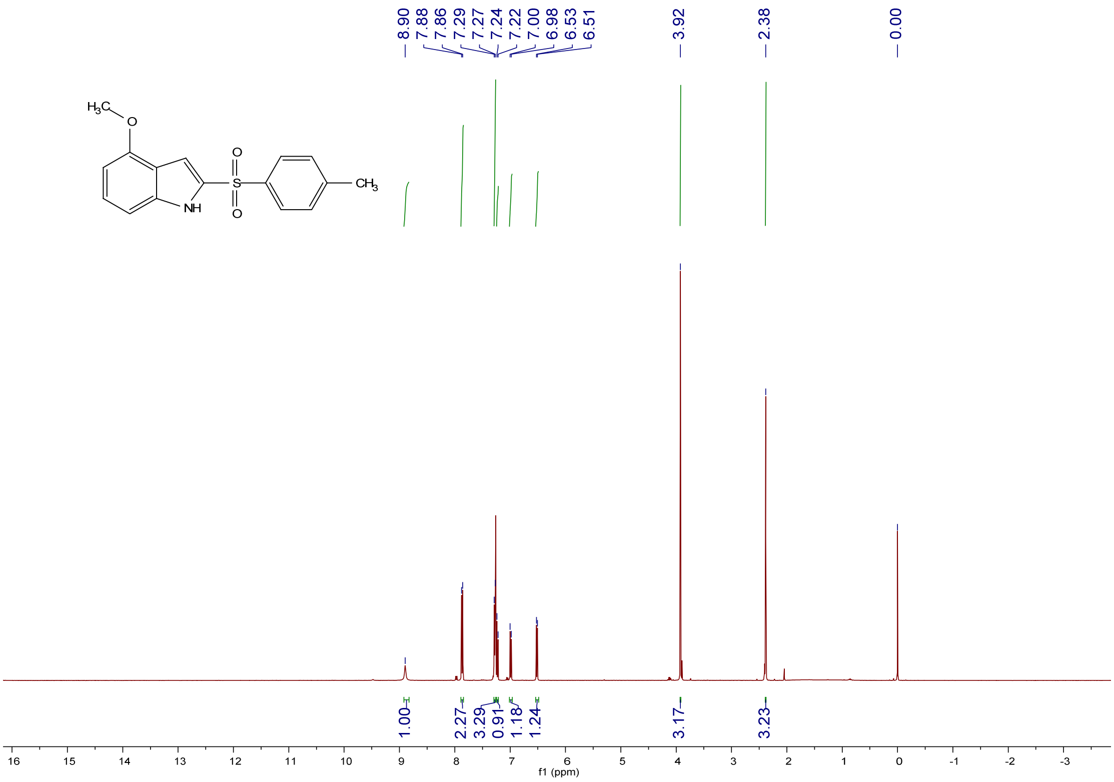

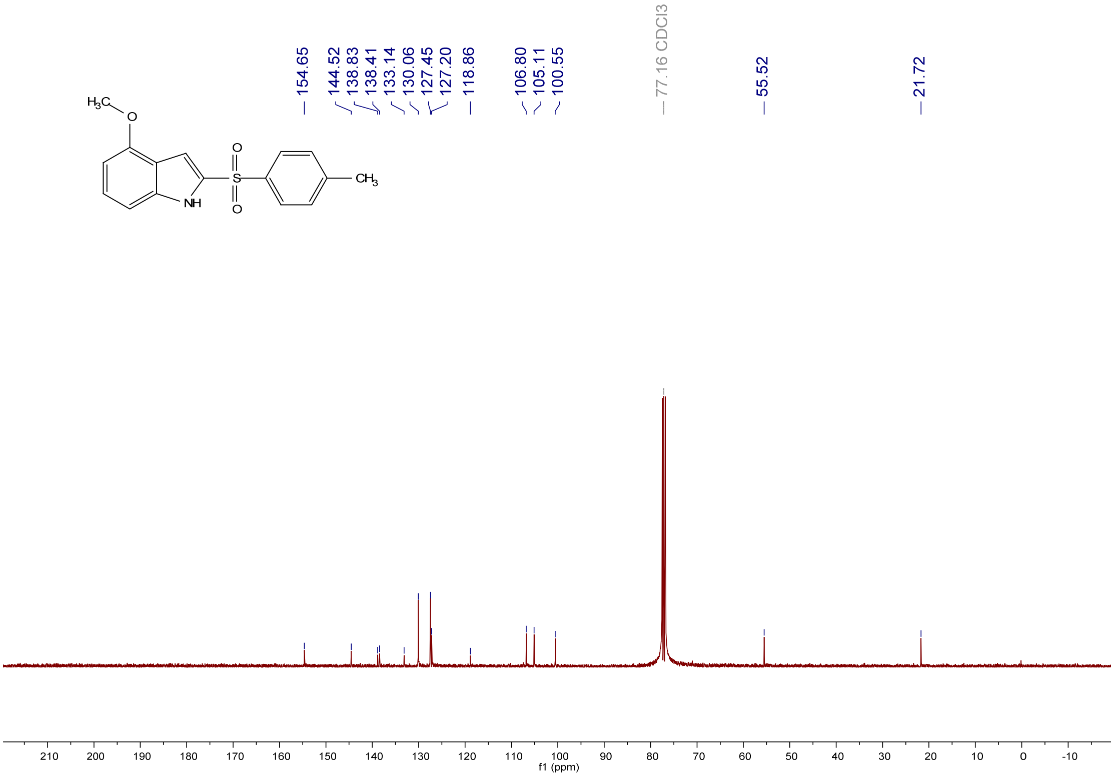

5-methoxy-2-tosyl-1H-indole (3i)

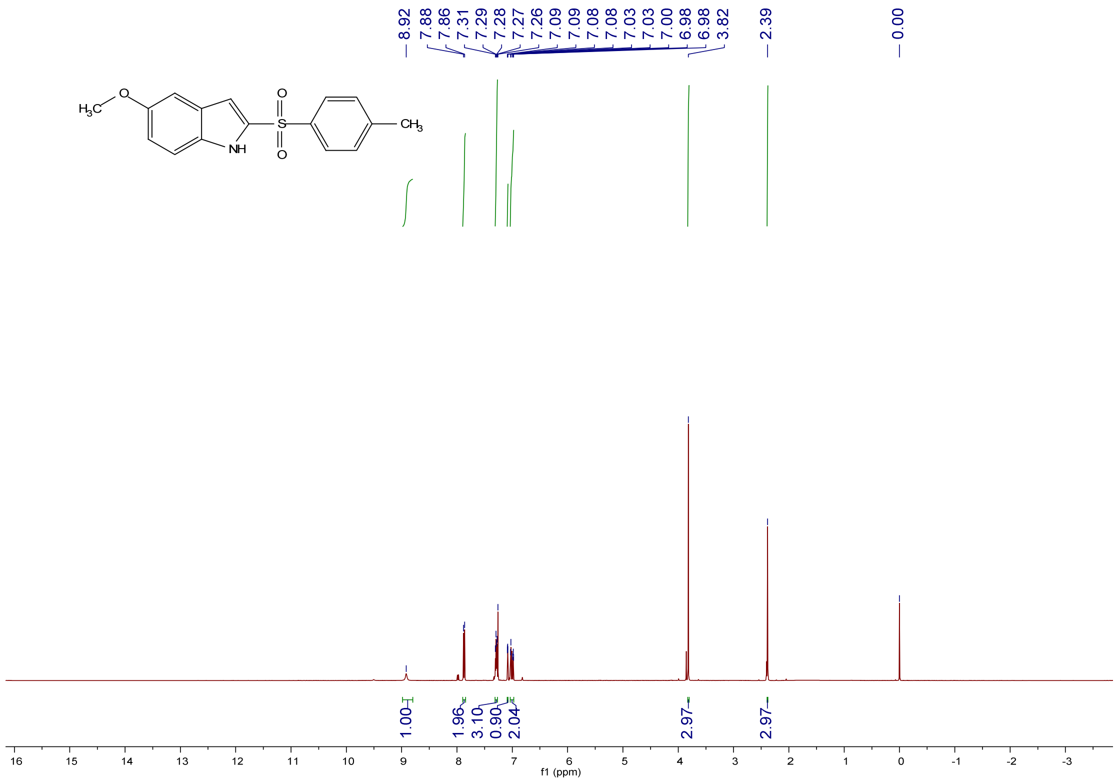

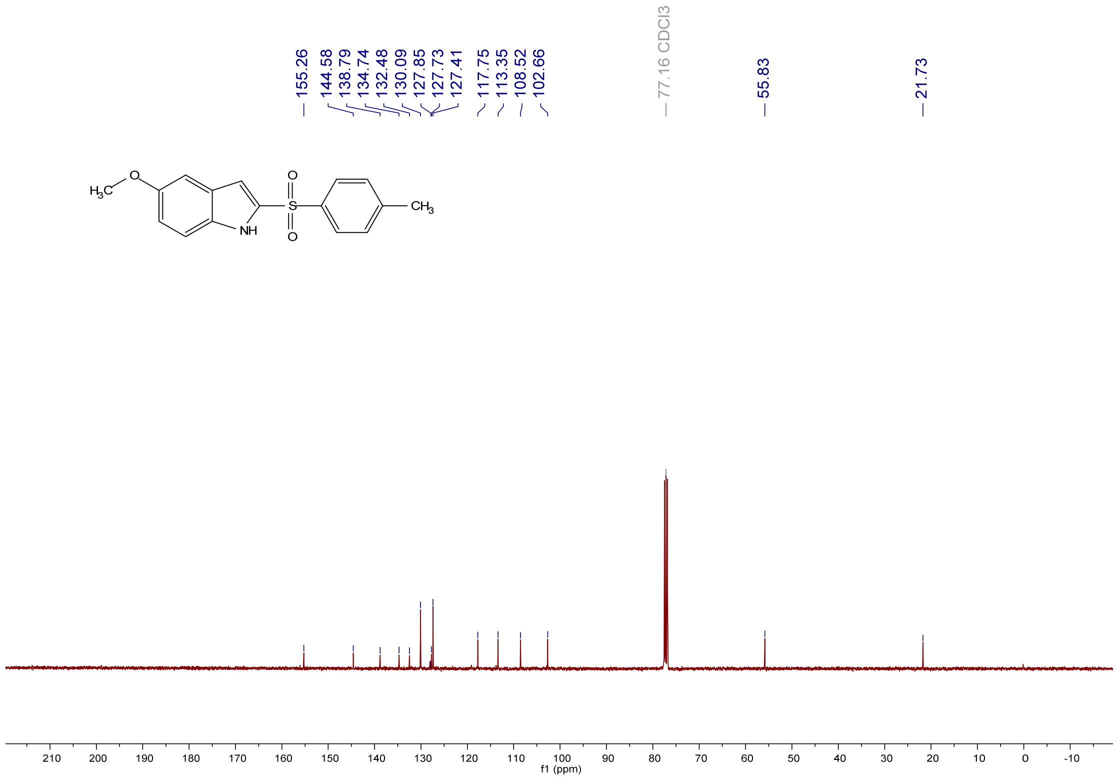

7-methoxy-2-tosyl-1H-indole (3j)

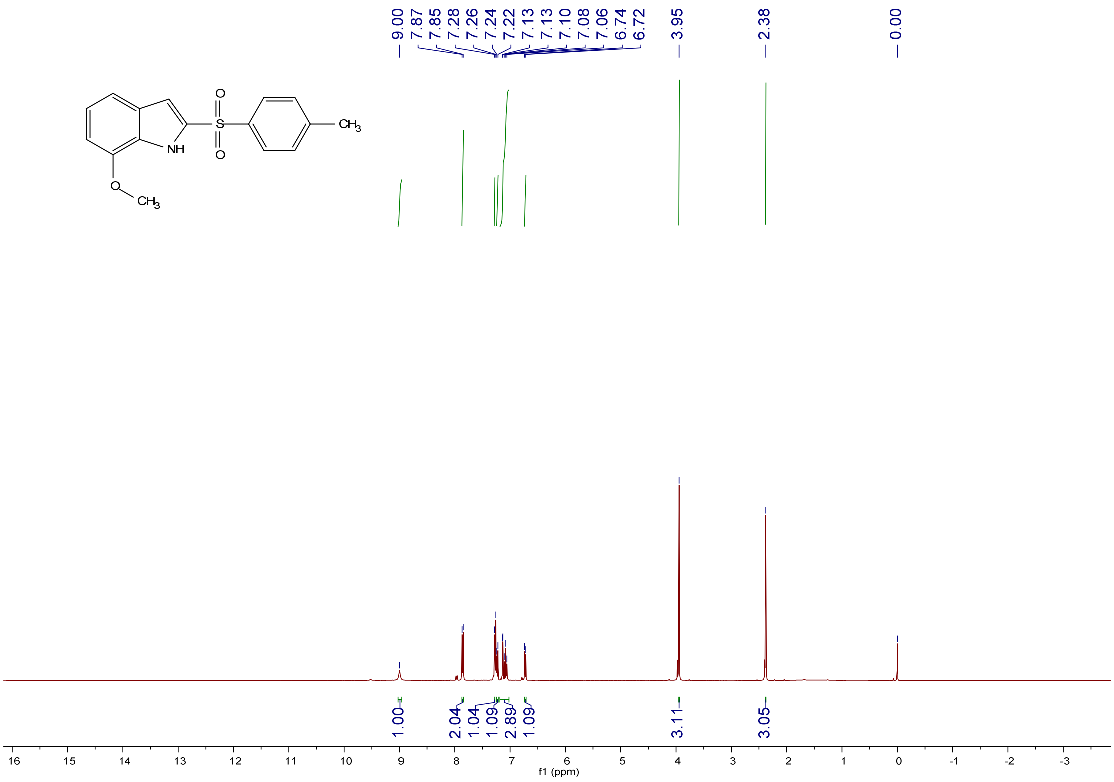

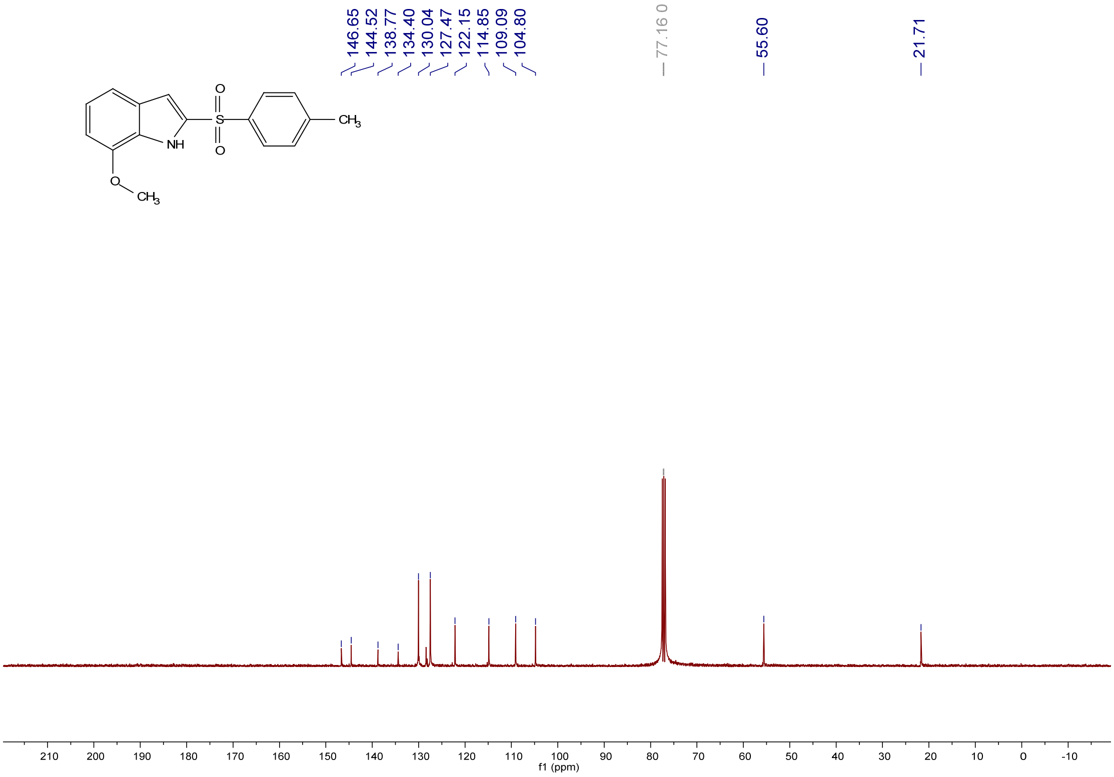

4-(benzyloxy)-2-tosyl-1H-indole (3k)

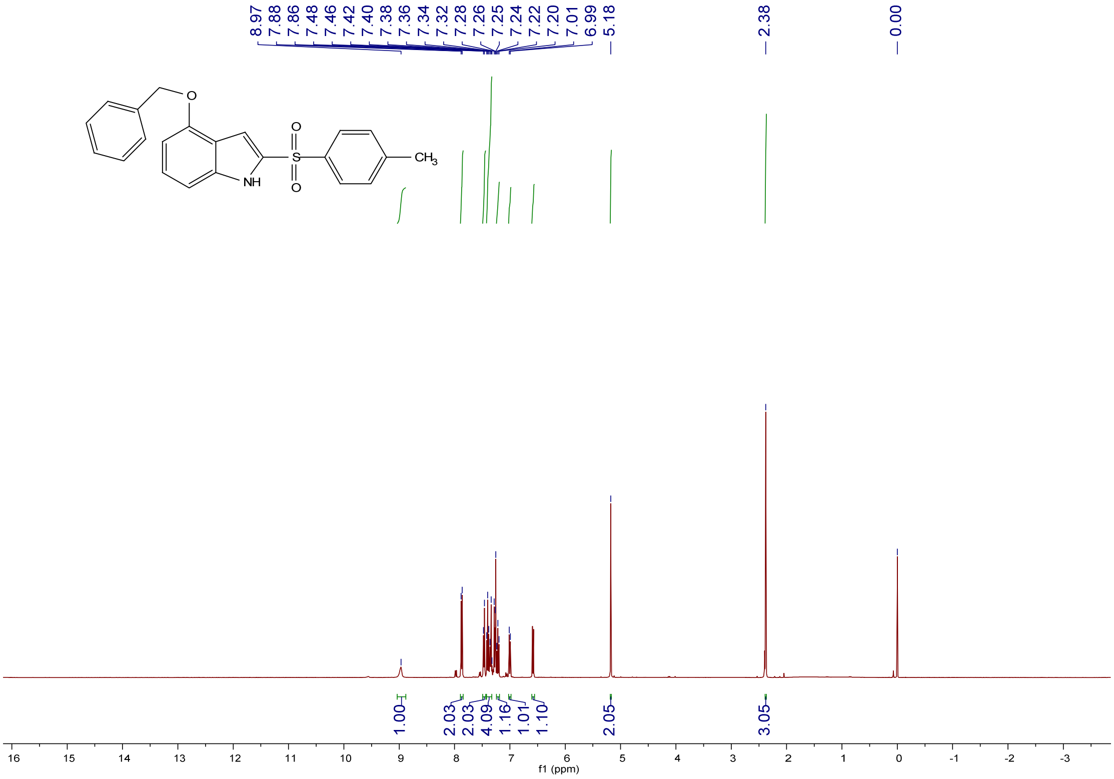

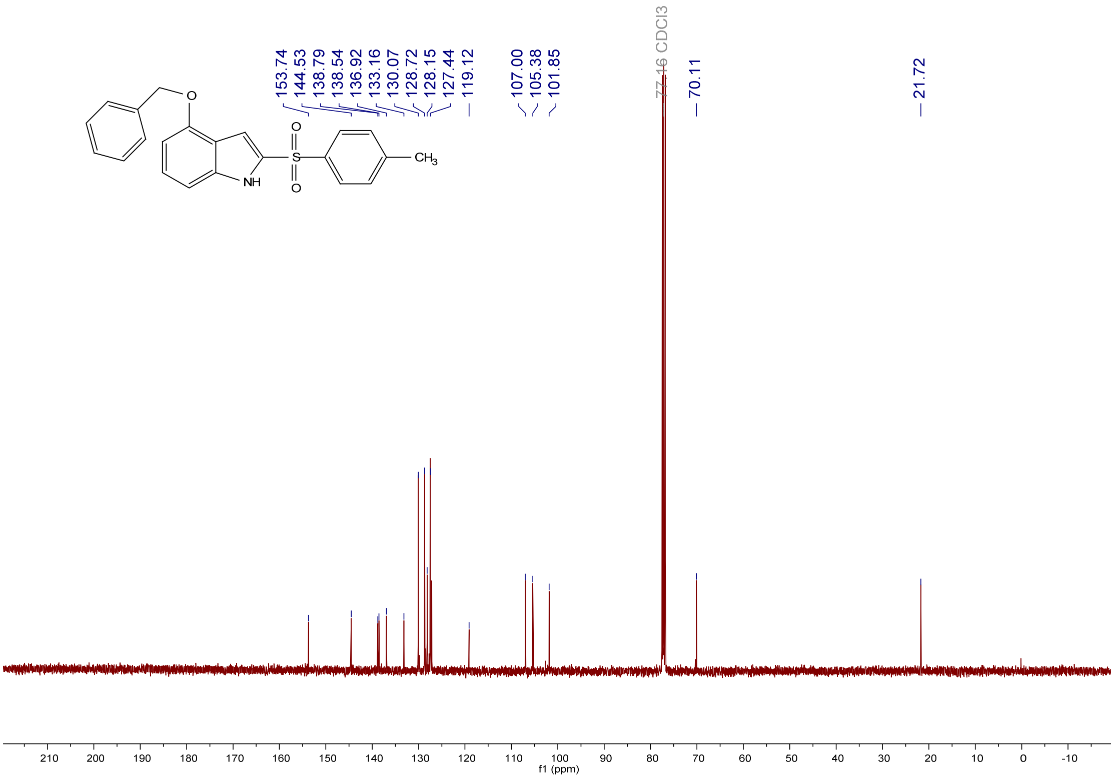

6-bromo-2-tosyl-1H-indole (3l)

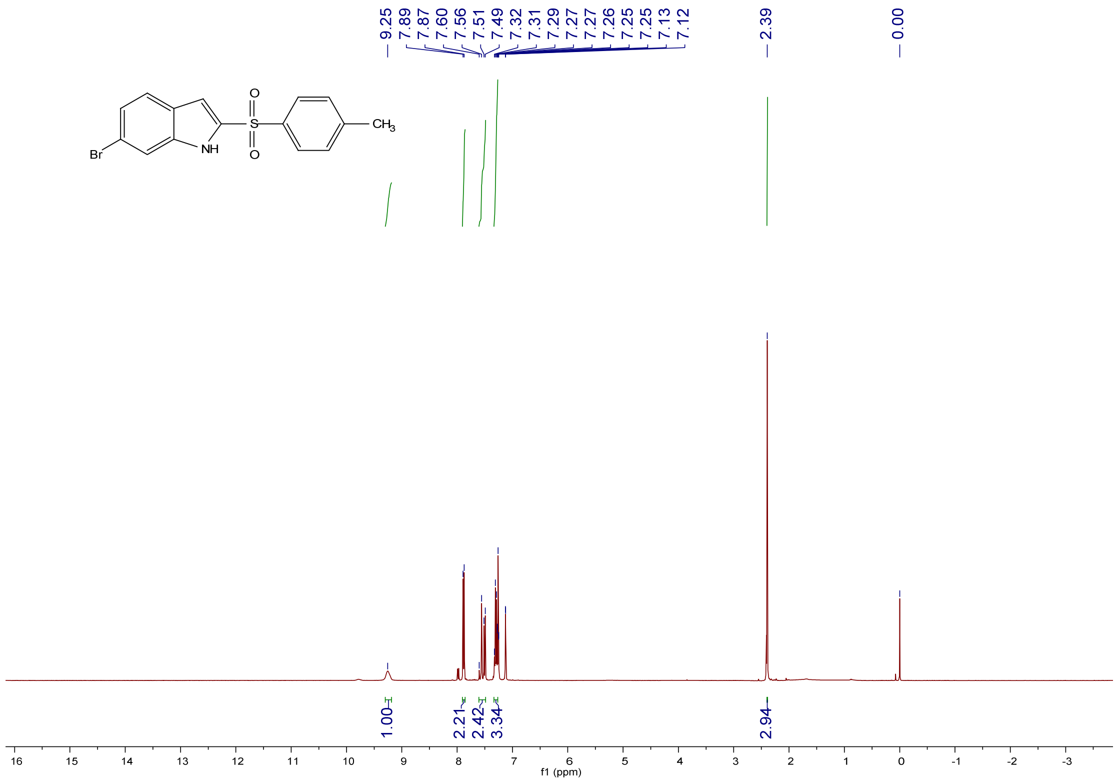

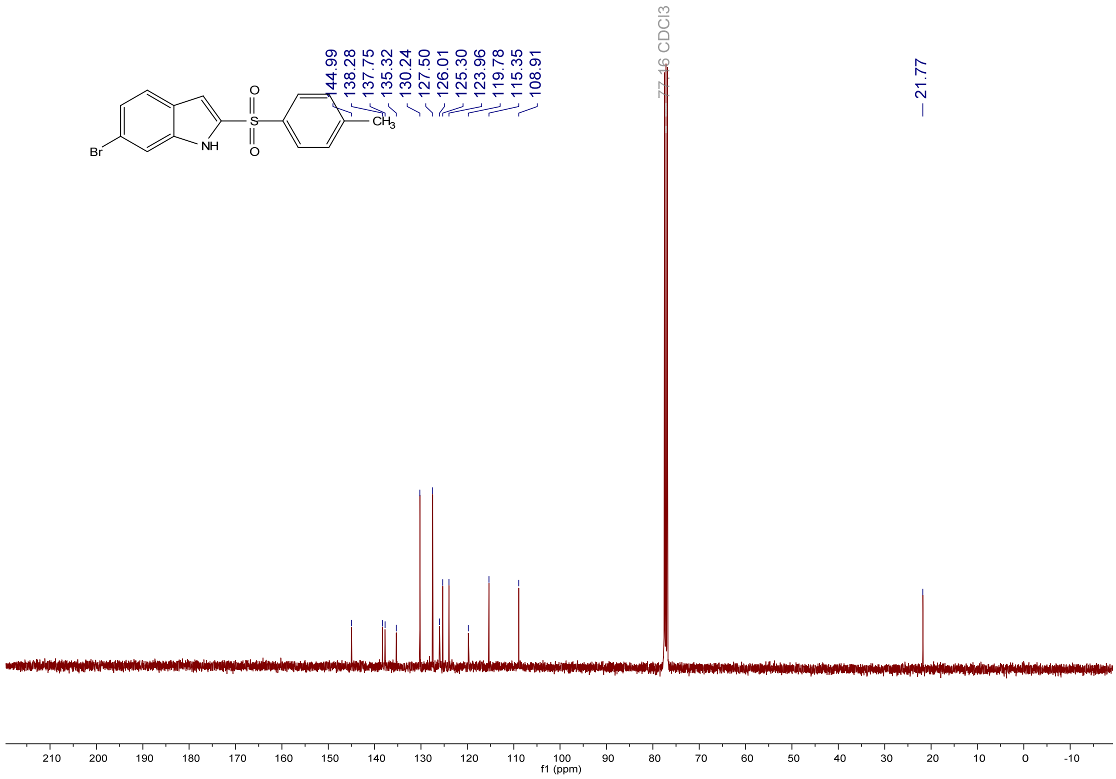

7-bromo-2-tosyl-1H-indole (3m)

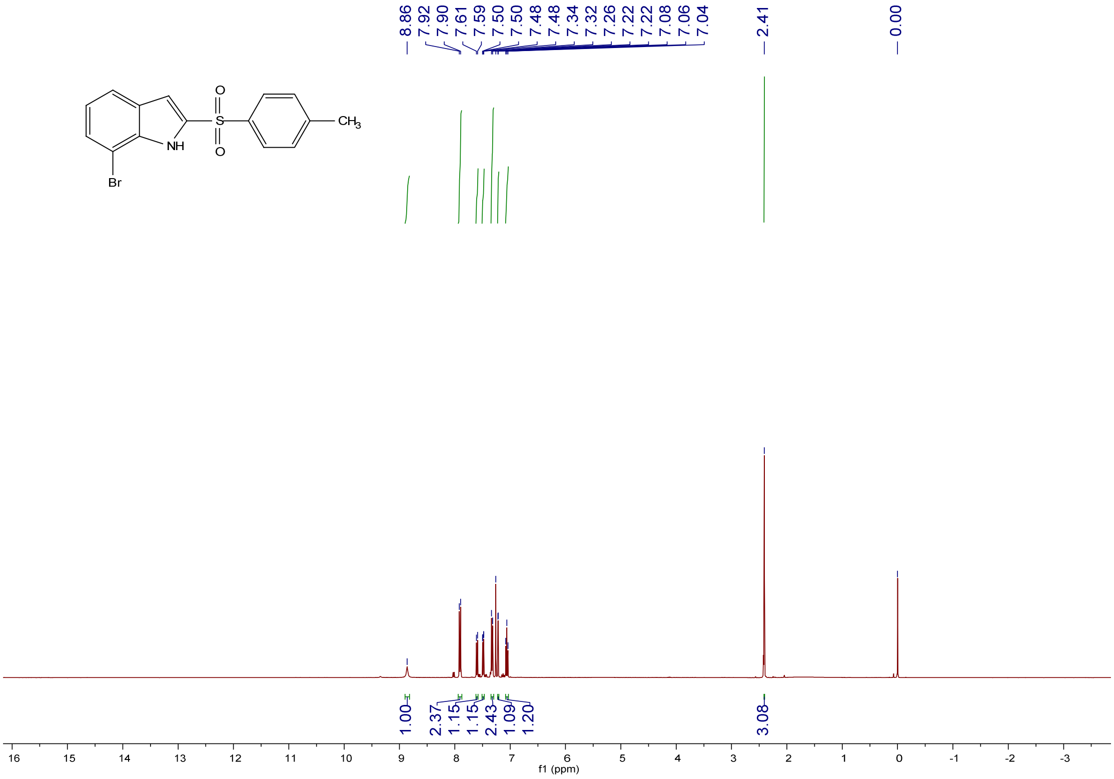

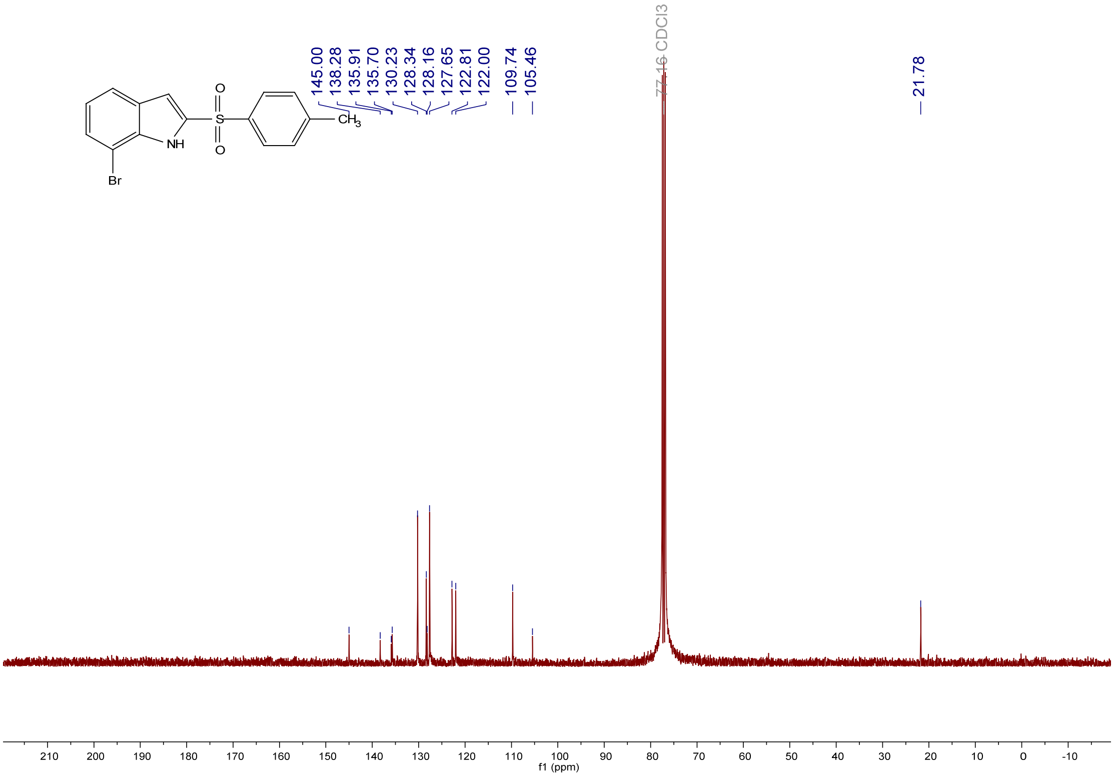

2-methyl-3-tosyl-1H-indole (3n)

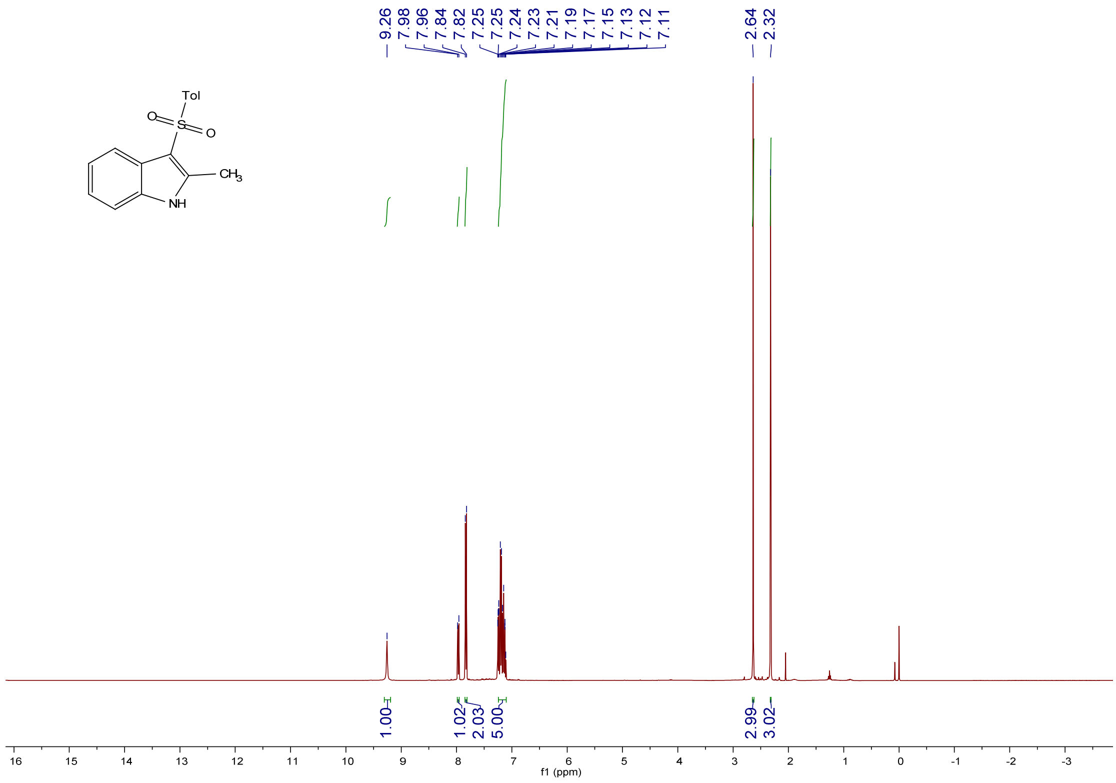

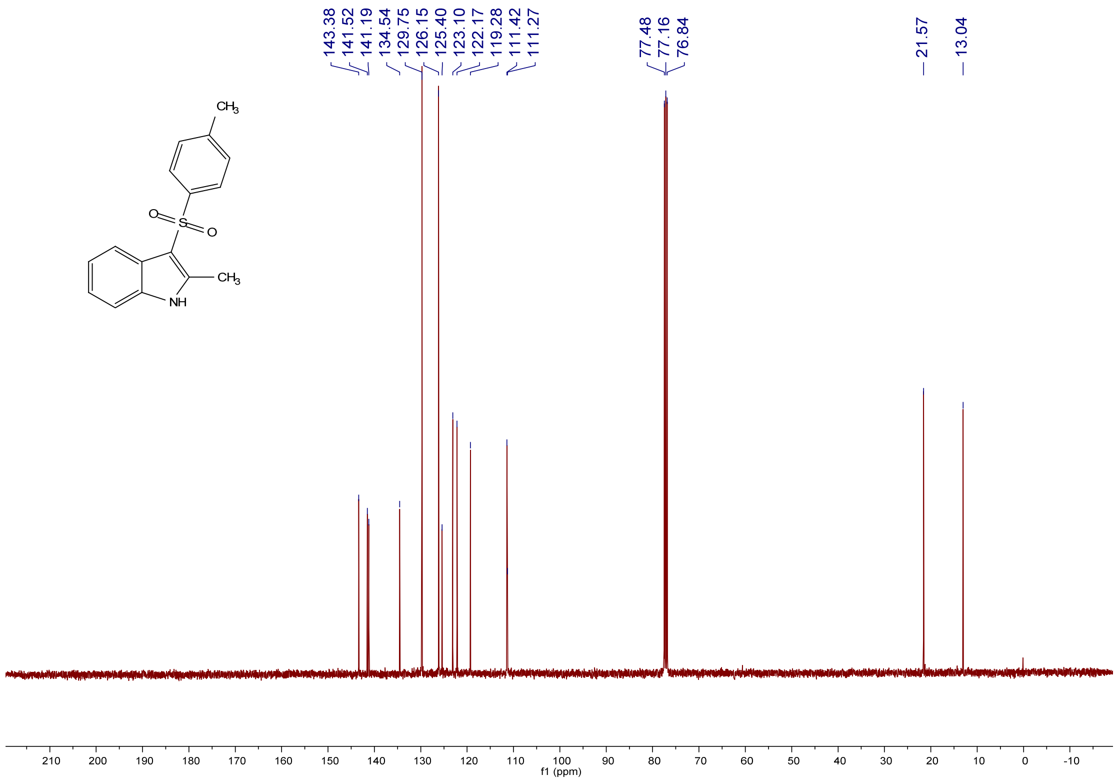

2-(phenylsulfonyl)-1H-indole (3o)

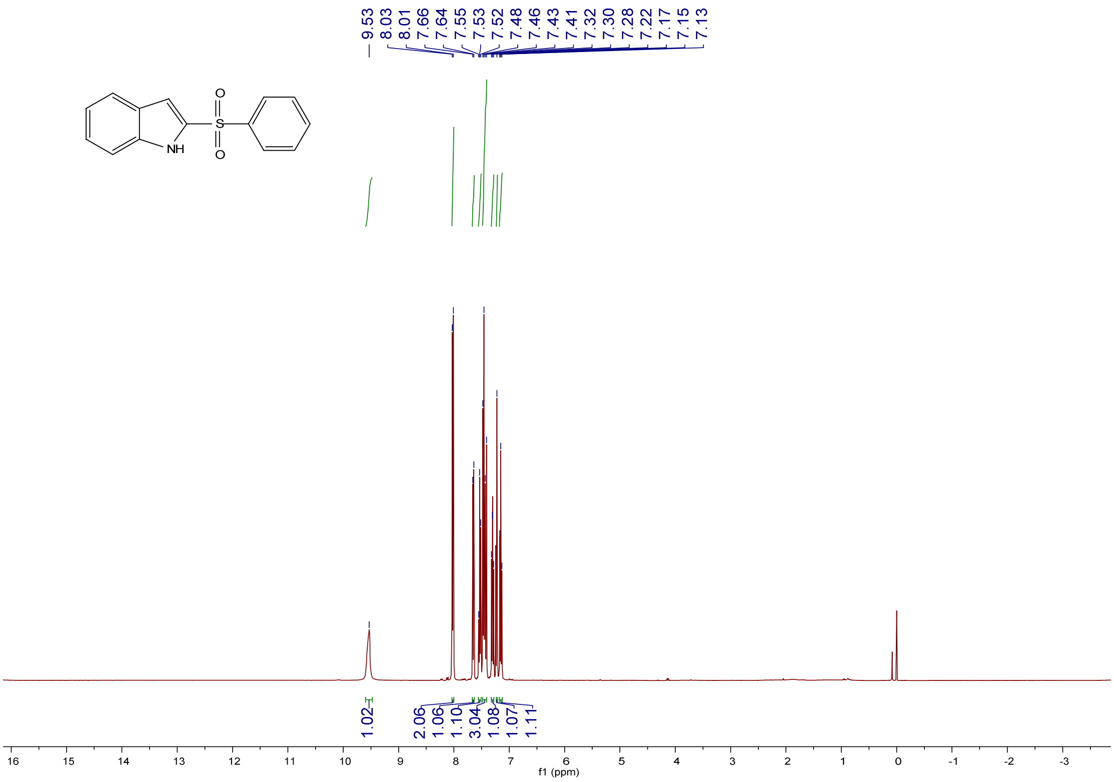

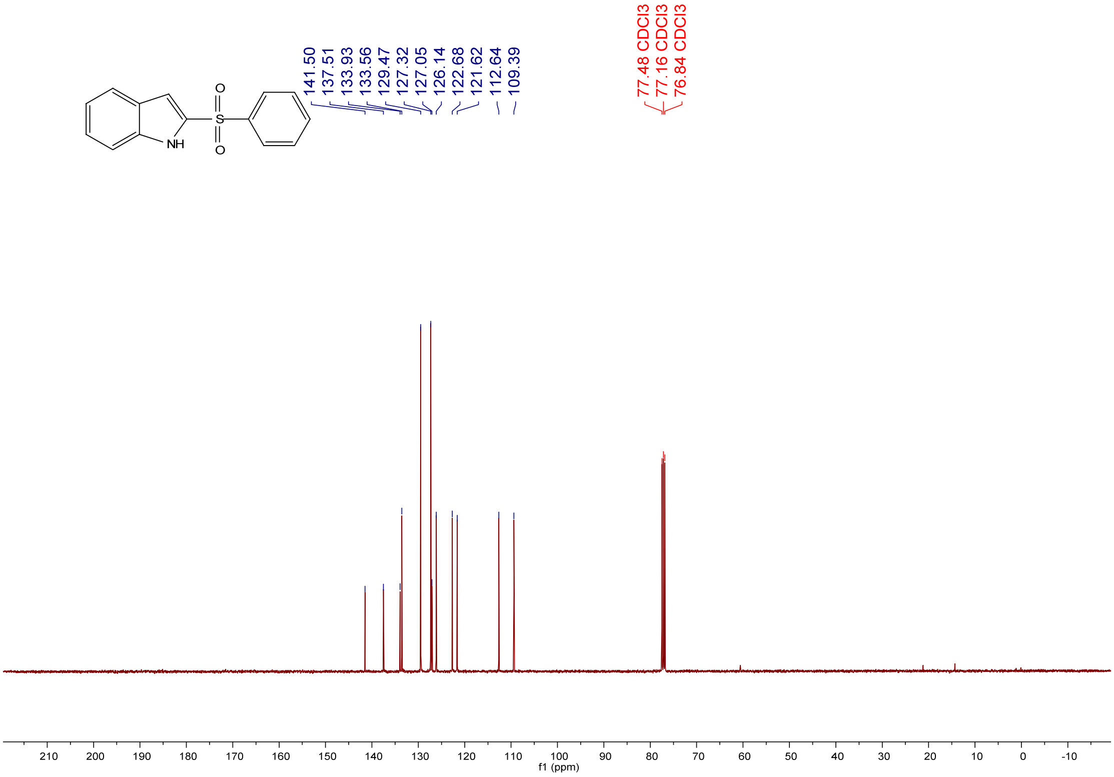

2-((4-fluorophenyl)sulfonyl)-1H-indole (3p)

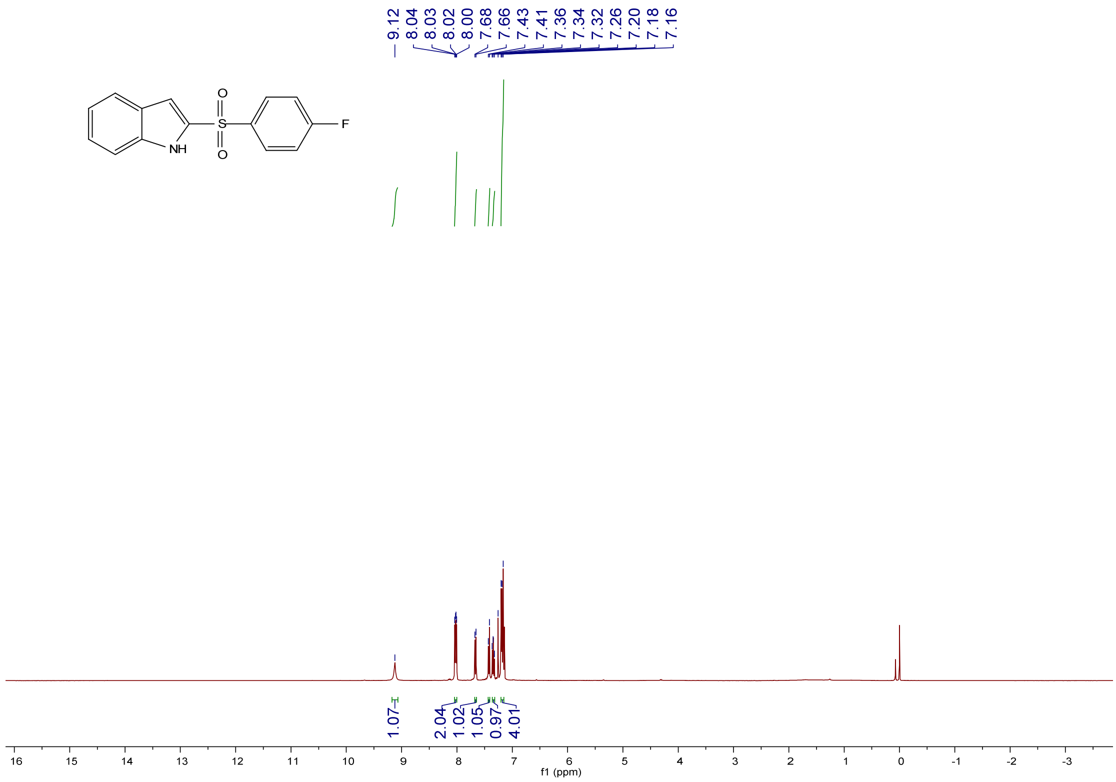

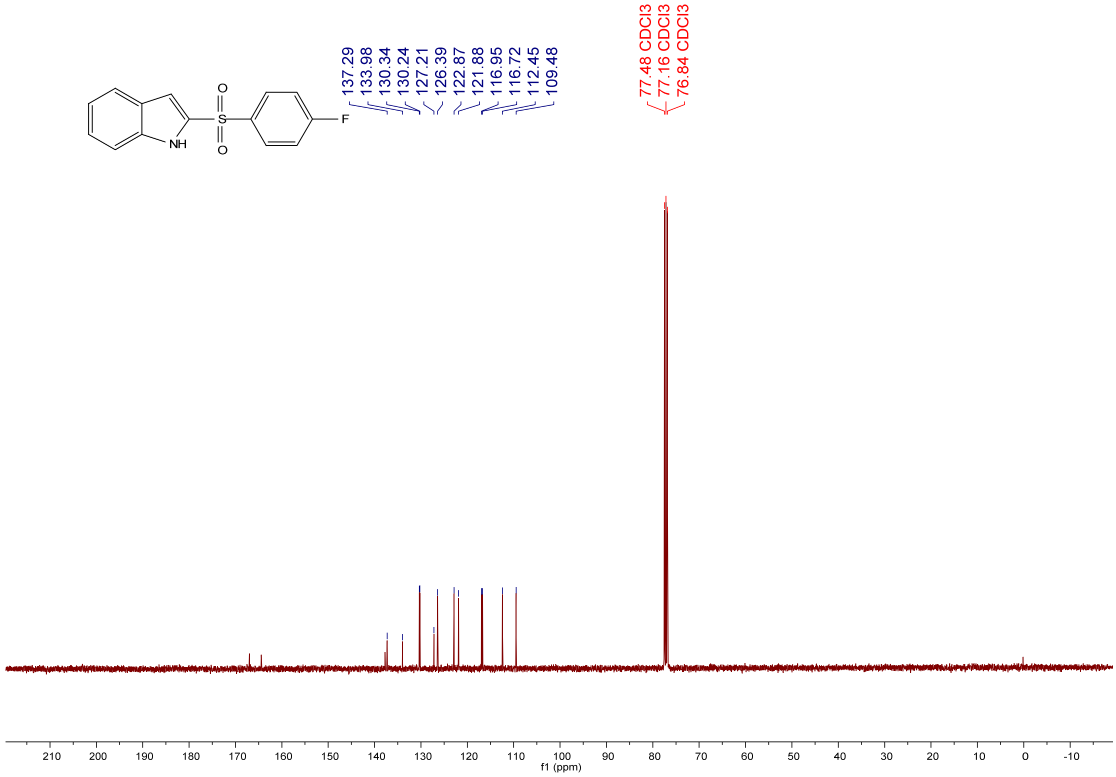

2-((4-chlorophenyl)sulfonyl)-1H-indole (3q)

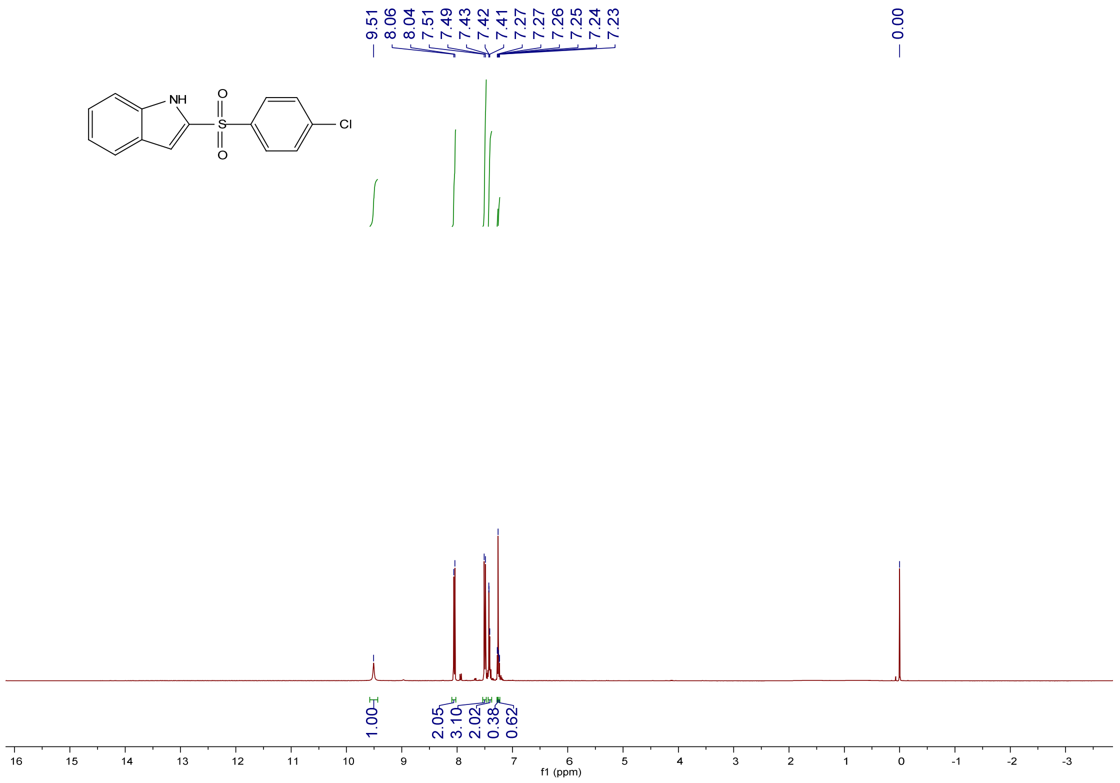

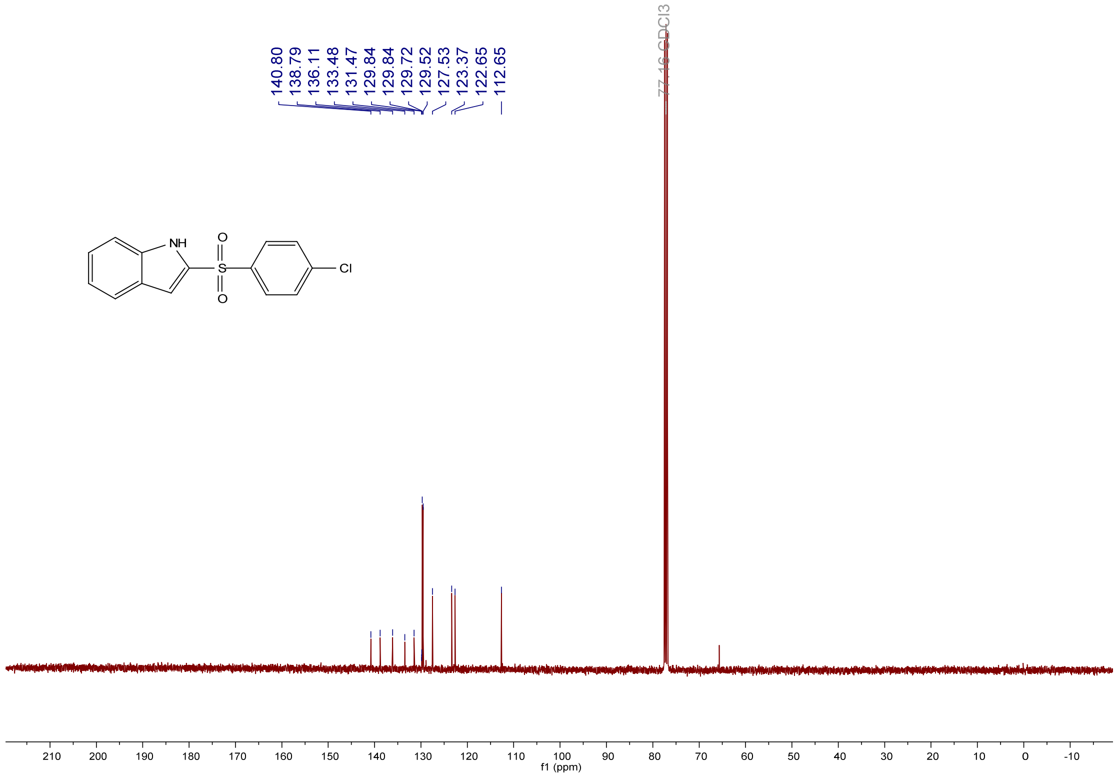

2-((4-bromophenyl)sulfonyl)-1H-indole (3r)

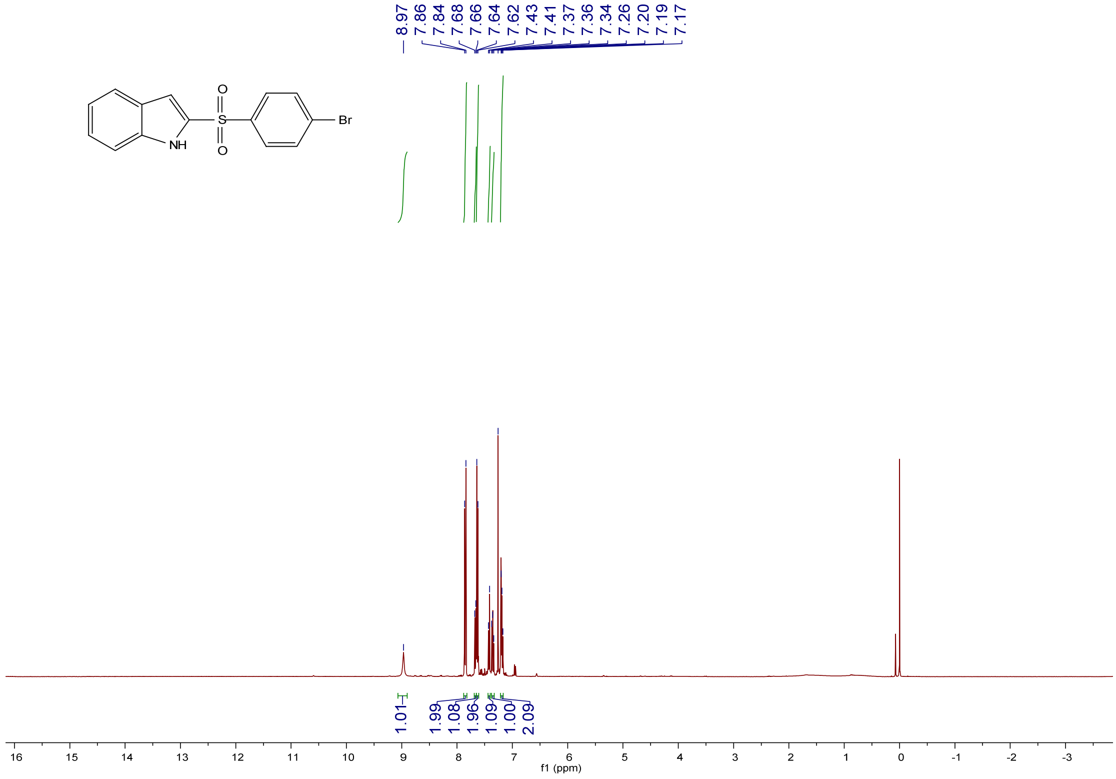

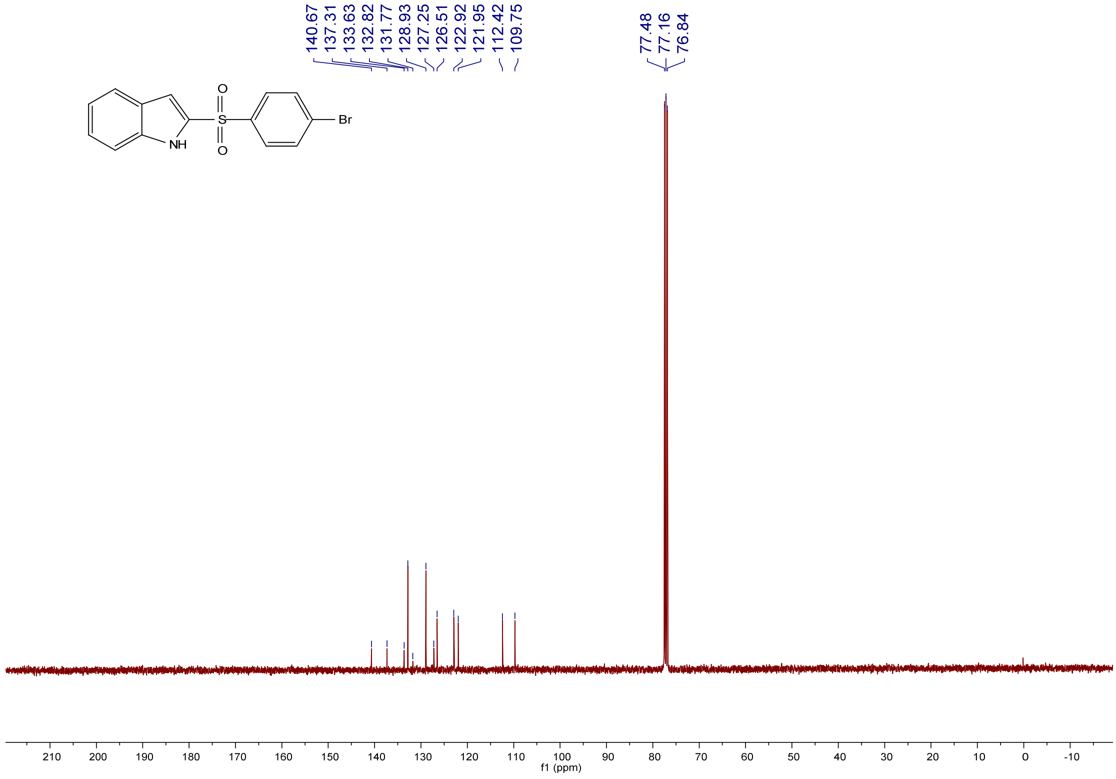

2-((4-(trifluoromethyl)phenyl)sulfonyl)-1H-indole (3s)

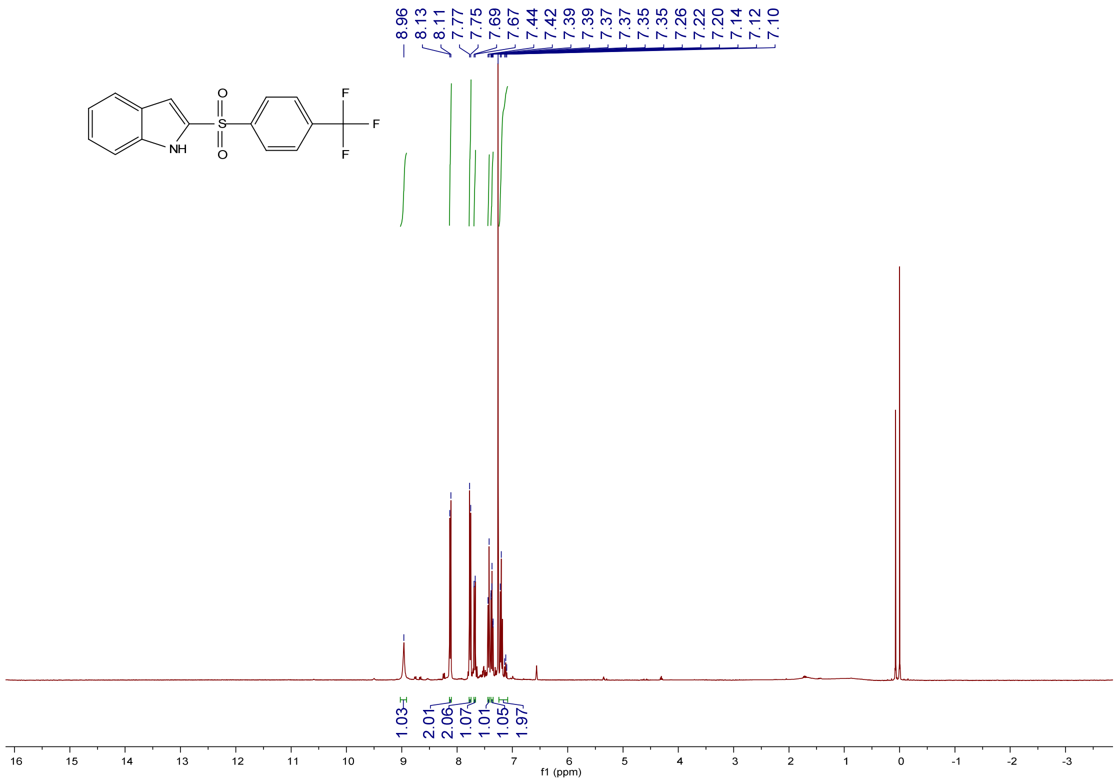

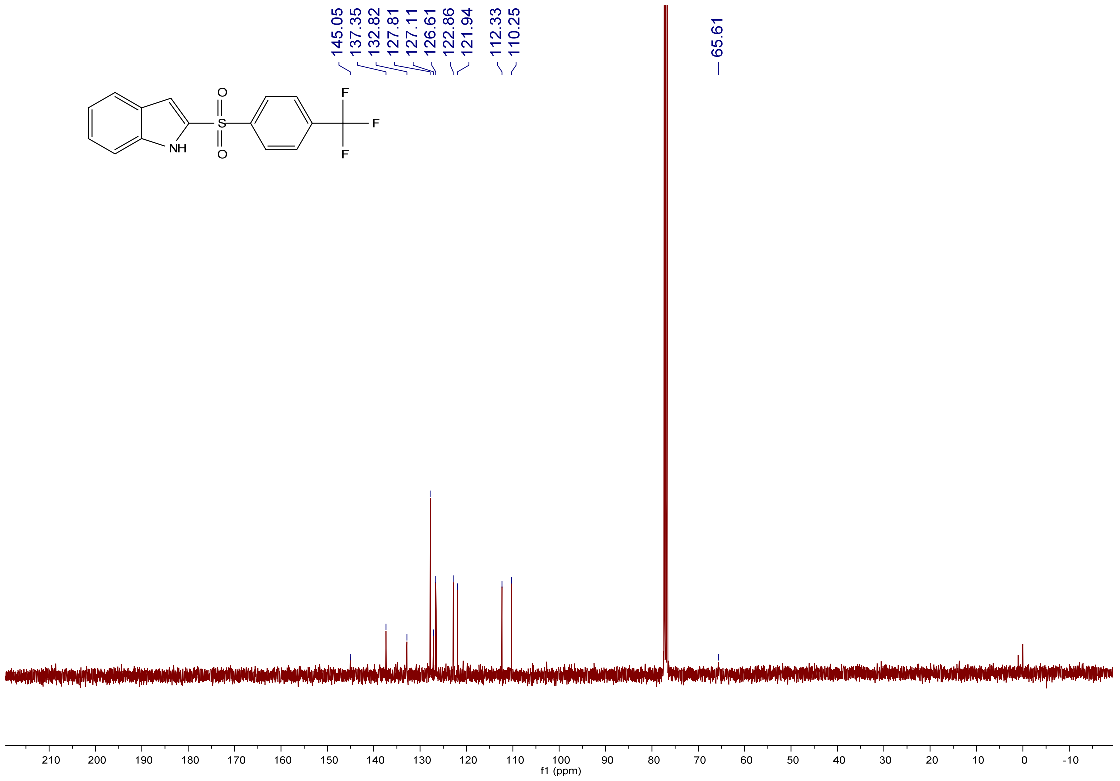

2-((4-nitrophenyl)sulfonyl)-1H-indole (3t)

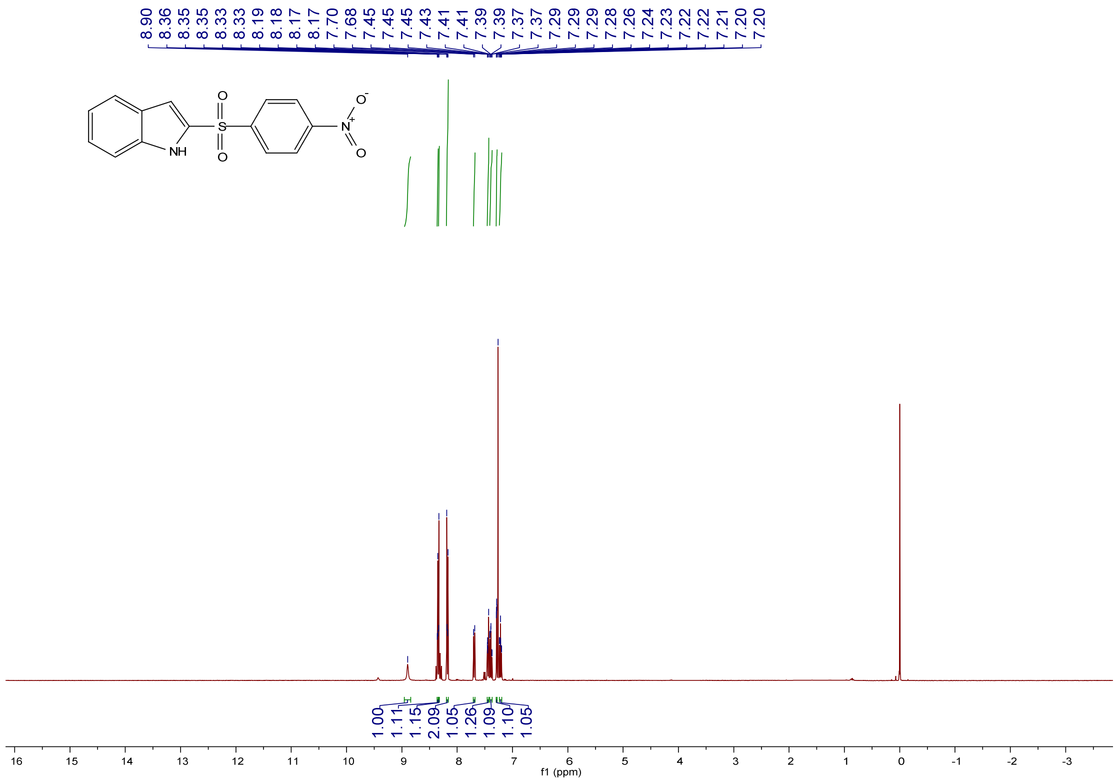

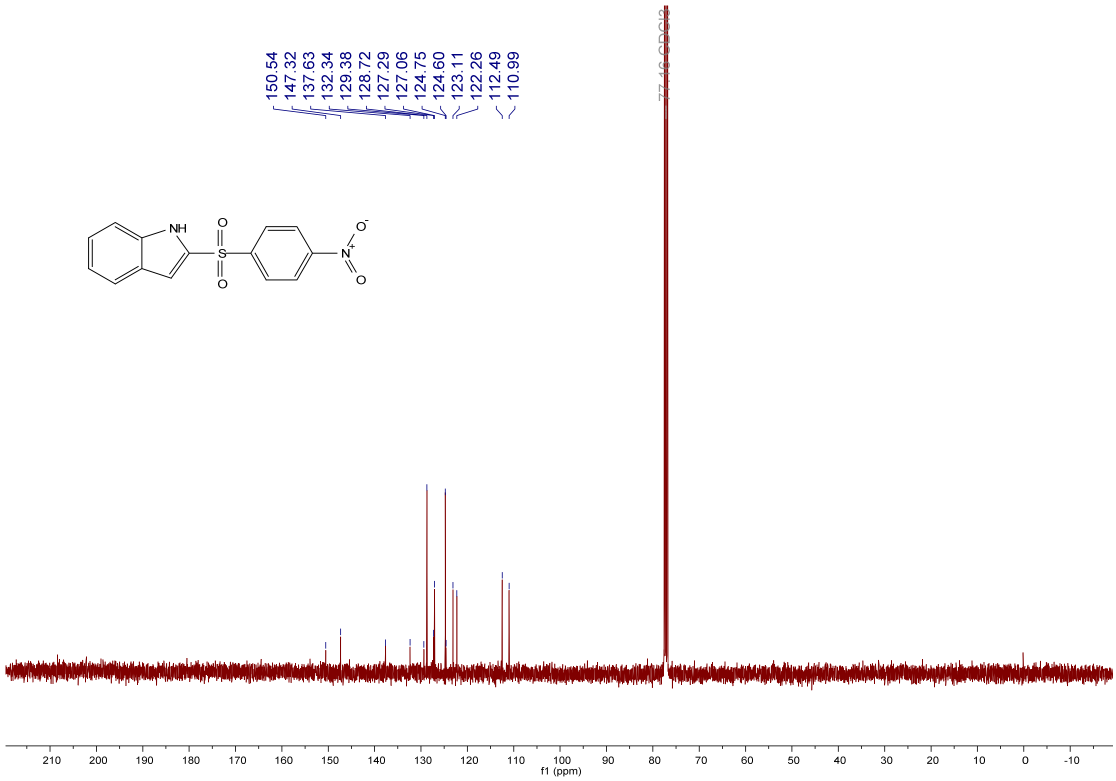

2-((4-(tert-butyl)phenyl)sulfonyl)-1H-indole(3u)

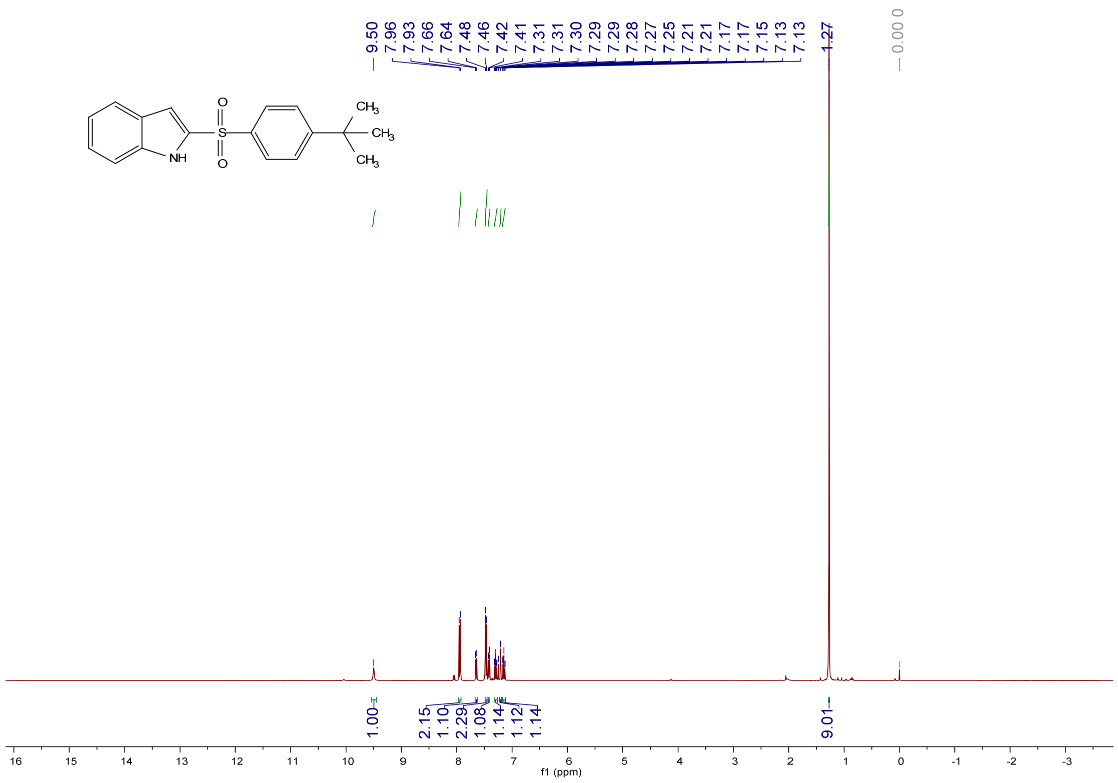

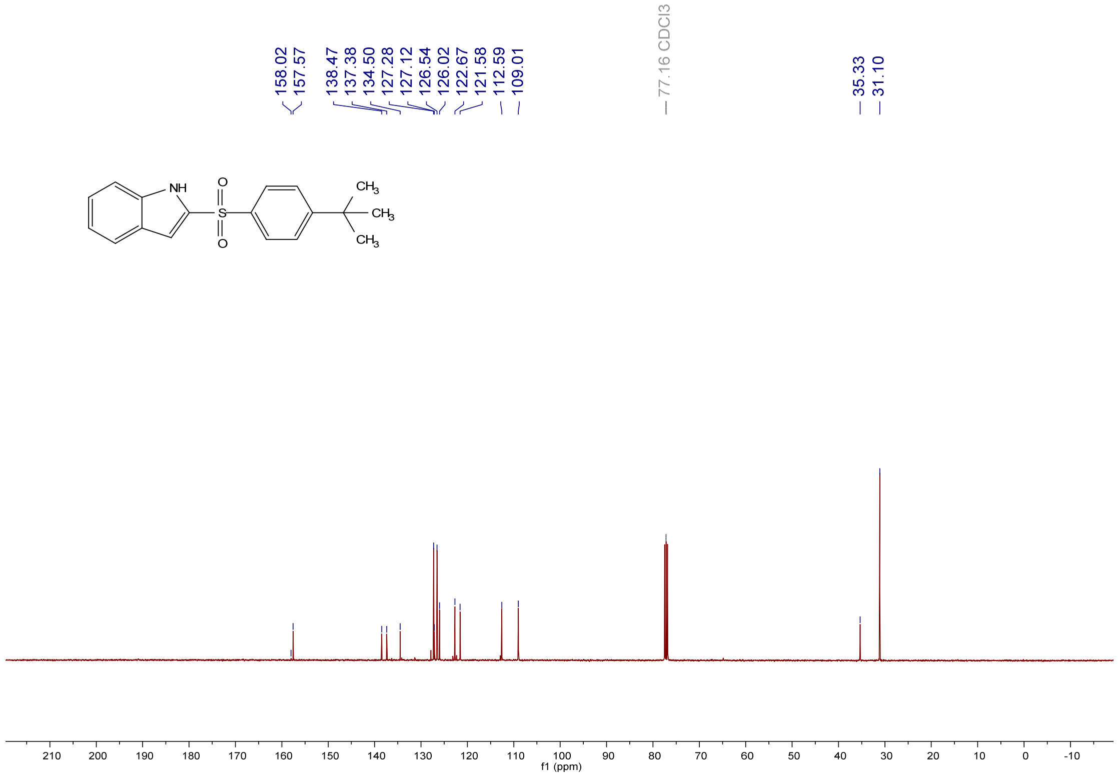

2-((4-methoxyphenyl)sulfonyl)-1H-indole (3v)

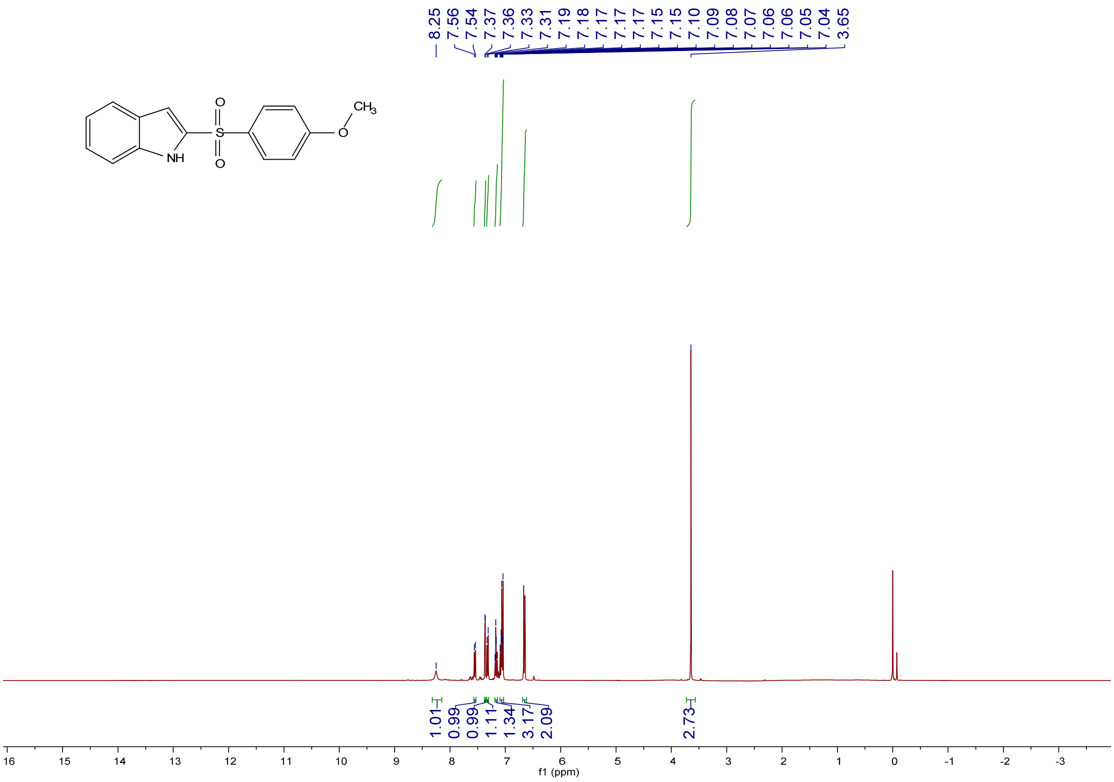

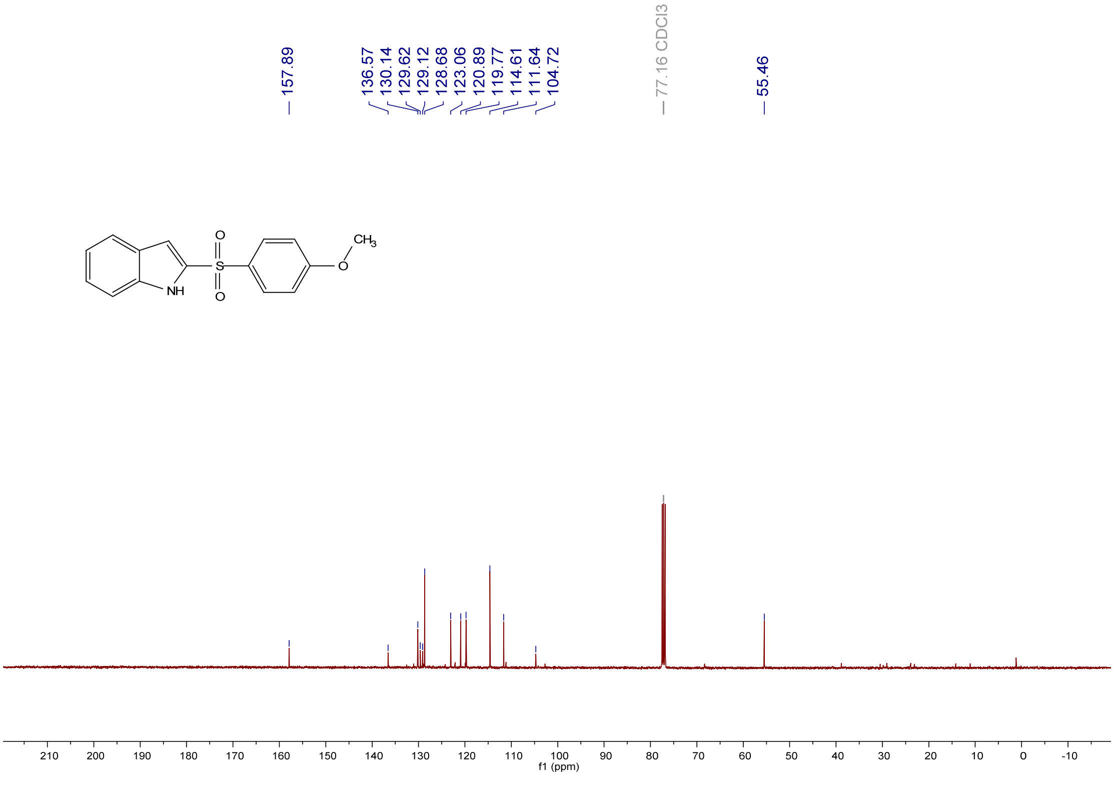

2-(naphthalen-2-ylsulfonyl)-1H-indole (3w)

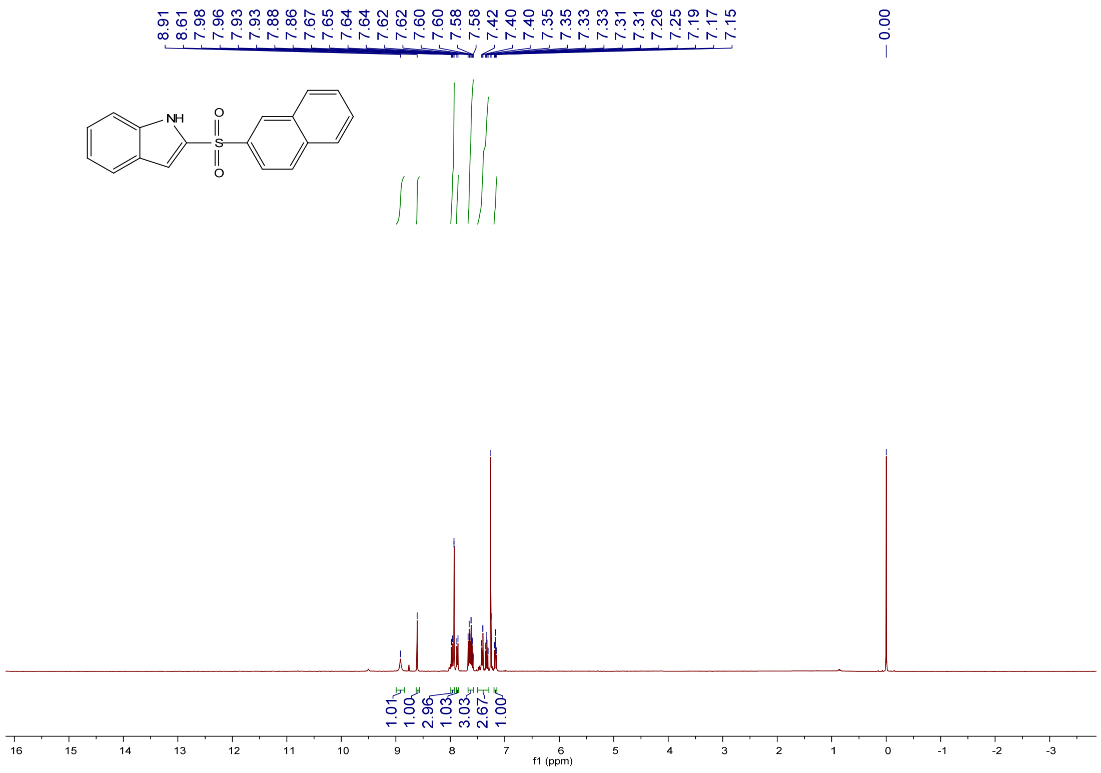

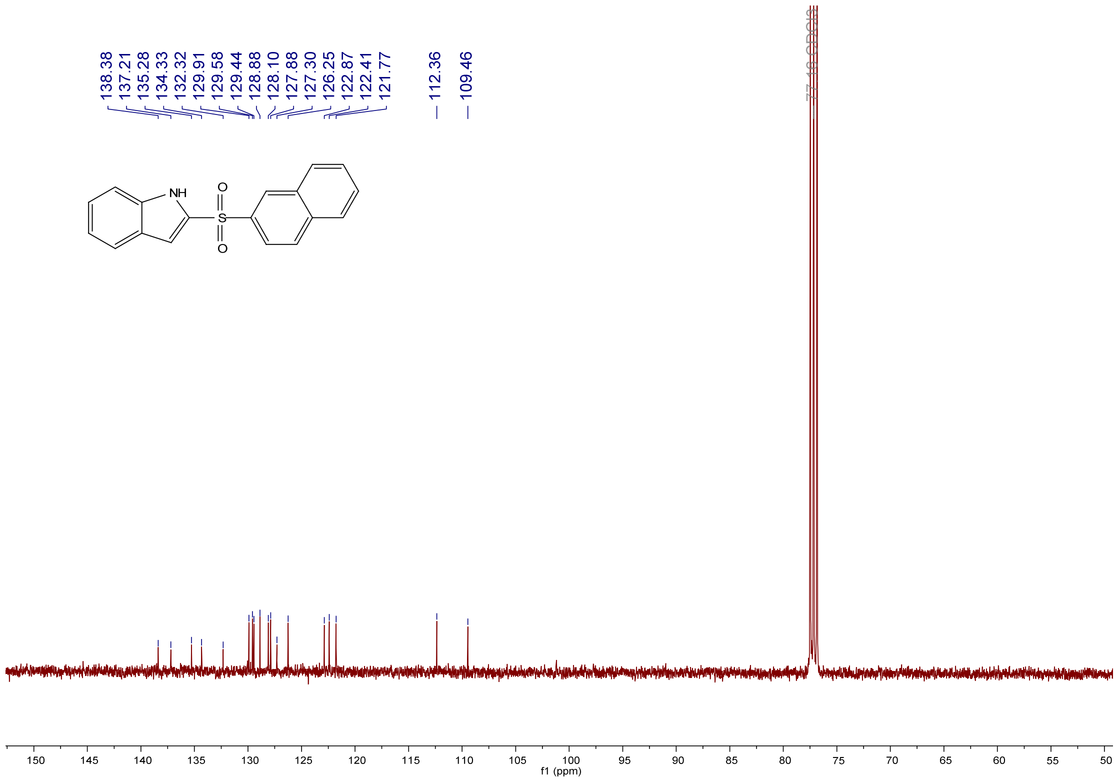

1-methyl-2-(phenylsulfonyl)-1H-pyrrole (5a)

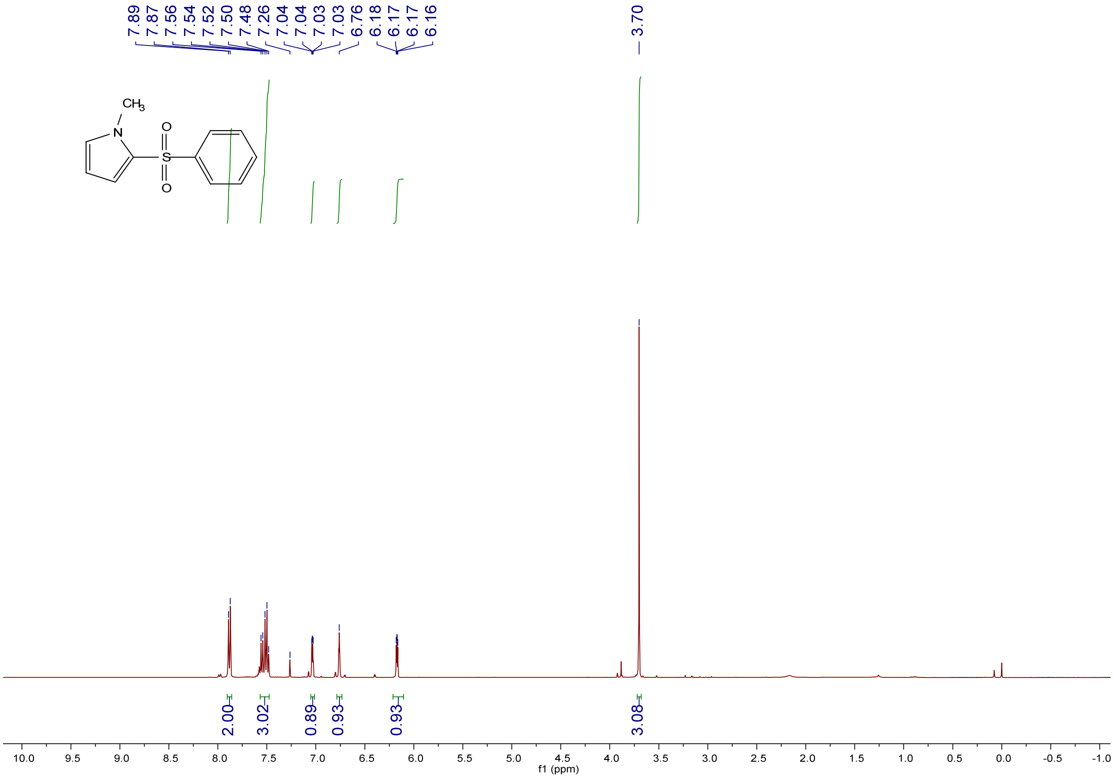

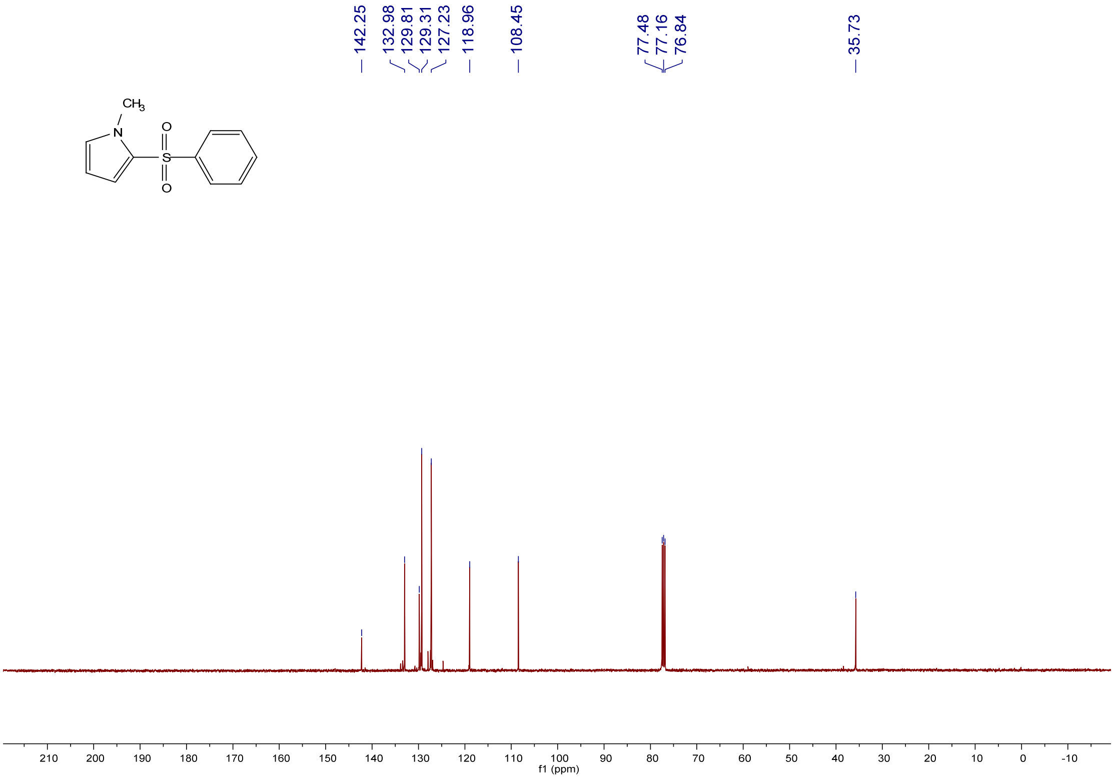

1-methyl-2-tosyl-1H-pyrrole (5b)

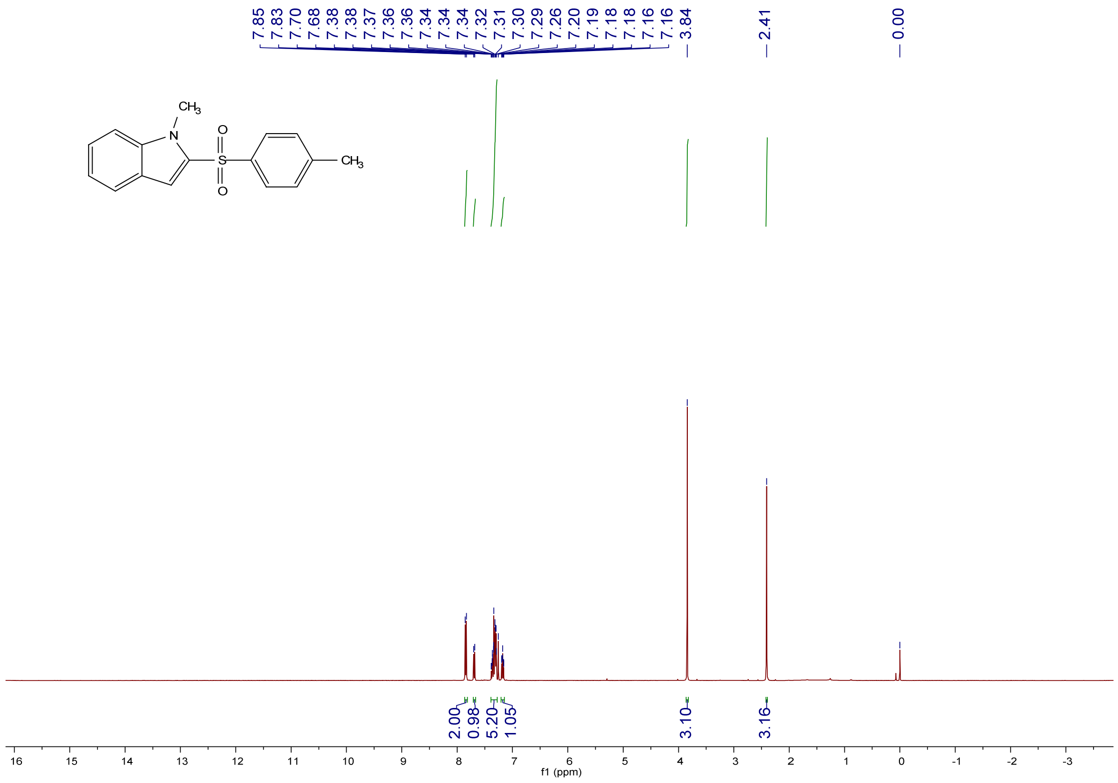

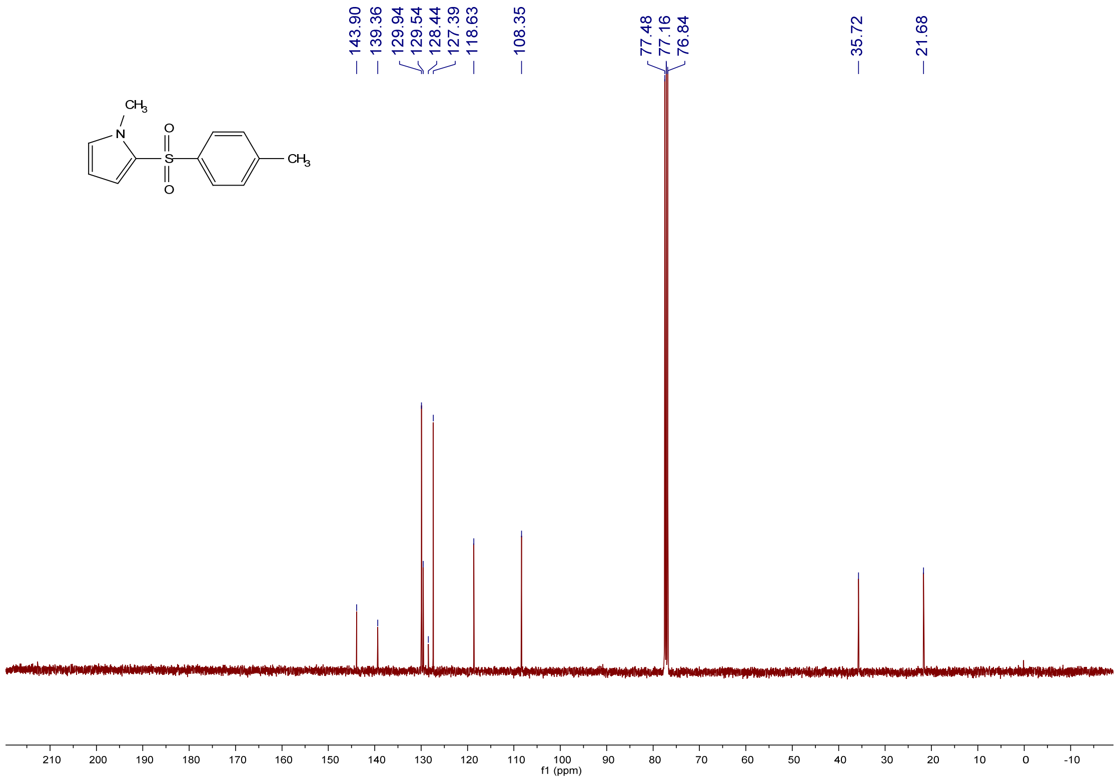

2-tosyl-1H-pyrrole (5b′)

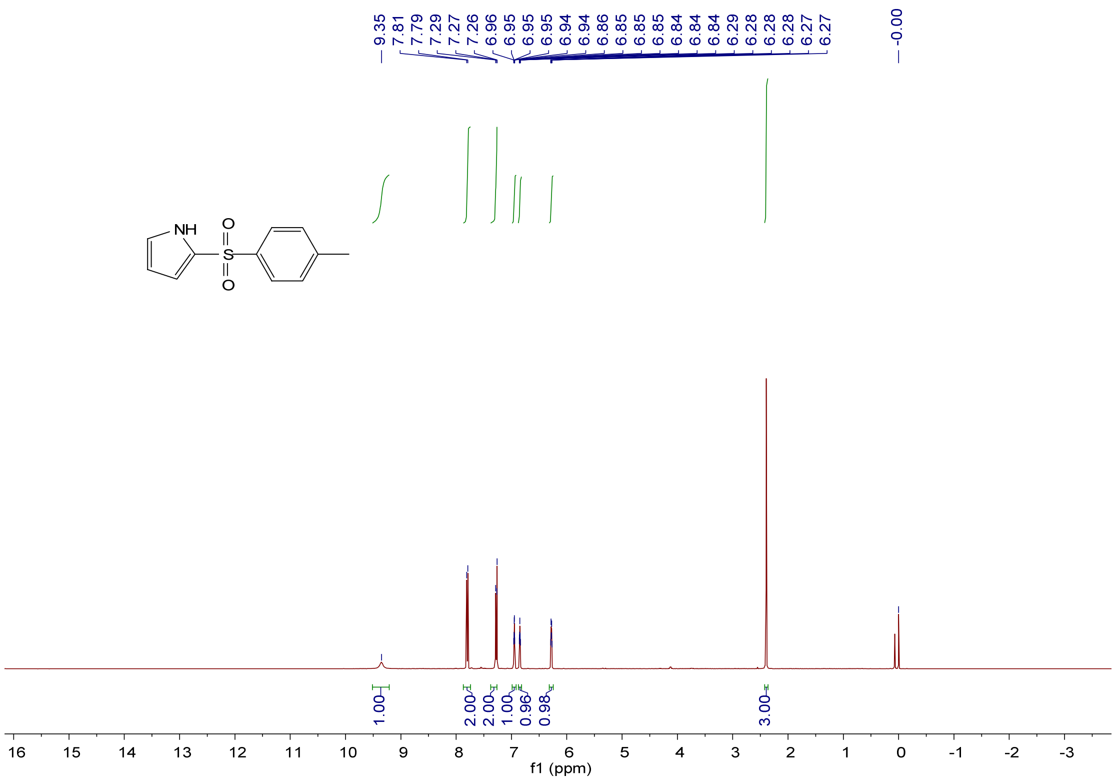

2-((4-(tert-butyl)phenyl)sulfonyl)-1-methyl-1H-pyrrole (5c)

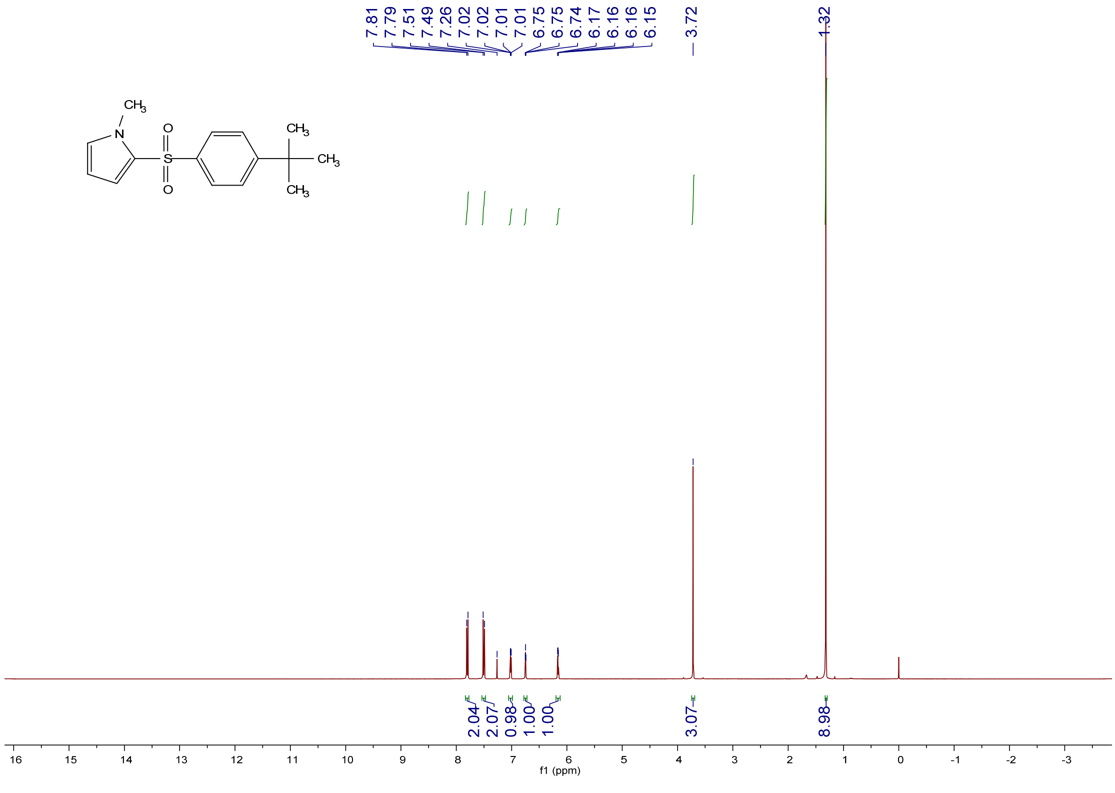

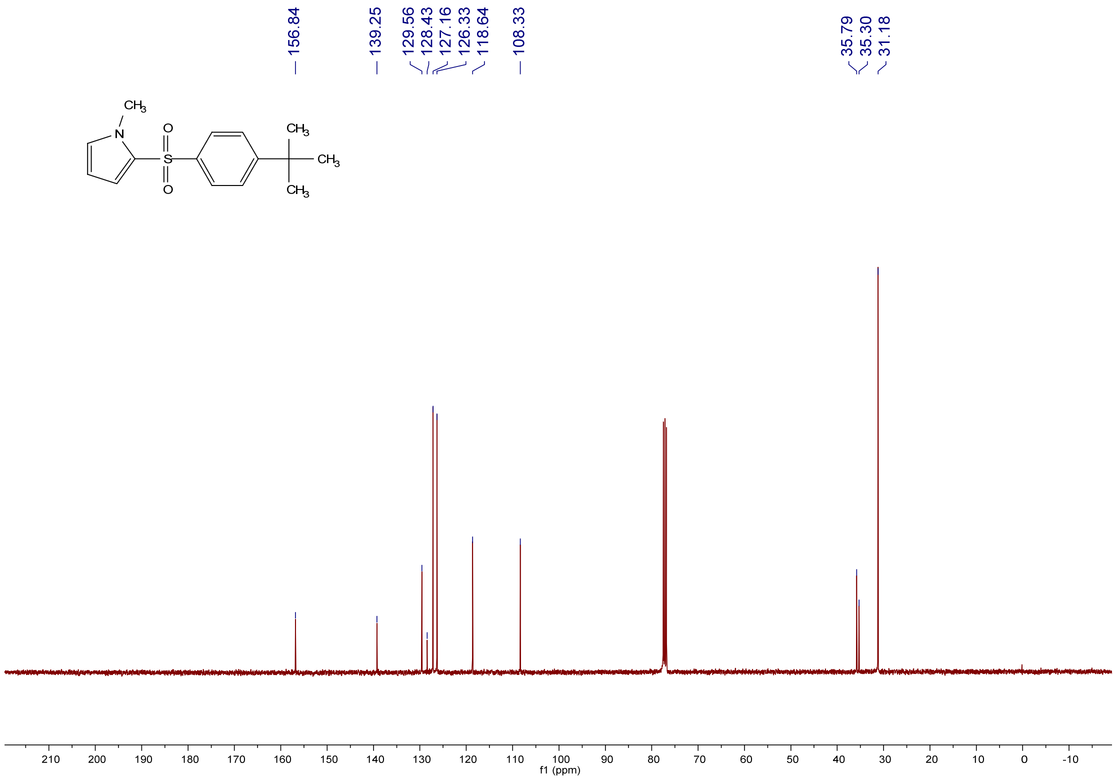

2-((4-fluorophenyl)sulfonyl)-1-methyl-1H-pyrrole (5d)

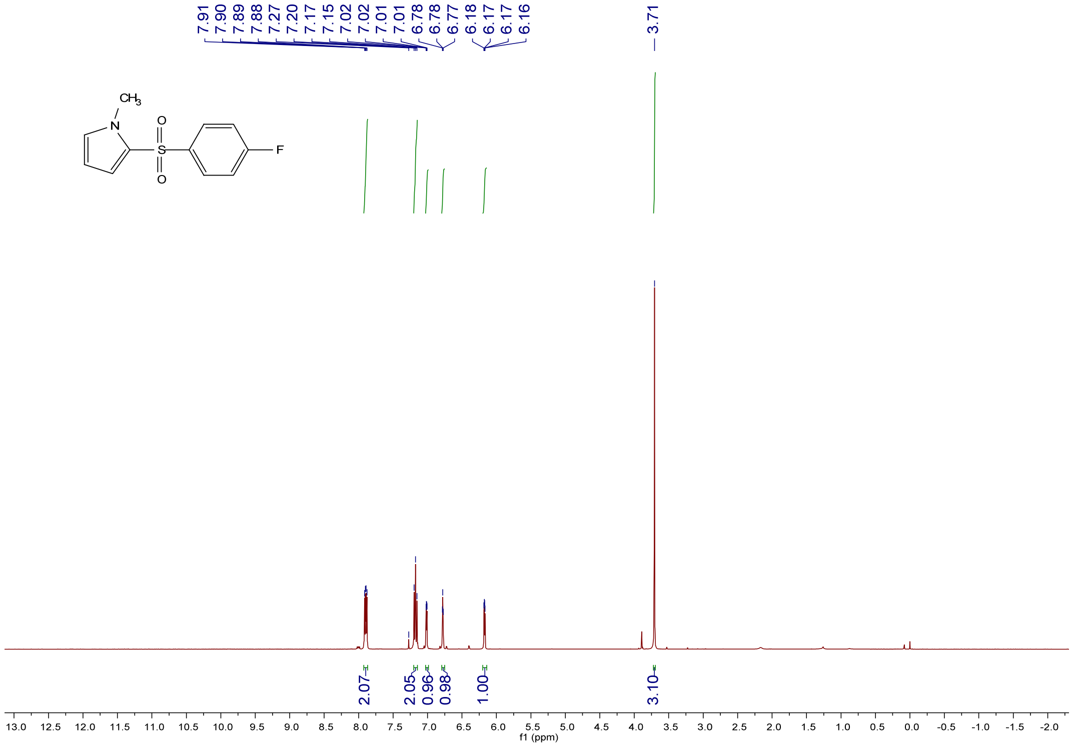

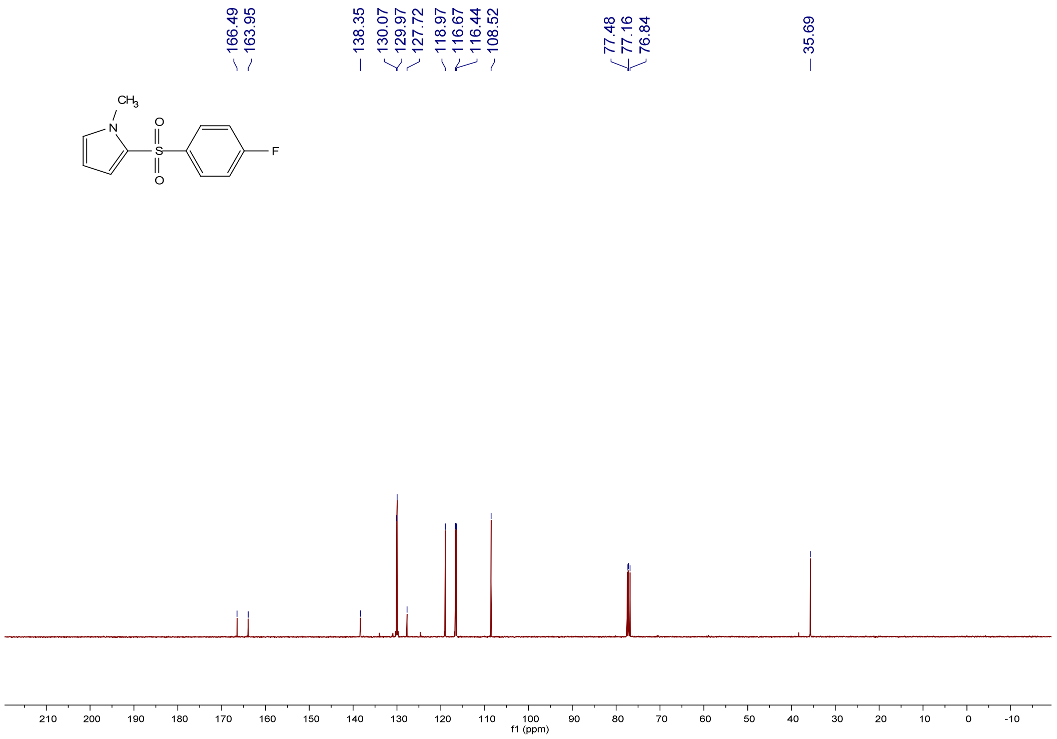

2-((4-chlorophenyl)sulfonyl)-1-methyl-1H-pyrrole (5e)

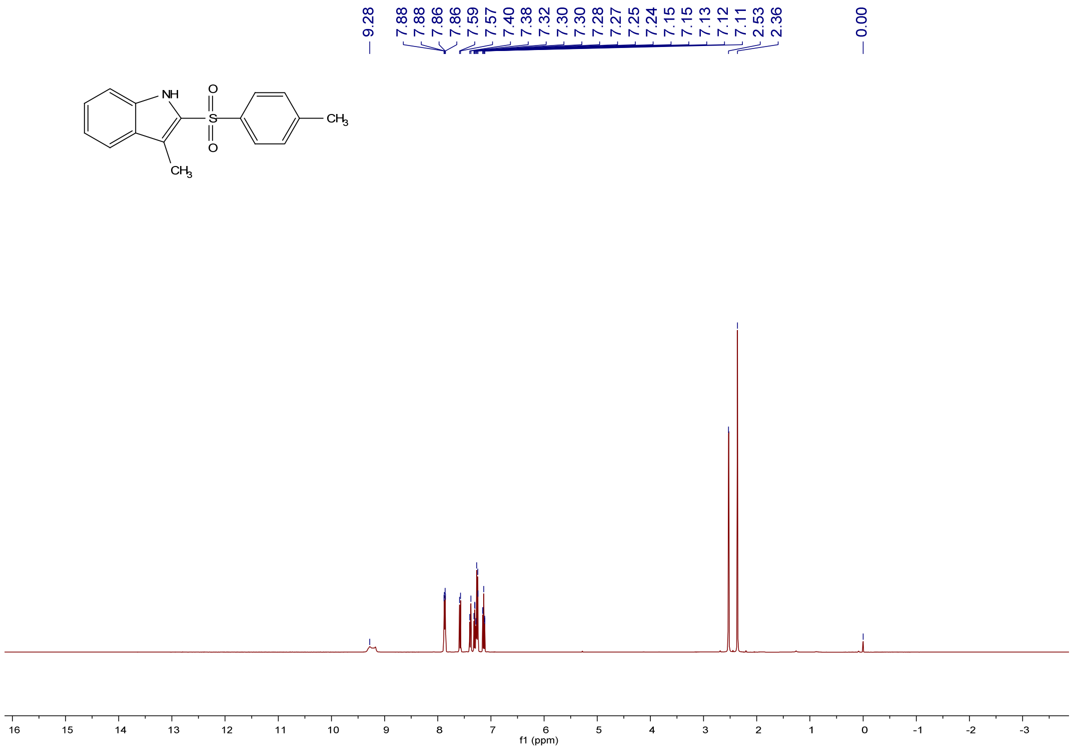

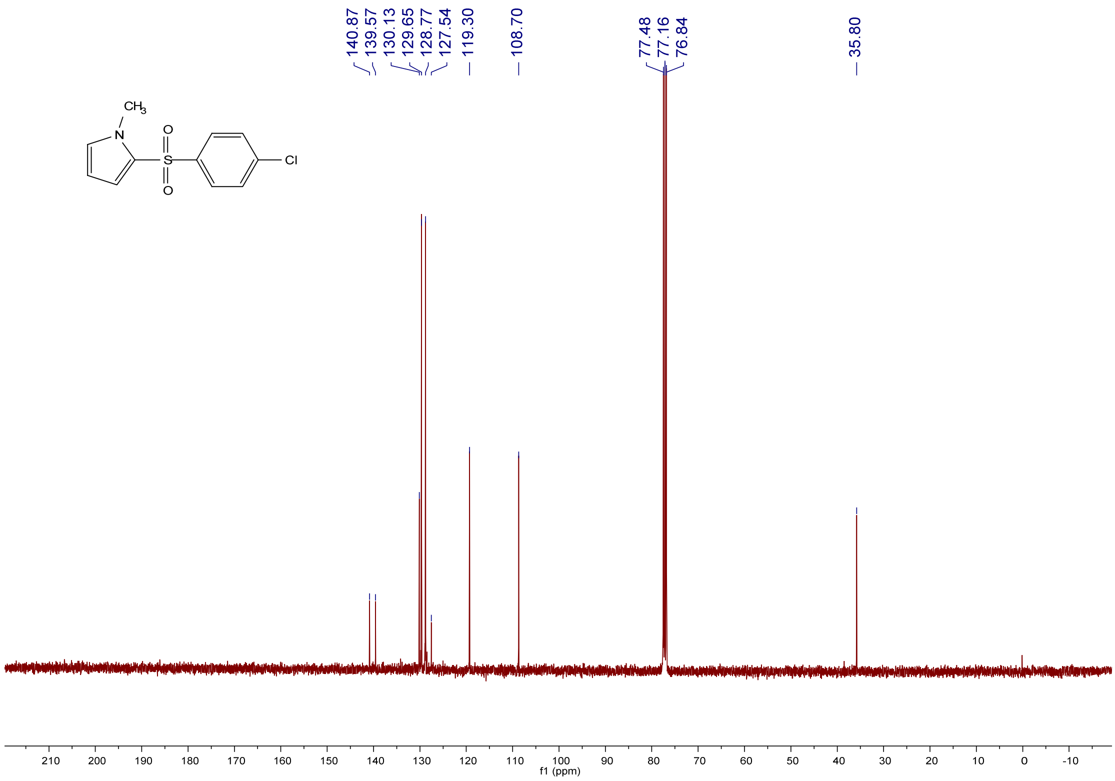

2-((4-bromophenyl)sulfonyl)-1-methyl-1H-pyrrole (5f)

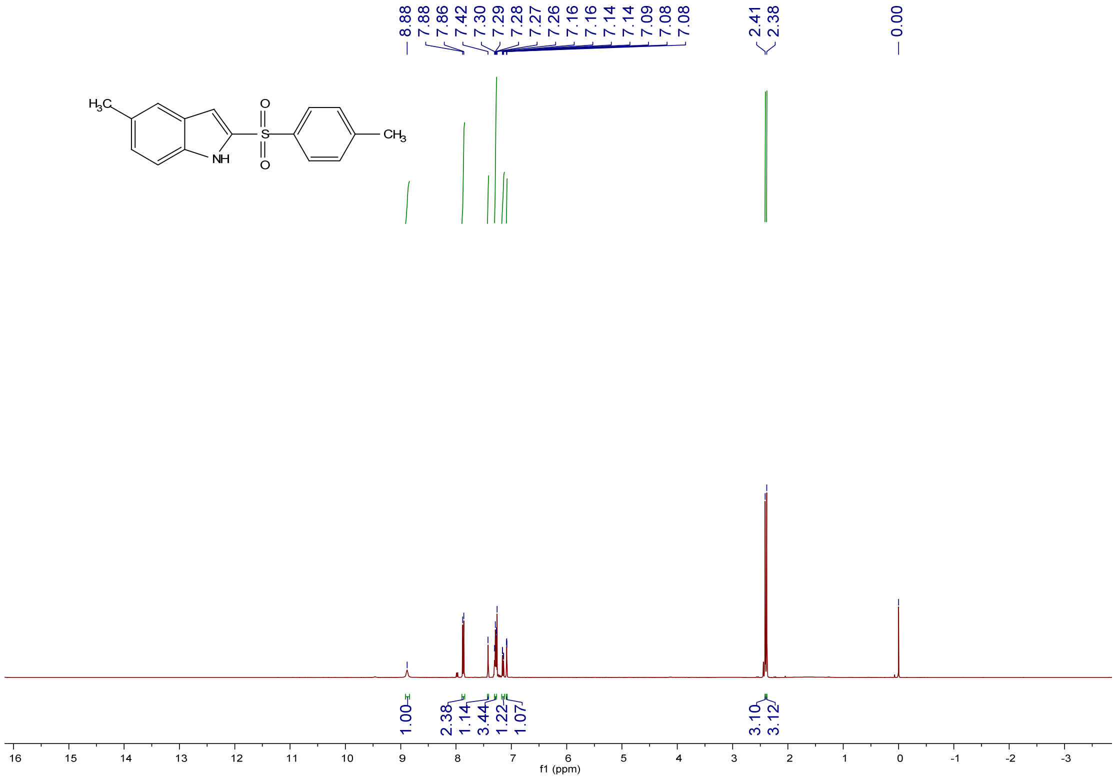

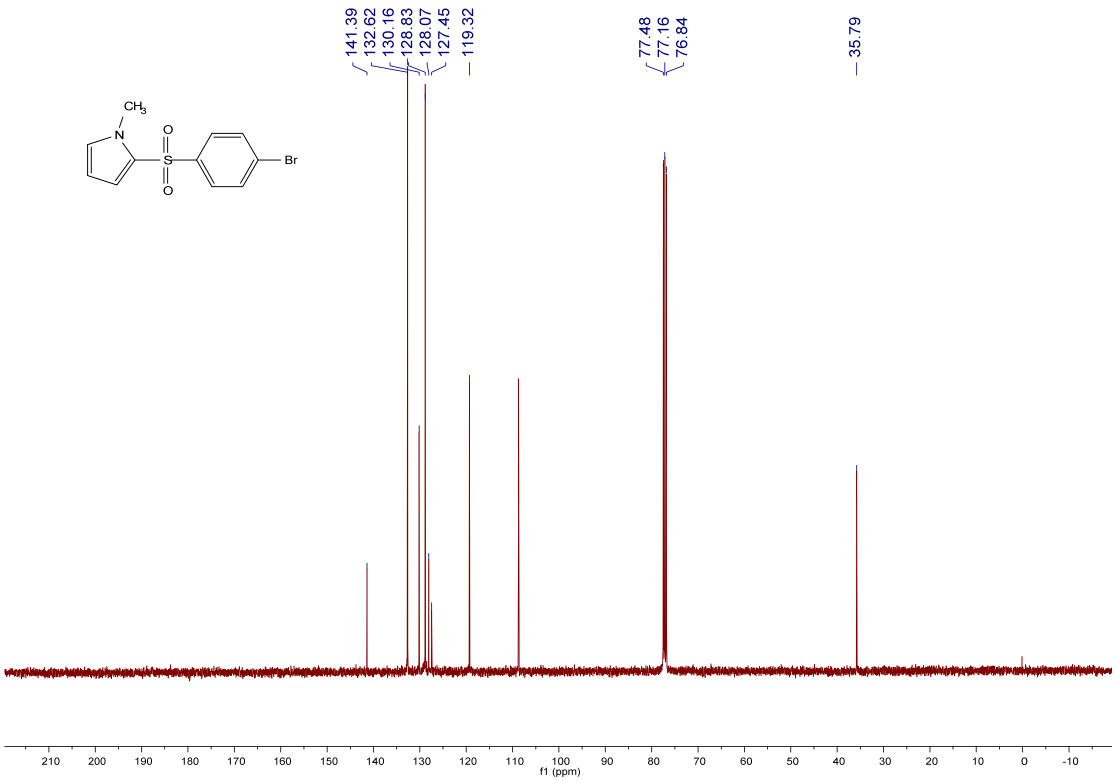

1-methyl-2-((4-(trifluoromethyl)phenyl)sulfonyl)-1H-pyrrole (5g)

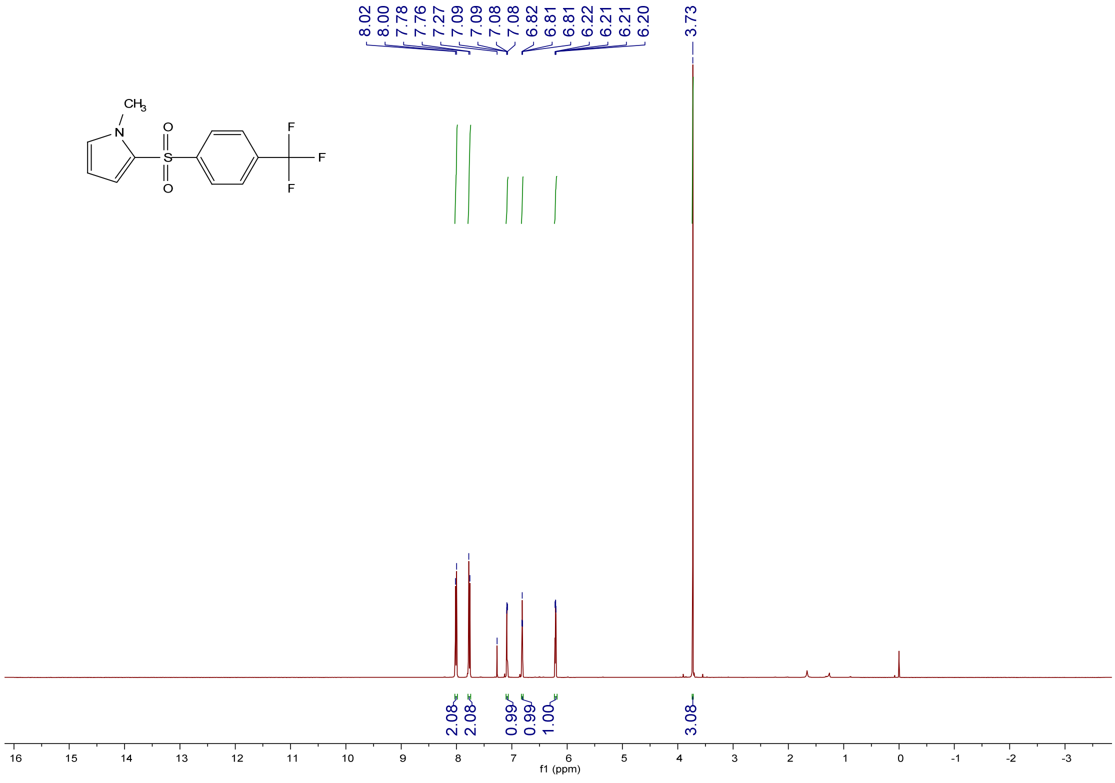

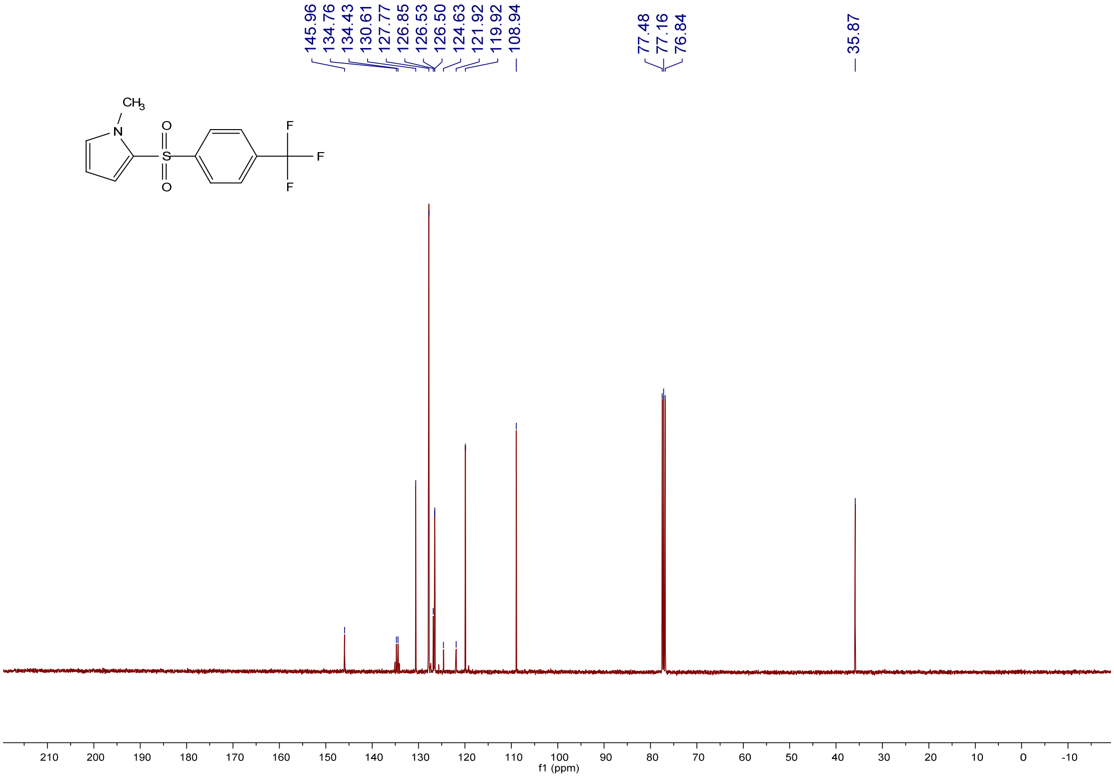

2-((3-bromophenyl)sulfonyl)-1-methyl-1H-pyrrole (5h)

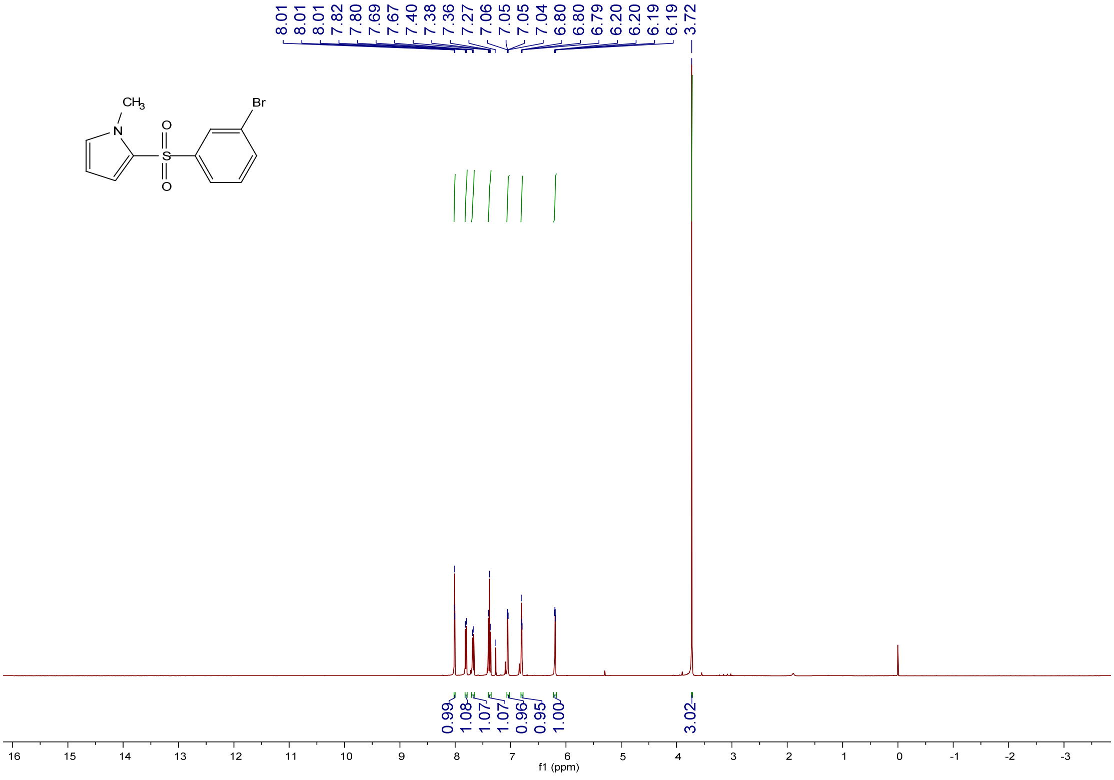

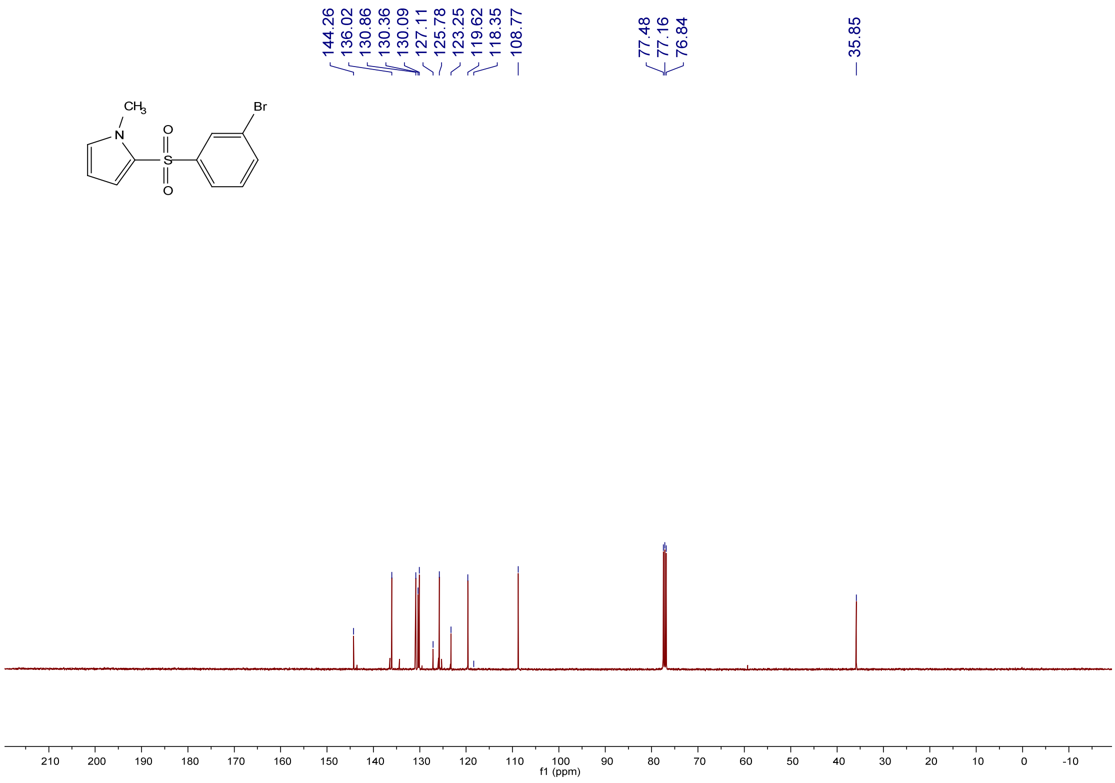

1-methyl-2-(naphthalen-2-ylsulfonyl)-1H-pyrrole (5i)

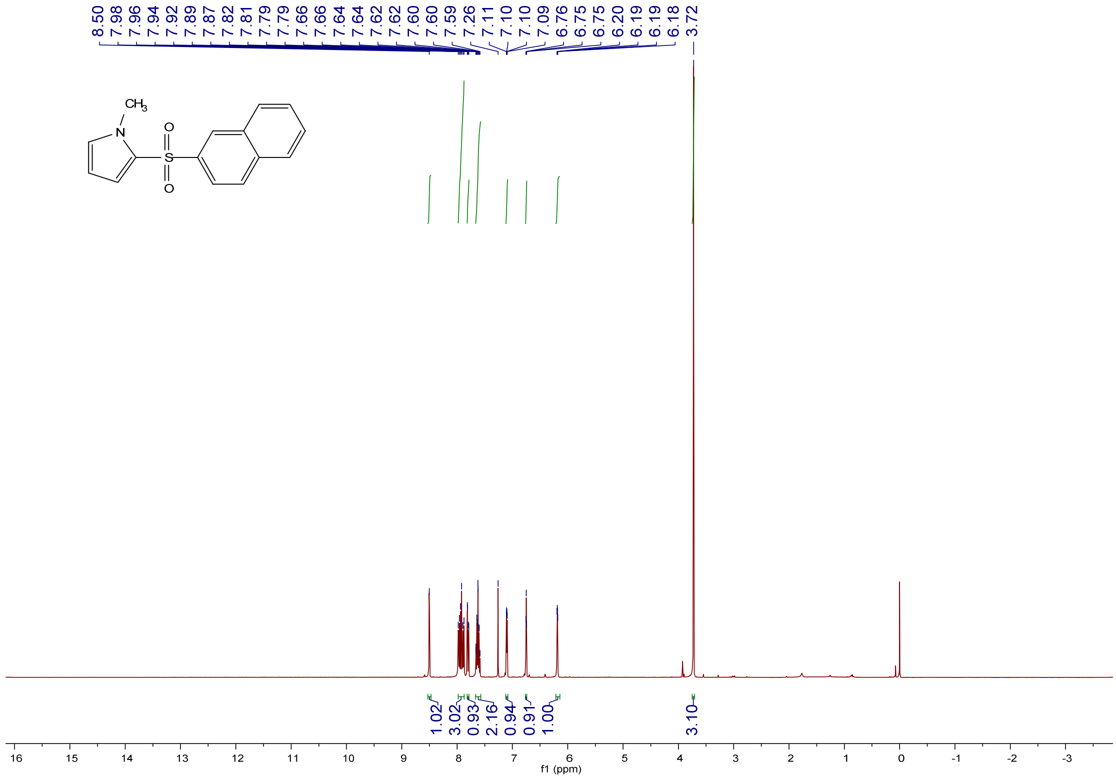

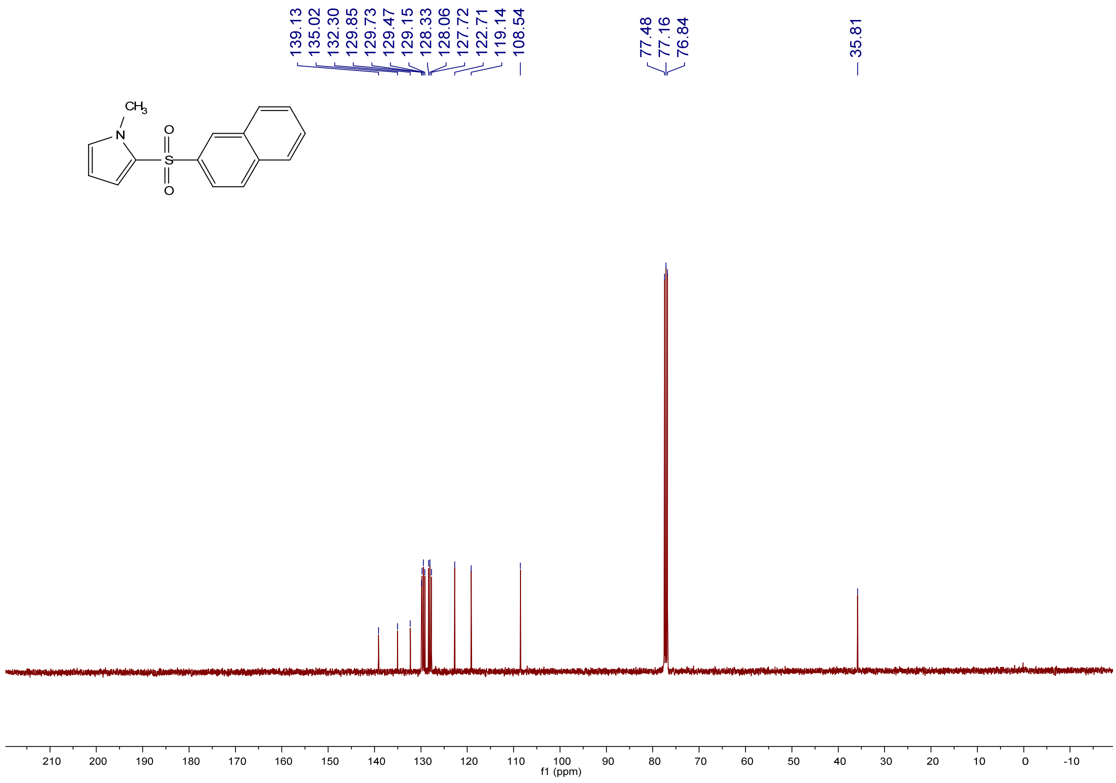
139.13
135.02
132.30
129.85
129.73
129.47
129.15
128.33
128.06
127.72
122.71
119.14
108.54
77.48
77.16
76.84
35.81
CH3
N
O
S
O
210 200 190 180 170 160 150 140 130 120 110 100 90 80 70 60 50 40 30 20 10 0 -10
f1 (ppm)
